# A comprehensive analysis of the germline and expressed TCR repertoire in White Peking duck

**DOI:** 10.1038/srep41426

**Published:** 2017-01-30

**Authors:** Zhi Yang, Yi Sun, Yonghe Ma, Zhenrong Li, Yu Zhao, Liming Ren, Haitang Han, Yunliang Jiang, Yaofeng Zhao

**Affiliations:** 1 State Key Laboratory of Agrobiotechnology, College of Biological Sciences, China Agricultural University, Beijing, 100193, China; 2 Beijing Advanced Innovation Center for Food Nutrition and Human Health, College of Food Science and Nutritional Engineering, China Agricultural University, Beijing, 100193, China; 3 Shandong Provincial Key Laboratory of Animal Biotechnology and Disease Control and Prevention, College of Animal Science and Veterinary Medicine, Shandong Agricultural University, Taian, 271018, China; 4 National Laboratory of Biomacromolecules, Institute of Biophysics, Chinese Academy of Sciences, Beijing, 100101, China

## Abstract

Recently, many immune-related genes have been extensively studied in ducks, but relatively little is known about their TCR genes. Here, we determined the germline and expressed repertoire of TCR genes in White Peking duck. The genomic organization of the duck TCRα/δ, TCRγ and unconventional TCRδ2 loci are highly conserved with their counterparts in mammals or chickens. By contrast, the duck TCRβ locus is organized in an unusual pattern, (Vβ)_n_-Dβ-(Jβ)_2_-Cβ1-(Jβ)_4_-Cβ2, which differs from the tandem-aligned clusters in mammals or the translocon organization in some teleosts. Excluding the first exon encoding the immunoglobulin domain, the subsequent exons of the two Cβ show significant diversity in nucleotide sequence and exon structure. Based on the nucleotide sequence identity, 49 Vα, 30 Vδ, 13 Vβ and 15 Vγ unique gene segments are classified into 3 Vα, 5 Vδ, 4 Vβ and 6 Vγ subgroups, respectively. Phylogenetic analyses revealed that most duck V subgroups, excluding Vβ1, Vγ5 and Vγ6, have closely related orthologues in chicken. The coding joints of all cDNA clones demonstrate conserved mechanisms that are used to increase junctional diversity. Collectively, these data provide insight into the evolution of TCRs in vertebrates and improve our understanding of the avian immune system.

Conventional T cell receptors (TCRs) are disulfide-linked heterodimers comprising either α and β chains or γ and δ chains. All four types of TCR chains are trans-membrane molecules that contain antigen-binding variable (V) domains and membrane-proximal constant (C) domains. The V domains of TCRβ and TCRδ are assembled via somatic recombination of variable (V), diversity (D) and joining (J) gene segments, whereas the rearranged V and J segments encode the V domains of TCRα and TCRγ^1^. Based on the combinations of TCR heterodimers, conventional T cells can be divided into two major lineages: αβ T cells and γδ T cells. The αβ T cells mainly assist in immunoglobulin (Ig) production and cytolytic T cell responses. Their αβTCR complexes bind to the peptide antigens presented by major histocompatibility complex (MHC) or MHC-like molecules^2^. By contrast, γδ T cells constitute a heterogeneous T cell population with multiple functions. Some γδTCR complexes can recognize antigens presented by MHC molecules, whereas other γδTCRs appear to bind directly to free antigens, similar to the recognition manner utilized by Igs^3^. The frequencies and physiological distributions of γδ T cells differ among diverse species. In adult humans, mice and dogs, γδ T cells make up less than 5% of the peripheral T cells (“γδ low” species)^3^,^4^. However, γδ T cells constitute more than 20% of the peripheral T cells in artiodactyls, rabbits and chickens (“γδ high” species)^5^,^6^,^7^,^8^. Recently, unconventional TCR chains that use Ig-like V domains have been discovered in a few distantly related vertebrate species. These unconventional TCR chains include TCRδ that uses VHδ and is found in amphibians, birds, and duckbill platypus^9^,^10^,^11^, the NAR-TCR found in cartilaginous fish^12^, and the TCRμ, which is only found in nonplacental mammals^13^,^14^.

As the representative of the anseriform birds, ducks split from the related chicken approximately 65–70 million years ago^15^. Moreover, the duck is not only one of the most economically important waterfowl, but is also a particularly good animal model for research in immunology because it serves as a natural reservoir of influenza A viruses and carries all 16 haemagglutinin and 9 neuraminidase subtypes^16^. Typically, ducks do not show apparent signs of disease upon infection with many strains of highly pathogenic H5N1, making them a Trojan horse for the maintenance of H5N1 in nature^17^,^18^. Recently, the molecular basis of the natural resistance of ducks to influenza infection has become a hot topic in avian immunology. Numerous innate immune-related genes, such as *RIG-I* (also called *DDX58*)^19^, *RNF135*^20^, and gene families of *IFITM, BTNL*, and *β*–*defensin*^21^,^22^, as well as the repertoire, expression and function of Ig isotypes^23^, have been extensively studied in ducks, but little is known about the duck TCR genes. In this study, we report the detailed genomic organization and repertoire diversity of all TCR loci in White Peking duck, including three conventional TCR loci (TCRα/δ, TCRβ and TCRγ) and the recently discovered TCRδ2 locus, providing a theoretical basis for further understanding of the avian adaptive immune system as well as the evolutionary relationships of TCRs in vertebrates.

## Results

### Genomic organization and germline repertoire of duck TCR genes

#### TCRα/δ locus

**Figure 1:**
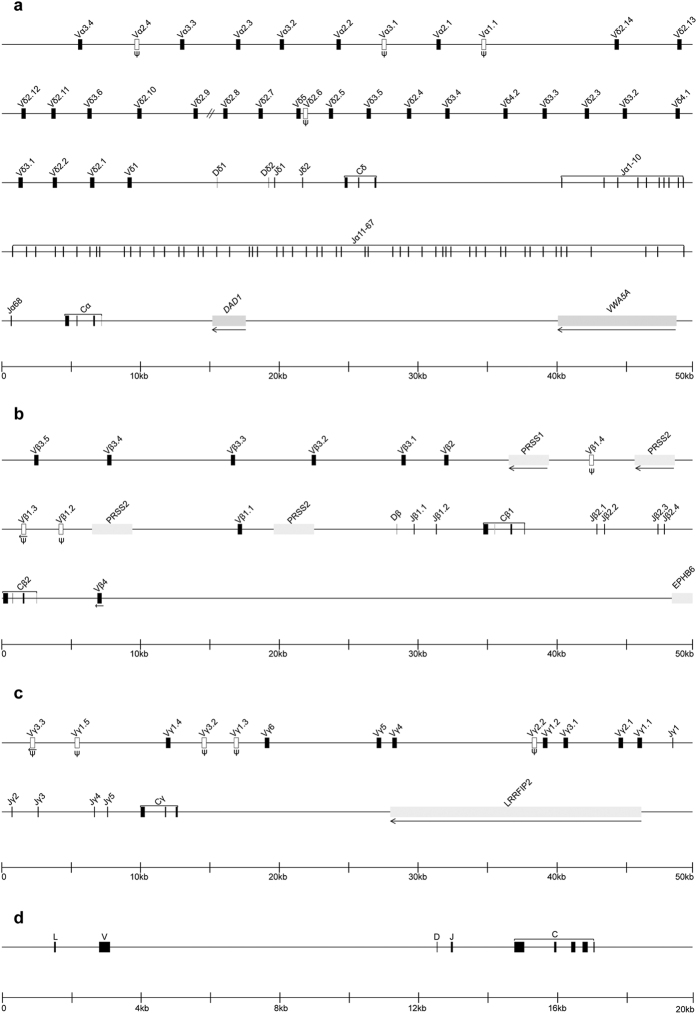
The genomic organization of duck TCR gene loci, TCRα/δ (**a**), TCRβ (**b**), TCRγ (**c**) and TCRδ2 (**d**). V, variable gene segment; D, diversity gene segment; J, joining gene segment; C, constant gene segment. V segments are named 3′ to 5′ with the subgroup number followed by the gene segment number if there were more than one member in the subgroup. Functional V segments are shaded, and pseudogenes are shown in hollow boxes and marked with a ψ. Non-TCR genes located in or flanking each TCR locus are shown in light grey. V segments and non-TCR genes with an opposite transcriptional orientation to the relevant C region are indicated by an arrow, and the sequence gap in (**a**) and (**c**) is marked as //.

Based on the mallard TCRα cDNA sequence (accession number AF323922), we first identified a Cα gene-positive BAC clone, DHS1503D01. According to the end sequence of DHS1503D01, another BAC clone, DHS1008P13, was found to overlap the 5′ portion of DHS1503D01 and contain the Cδ gene. An analysis of the two BAC sequences showed that the δ locus was located within the α locus, resembling the genomic organization of the TCRα/δ locus in other tetrapods (Fig. 1a).

**Figure 2:**
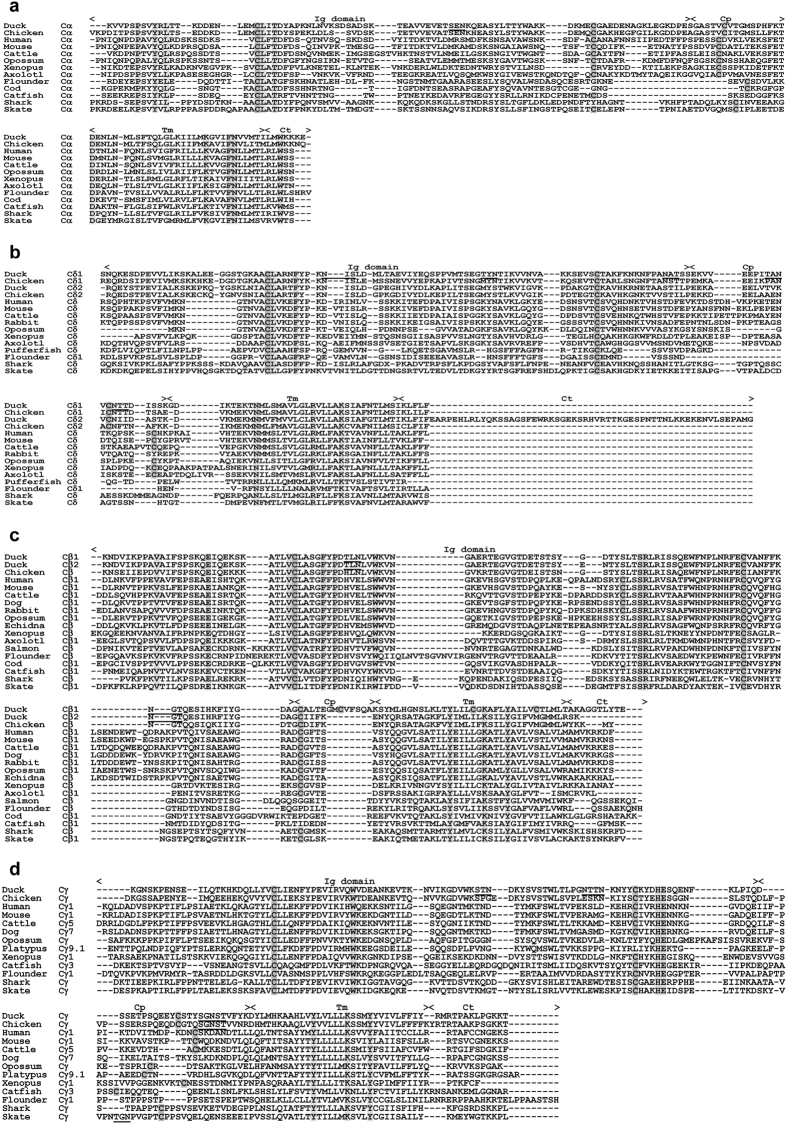
Alignment of deduced amino acid sequences of TCR C regions in selected vertebrates, Cα (**a**), Cδ (**b**), Cβ (**c**) and Cγ (**d**). The different domains of the C region are indicated above the sequences. Dashes indicate gaps. Canonical amino acids are shaded, and the potential N-linked glycosylation sites are underlined.

The duck Cα and Cδ genes were encoded by three exons that successively encoded the Ig domain, connecting peptide (Cp), and transmembrane-cytoplasmic (Tm-Ct) domain, all of which contained the three conserved cysteines required for intra- and inter-chain disulfide bond formation and the conserved lysine and arginine residues responsible for the interaction with other TCR dimers (Fig. 2a and b). A comparison of the amino acid sequences of duck Cα and Cδ with the corresponding sequences of other vertebrate species revealed maximum identity levels (71.6% for Cα and 66.2% for Cδ) between the duck and chicken, but less than 35% identity between the duck and other animal species. One and five potential N-glycosylation sites were identified in duck Cα and Cδ, respectively (Fig. 2a and b). At least 68 functional Jα segments were identified between the Cδ and Cα genes, and at least two Dδ and two functional Jδ segments were found upstream of the single Cδ gene (Supplementary Fig. S1A and B).

Within the BAC sequences, we further identified 33 V segments, all of which were located 5′ upstream of the first Dδ segment (Fig. 1a). When the nucleotide identity was compared with the V segments defined in other species, the V segments could be further categorized into one of two distinct groups, 9 Vα at the 5′ end and 24 Vδ located downstream of the Vα group. Of the 33 V segments, four were found to be pseudogenes due to non-sense mutations (Vα2.4 and Vα3.1), frameshift (Vα1.1) or the absence of the exon encoding the leader peptide (Vδ2.6). Using 5′ RACE, 40 extra Vα and 6 extra Vδ segments were detected in the cDNA clones, indicating that the current TCRα/δ locus is incomplete. Based on the criterion that V segments belonging to the same subgroup should share 75% or greater nucleotide identity^24^, a total of 49 (9 + 40) Vα segments could be grouped into three subgroups (Vα1 to Vα3) (Table 1) (Supplementary Fig. S2A), and the total of 30 (24 + 6) Vδ segments were categorized within five subgroups (Vδ1 to Vδ5) (Table 1). The Vδ2 appeared to be the largest Vδ subgroup, consisting of 20 Vδ segments (Supplementary Fig. S2B). Members within a Vα or Vδ subgroup exhibited more than 76.4% or 75.1% nucleotide identity. Within a subgroup, each Vα or Vδ segment cloned from 5′ RACE displayed 76.4% to 96.9% or 75.1% to 96.0% nucleotide identity with the remaining Vα or Vδ segments, respectively. The only exception is Vα3.16, which displayed 97.1% nucleotide identity with the pseudogene Vα3.1. Since the Vα3.16 is functional, it was considered as a novel Vα segment.

Dot plot analyses indicated that both Vα and Vδ regions had undergone multiple duplications (Supplementary Fig. S4A). The current incomplete Vα region originated from tandem duplications of a homology unit containing one Vα2 and one Vα3 segment (Supplementary Fig. S4B). The Vδ region contained several ~4 kb repeated units, which were composed of V segments from Vδ2 and Vδ3 subgroups (Supplementary Fig. S4C).

We also performed genomic Southern blotting using probes from C and selected V subgroups. The detection of only one band with the Cα probe verified that there was only a single copy of the Cα gene in the duck genome (Supplementary Fig. S3A). However, one dark and two light bands were detected when the enzyme *Pst* I and the Cδ probe were used, indicating that another Cδ-like gene might be located outside the TCRα/δ locus (Supplementary Fig. S3B), resembling the second TCRδ locus identified in chicken and zebra finch, as discussed later. The number and intensity of hybridizing bands substantiated the presence of larger number of Vα3 and Vδ2 segments in the genome. However, compared with the number of V segments obtained thus far, more bands were detected using the Vα1 and Vδ5 probes, suggesting the presence of additional germline members within the Vα1 and Vδ5 subgroups. (Supplementary Fig. S3A and B).

#### TCRβ locus

According to the mallard TCRβ cDNA sequence (accession number AY039002), a Cβ gene-positive BAC clone, DHS0801D24, was identified and sequenced. Analysis of the BAC sequence revealed that the duck TCRβ D, J, and C genes were organized in a unique pattern, Dβ-(Jβ)_2_-Cβ1-(Jβ)_4_-Cβ2 (Fig. 1b), in contrast to the tandem-aligned D-(J)_n_-C clusters in most mammals or the translocon organization with a greater number of Jβ genes in some teleosts.

Both Cβ1 and Cβ2 genes consisted of four exons. The first exon of the two Cβ genes, which encoded the Ig domain, was highly conserved with only three amino acid changes. However, the following exons were substantially divergent, with only 33% identity at the amino acid level. Maximal differences in length and nucleotide composition have been observed in exon 2, which was found to encode Cp. Exon 2 of Cβ1 encoded as many as 14 amino acids, whereas exon 2 of Cβ2 encoded only six amino acids. In Cβ2, both Tm and the cytoplasmic Ct domain were encoded by exon 3, and exon 4 contained only the 3′ untranslated region (3′ UTR). By contrast, exon 4 of Cβ1 encoded eight extra amino acids, forming a longer Ct domain. In addition to the canonical cysteine required for intra- and inter-chain disulfide bond formation, Cβ1 encoded three extra cysteine residues, one in the Cp domain and the other two in the Tm domain. The Ct domains of both Cβ genes contained a lysine residue that was involved in the interaction with the CD3 complex. Two potential N-glycosylation sites were identified in both Cβ1 and Cβ2 (Fig. 2c). Southern blotting analysis further corroborated the presence of two Cβ genes in the duck genome (Supplementary Fig. S3C). The single Dβ segment had a 13-bp G-rich coding region that could be productively read in all three frames (Supplementary Fig. S1C). All six Jβ segments were functional and shared less than 60% amino acid sequence homology (Supplementary Fig. S1C).

Upstream of the Dβ gene, we identified ten Vβ segments. Among them, three were pseudogenes due to in-frame stop codon. As in mammals, a single Vβ gene (Vβ4) with an inverted transcriptional orientation was located 3′ downstream of Cβ2 (Fig. 1b). The current TCRβ locus is also incomplete because two extra Vβ segments, designated as Vβ3.6 and Vβ3.7, were cloned from 5′ RACE PCR. The total 13 duck Vβ segments could be grouped into four subgroups (Vβ1 to Vβ4) (Table 1) (Supplementary Fig. S2C). Members within a Vβ subgroup shared more than 91.4% nucleotide identity. The two Vβ segments cloned from 5′ RACE exhibited 91.4% and 95.3% nucleotide identity with the remaining Vβ3 members, respectively. Dot-plot matrix showed two regions containing tandem duplications, one corresponding to Vβ3 subgroup and the other comprising of three copies of a homology unit, in which a *PRSS2* gene and a Vβ1 segment are located (Supplementary Fig. S4D). Southern blotting analysis substantiated the presence of larger number of Vβ3 segments and smaller number of Vβ2 segments in the genome (Supplementary Fig. S3C).

#### TCRγ locus

The BAC clone DHS0702G12 was isolated using primers designed to amplify the first exon of the mallard Cγ cDNA (accession number AF378702). BAC end sequencing demonstrated that this clone likely encompassed most of the duck TCRγ locus. Shotgun sequencing of this BAC clone provided three contigs, contig 14 (5,853 bp), contig 34 (5,335 bp), and contig 53 (183,893 bp), which were located sequentially 5′ to 3′ but did not overlap.

The duck TCRγ locus exhibited a translocon organization. A single Cγ gene containing three exons was identified in BAC clone DHS0702G12 and was also detected in the genomic Southern blotting assay (Fig. 1c and Supplementary Fig. S3D). Exon 1 encoded the extracellular Ig domain, which contained two conserved cysteine residues that were required for intra-chain disulfide bond formation and three N-glycosylation sites. Exon 2 encoded a short Cp containing the single conserved cysteine that formed the inter-chain disulfide bond with TCR Cδ, and exon 3 encoded the Tm, a positively charged Ct, and the 3′UTR regions. As expected, pairwise alignments showed that the duck TCR Cγ chain exhibits the highest amino acid identity (67.1%) with the chicken TCR Cγ chain, but low amino acid identity (less than 30%) with those of other vertebrates (Fig. 2d).

Thirteen Vγ segments were identified in BAC clone DHS0702G12. Of them, five were pseudogenes due to an in-frame stop codon (Fig. 1c). Two extra Vγ segments, designated as Vγ1.6 and Vγ3.4, respectively, were cloned by 5′ RACE PCR, suggesting that there are at least two germline Vγ segments located 5′ upstream of the Vγ3.3 in the genome. All 15 duck Vγ segments could be divided into six subgroups (Vγ1 to Vγ6) based on the same criterion applied for TCRα/δ and TCRβ (Table 1) (Supplementary Fig. S2D). Members within a Vγ subgroup shared more than 79.3% nucleotide identity. The Vγ1.6 or Vγ3.4 displayed 81.0% to 93.3% or 78.9% to 89.6% nucleotide identity with the remaining Vγ1 or Vγ3 segments, respectively. Dot plot analysis indicated no duplicated units longer than 2 kb in the current Vγ region (Supplementary Fig. S4E). The results of Southern blotting of the representative Vγ subgroups are shown in Supplementary Fig. S3D. The number and intensity of hybridizing bands substantiated the presence of larger number of Vγ1 segments and smaller number of Vγ5 segments in the genome. Between Vγ1.1 and Cγ, five functional Jγ segments were identified (Supplementary Fig. S1D).

#### TCRδ2 locus

Based on the previously reported cDNA sequence encoded by the duck TCRδ2 locus (accession number AF415216)^10^, we obtained the complete genomic sequence of this locus in a BAC clone, DHS0901N17. The duck TCRδ2 locus spanned approximately 16 kb and had a conserved organization similar to that of chicken, containing a single cluster of VHδ, Dδ, Jδ, and Cδ genes (Fig. 1d). The VHδ gene was flanked by typical 3′ 23-RSS, and the D gene had 5′ 12-RSS and 3′ 23-RSS, which were canonically used in the TCRδ. The Jδ gene had 5′ 12-RSS and a conserved splice site at the 3′ end. The Cδ2 gene consisted of five exons. Exon 1 encoded the extracellular Ig domain, exon 2 encoded a short Cp, and the last three exons together encoded the Tm and a long Ct containing 62 amino acids, as well as the 3′UTR regions (Fig. 2b). To determine whether duck has more than one TCRδ2 locus in its genome, Southern blotting was performed using one probe from VHδ and one probe from exon 1 of Cδ2. Because the enzyme sites of *Pvu* I and *Pst* I were located in the VHδ sequence, one dark and one light band were detected using the VHδ probe (Supplementary Fig. S3E). We also found one dark and one light band using the enzyme *Pst* I and the Cδ probe (Supplementary Fig. S3E). The single dark band corresponded to the actual Cδ2 gene, but the single light band seemed to be the conventional Cδ gene, in which exon 1 shares 59.5% nucleotide identity with that of the Cδ2 gene (Fig. 2b).

### Phylogenetic analyses of duck Vα, Vδ, Vβ, and Vγ gene segments

**Figure 3:**
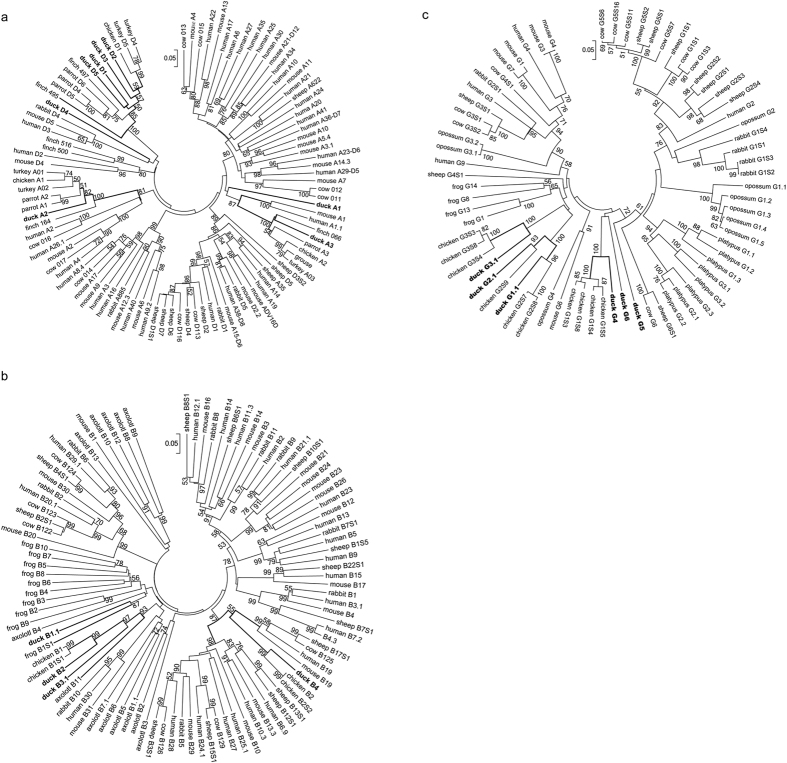
Phylogenetic analyses of the TCR V gene segments from representative mammalian, avian and amphibian species, Vα and Vδ (**a**), Vβ (**b**) and Vγ (**c**). The unrooted trees were constructed using the neighbour-joining method with nucleotide sequences corresponding to FR1 through FR3. Duck V gene segments are indicated in bold. Numbers next to the branches show the percentages of the nodes in 1,000 bootstrap replicates.

As shown in Fig. 3a, the duck Vα2 and Vα3 subgroups were closely related to the chicken (and zebra finch) Vα1 and Vα2 subgroups, respectively, and orthologous genes have also been found in mammals. The duck genes from the Vδ1, Vδ2, Vδ3, and Vδ5 subgroups fell in the same phylogenetic clade with the Vδ1 subgroup of chicken as well as the Vδ1 and Vα3 subgroups of zebra finch, but this clade was distinct and specific for birds. However, the duck Vα1 and Vδ4 subgroups did not clearly cluster with any Vα or Vδ genes from other birds or mammals (Fig. 3a).

Although duck Vβ genes belonging to subgroup Vβ2 and Vβ3 were classified as distinct subgroups, both subgroups fell in the same phylogenetic clade as the chicken Vβ1 subgroup, and all were derived from a common ancestral gene that was also present in amphibians. The duck Vβ1 subgroup lacked orthologues in chicken and mammals but demonstrated a clear relationship with amphibian Vβ genes. Conversely, the duck Vβ4 was closely related to the Vβ genes from chicken Vβ2 and many mammalian Vβ subgroups but lacked a known orthologue in amphibians, suggesting its emergence after the separation of amphibians (Fig. 3b).

In contrast to Vβ genes, all duck Vγ subgroups showed a high specificity to birds, except the Vγ6 subgroup, which formed a weakly supported group (72% support) with the clade containing all mammalian Vγ genes. The duck Vγ1 and Vγ2 subgroups clustered with chicken Vγ2, and the Vγ3 and Vγ4 subgroups clustered with chicken Vγ3 and Vγ1, respectively. The Vγ5 subgroup appeared to have evolved separately in duck or anseriform species because it did not clearly cluster with any Vγ genes from other tetrapods (Fig. 3c).

### Expression of duck TCR genes in various tissues

**Figure 4:**
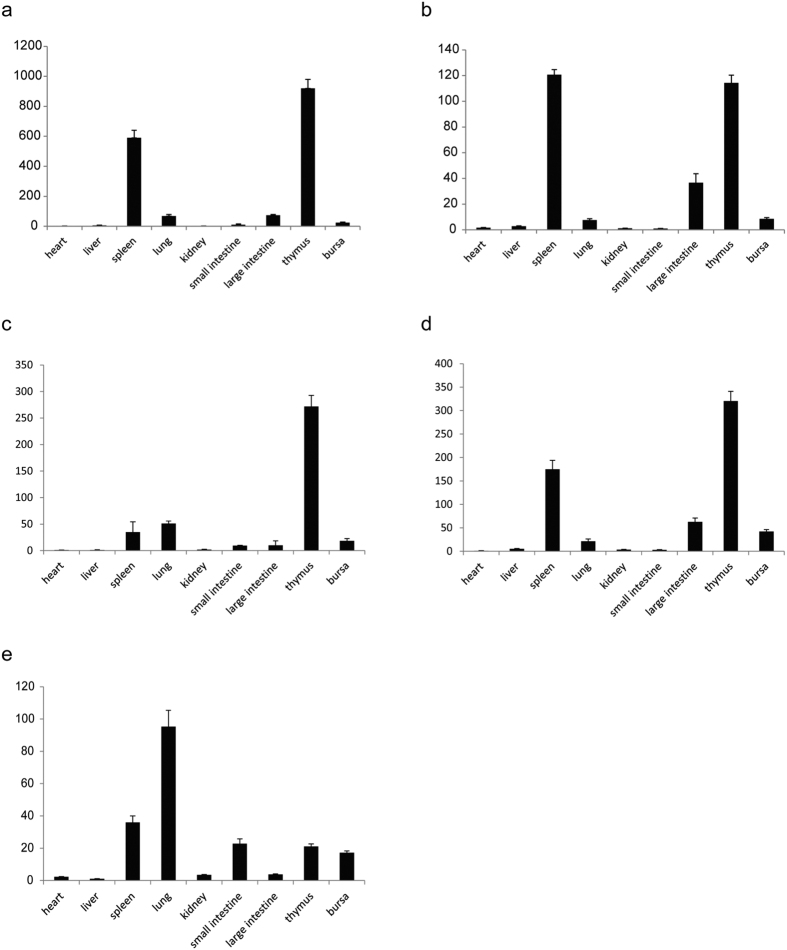
Quantitative real-time PCR analyses of the relative expression levels of duck TCR genes in different tissues, TCRα (**a**), TCRδ (**b**), TCRβ (**c**), TCRγ (**d**), and TCRδ2 (**e**). The duck *EF1A1* gene was chosen as an internal control. The *y*-axis shows the normalized fold changes in expression, and the nine tissues are listed below the *x*-axis.

The expression pattern of duck TCR genes in different tissues was assessed by quantitative real-time PCR. TCRα, γ, and δ1 were highly expressed in the thymus and spleen, and relatively weakly in the large intestine, lung, and bursa, but they were barely detectable in other tissues (Fig. 4a,b and d). TCRβ was only expressed at high levels in the thymus; it was expressed at much lower levels in other tissues, including the spleen (Fig. 4c). Unexpectedly, TCRδ2 was expressed at the highest levels in the lung but relatively weakly in lymphoid tissues, including the spleen, small intestine, thymus, and bursa (Fig. 4e), indicating that the TCRδ2 may play a crucial role in the tolerance of ducks to avian influenza viruses.

### Diversity of conventional TCR transcripts in duck

Based on the results of 5′ RACE PCR, a total of 142 TCRα, 76 δ, 42 β1, 43 β2, and 102 γ cDNA clones were sequenced, and after removing duplicates, 134 α, 75 δ, 42 β1, 43 β2, and 102 γ remaining clones were considered unique. These clones were analysed for the use of V, D, and J gene segments and overall CDR3 diversity.

#### TCRα

Of 134 unique TCRα cDNA clones, 112 clones were deemed potentially functional based on their complete ORFs. In general, members of subgroup Vα3 (75 clones) appeared to be more frequently utilized than those of subgroups Vα1 (29 clones) and Vα2 (30 clones). However, excluding Vα3.4, none of the germline Vα segments presented in the BAC sequence were found in the 5′ RACE clones (Supplementary Fig. S5A). For the potentially functional clones, the length of CDR3 was 28.4 ± 6.2 bp, encoding 4 to 16 amino acid residues with an average of 9.5 residues (Supplementary Figs S5A and S6A).

#### TCRδ

Among 75 unique TCRδ cDNA clones, 57 clones had an intact ORF. Forty-nine clones utilized 20 Vα segments, of which nine were also used by TCRα. Notably, none of the functional members belonging to subgroup Vα1 were used in the TCRδ rearrangement, and in contrast to TCRα, all of the germline Vα segments identified in the BAC sequence, excluding the pseudo Vα3.1, participated in TCRδ rearrangement, indicating that TCRα and TCRδ have different usage preferences for the Vα segments (Supplementary Fig. S5B). In the remaining 26 clones containing Vδ segments, members of the subgroup Vδ2 (18 clones) were more frequently used, whereas members of the subgroup Vδ4 were not observed (Supplementary Fig. S5B). There appeared to be a Jδ usage preference. The Jδ1 segment, which has a more conserved heptamer in its RSS, accounted for more than two-thirds (57 clones) of the expressed Jδ repertoire (Supplementary Fig. S5B). Most VJ junctions contained either one (10 clones for Dδ1 and 27 clones for Dδ2) or both (31 clones) Dδ segments. Among them, N and P nucleotide additions between different gene segments were common. However, the remaining seven clones demonstrated evidence for N nucleotide additions but no D segment incorporation, indicating extensive trimming of D or direct Vα/δ-Jδ recombination during rearrangement. For the potentially functional clones, the length of CDR3 was 34.2 ± 9.0 bp and encoded 5 to 19 amino acid residues, with an average of 11.5 residues (Supplementary Figs S5B and S6B).

#### TCRβ

Of the 42 unique TCRβ1 and 43 β2 cDNA clones, 74 clones were considered to be potentially functional. Both TCRβ1 and β2 showed a similar usage pattern of Vβ segments. A total of 55 clones (24 of β1 and 31 of β2) used the V segments from subgroup Vβ3, which contained the most germline members. Notably, the most frequently used V segment was Vβ2, the single member of subgroup 2, accounting for more than 20% of the expressed Vβ repertoire in both β1 (12 clones) and β2 (9 clones), whereas Vβ1.1, the only functional segment from subgroup 1, was not observed in the cDNA of either β1 or β2 (Supplementary Fig. S5C). Similar to TCRδ, TCRβ also demonstrated a biased usage of Jβ segments, especially β1, which utilized Jβ1.2 more frequently than Jβ1.1 (37 vs. 6 clones) (Supplementary Fig. S5C). Due to the single Dβ segment, the CDR3 length of TCRβ was 30.9 ± 7.1 bp, encoding 5 to 17 amino acid residues (average of 10.2 residues) (Supplementary Figs S5C and S6C). The features described above for TCRδ junctions were also found in the TCRβ junctions (Supplementary Fig. S5C).

#### TCRγ

Among 102 unique TCRγ cDNA sequences, 93 clones displayed an intact ORF. All potentially functional Vγ segments identified in the BAC sequence, excluding Vγ5, were found in the cDNA clones. Members of subgroup 1 (49 clones) and 3 (37 clones) were preferentially used, especially Vγ1.6 and Vγ3.4, which were not located on the BAC sequence but each contributed to approximately 20% (20 clones) of the expressed Vγ repertoire (Supplementary Fig. S5D). For all potentially functional clones, the average length of CDR3 was 24.1 ± 8.4 bp, encoding 2 to 16 amino acid residues with an average of 8 residues (Supplementary Figs S5D and S6D).

### Diversity of duck TCRδ2 transcripts

The total RNA of thymus tissue as well as the primers complementary to VHδ and Cδ2 (Supplementary Table S1) were used in RT-PCR to investigate the junctional diversity of the duck TCRδ2 transcripts. A total of 18 TCRδ2 cDNA clones were sequenced, and after removing the duplicates, the remaining 16 clones were considered unique. The junctional diversity of the duck TCRδ2 repertoire was characterized by clear P nucleotide additions to the 3′ ends of both V and D regions in almost all TCRδ2 clones. For 13 productive rearranged clones, the average length of CDR3 was 36.6 ± 6.1 bp, encoding 9 to 14 amino acid residues with an average of 11.5 residues (Supplementary Figs S5E and S6E).

## Discussion

Compared with TCRα/δ and TCRγ gene loci, the germline repertoire of the TCRβ locus has been extensively studied in many vertebrates. Among all mammals studied to date, the genomic organization of the TCRβ locus is highly conserved, with a pool of Vβ genes positioned at the 5′ end and several tandem repeated Dβ-(Jβ)_4~7_-Cβ clusters followed by a single V gene with an inverted transcriptional orientation located at the 3′ end^25^,^26^,^27^,^28^,^29^,^30^,^31^. Cβ genes within each mammalian species maintain a high degree of sequence similarity in the coding region but present high divergence in the 3′UTR, indicating that the Cβ genes have undergone concerted evolution by intra-species homogenization using gene conversion^28^,^30^,^32^,^33^. However, the genomic organization of the TCRβ locus and concerted evolution of the Cβ genes that seems to be conserved in mammals are not present in other vertebrate species, especially in teleosts. The TCRβ locus of zebrafish resembles that observed in mammals, but the D gene is absent from the second Dβ-(Jβ)_n_-Cβ cluster^34^. The TCRβ locus of channel catfish (*Ictalurus punctatus*) is arranged in a typical translocon organization containing a single Dβ gene followed by a total of 29 Jβ genes and two tandem Cβ genes^35^. Notably, the sequence similarity of Cβ isotypes within a single teleost species varies considerably. In the Japanese flounder (*Paralichthys olivaceus*) and Atlantic cod (*Gadus morhua*), different Cβ isotypes show more than 85% amino acid identity^36^,^37^. Conversely, in both zebrafish and catfish, the sequences of two Cβ isotypes are substantially different, sharing only 36% identity at the amino acid level^34^,^35^. Such multiple divergent Cβ isotypes have also been observed in bicolour damselfish (*Stegastes partitus*)^38^, as well as an urodele amphibian Mexican axolotl (*Ambystoma mexicanum*)^39^. Before this study, chicken was the only other bird for which the sequences of the TCRβ D-J-C region had been reported. The locus contains a single Dβ, 4 Jβ genes and a seemingly single Cβ gene^40^. In this study, we determined the complete sequence of the duck TCRβ locus, which is arranged in an unusual fashion, similar to that of the zebrafish, with a single Dβ gene followed by two tandem-aligned (Jβ)_n_−Cβ clusters. The absence of the 2nd Dβ gene in ducks may have occurred as an independent event and happens to form a functional genotype that is similar to that of zebrafish. Another attractive feature of duck TCRβ lies in the sequence conservation of each domain between the two Cβ genes. The Ig domains of the two Cβ are well-conserved, whereas the following Cp, Tm, and Ct domains differ remarkably. This special distribution of Cβ identity has not been reported in any other vertebrates, in which the sequence identity is high (>80%) or low (<50%) throughout the whole coding region of the different Cβ genes. Furthermore, the coding sequence of the Ct domain is entirely included within exon 3 of Cβ2 but separated into two exons by intron 3 in Cβ1, suggesting that the two Cβ genes might be the result of an ancient duplication that occurred long before the speciation of *Anas*.

The birth/death hypothesis has been postulated as an evolutionary mechanism of V genes from both Ig and TCR loci^41^. Recently, a phylogenetic analysis of genomic V-gene repertoires, which were extracted from mammals and reptiles with available WGS sequences, indicated that V genes from Ig and TCR loci might have markedly different evolutionary pathways. The Ig V genes undergo more pronounced birth/death processes, thereby permitting the frequent duplication of specific V subgroups that could directly recognize rapidly changing antigens in the external environment. By contrast, the V genes from the TCRα and TCRβ loci, which consist of multiple subgroups (Table 2) with relatively low duplication permissiveness throughout evolution, appeared to have undergone a co-evolution process with MHC molecules, resulting in natural evolutionary pressures^42^,^43^,^44^. As shown in Table 2, the most striking feature of duck Vα and Vβ genes is the presence of fewer subgroups in comparison to mammals. The same feature are also observed in the Vα and Vβ genes of chicken and zebra finch^10^,^45^,^46^. According to the co-evolution hypothesis, there might be some evolutionary connections between the diversity of Vα/Vβ subgroups and the number of expressed classical MHC loci. A larger number of expressed MHC genes would result in the positive selection of a more diverse TCR repertoire, but too many expressed MHC class I genes would also reduce the T cell repertoire during negative selection. Currently, the precise numbers of MHC class I and/or MHC class II genes have been ascertained in only a few birds. The chicken *MHC-B* locus contains two classical MHC class I genes (*BF1* and *BF2*) and two classical MHC class II B genes (*BLB1* and *BLB2*). However, only *BF2* and *BLB2* are dominantly expressed at the RNA and protein levels^47^. Similarly, among the five MHC class I genes in duck, only *UAA* is a dominantly expressed classical MHC class I gene; the others are the weakly expressed *UDA* and unexpressed pseudogenes (*UBA, UCA*, and *UEA*)^48^. Furthermore, in the genome sequence of zebra finch, only one functional MHC class I gene has been identified^49^. The above examples suggest that the evolution of fewer Vα/Vβ subgroups is probably due to the dominant expression of a single classical MHC class I gene in these avian species, providing an opportunity for the co-evolution of both MHC and TCR genes with associated roles in presenting and recognizing antigens.

As summarized in Table 2, many more functional germline Vδ genes have been identified in “γδ high” species than “γδ low” species, indicating that the germline diversity of the Vδ gene is directly proportional to the percentage of peripheral γδ T cells in mammals and chicken. Furthermore, three important points are relevant to the Vδ genes. First, the subgroup numbers of Vδ genes show no significant differences between the “γδ high” and “γδ low” species. Second, an enormous expansion of the germline repertoire of some Vδ subgroups is a striking feature observed in “γδ high” species. For example, the Vδ1 subgroup of cattle, sheep, and pig contains at least 52^50^, 40^51^, and 31^52^ members, respectively. Finally, the single Vδ subgroup of chicken, which contains as many as 36 members^45^, falls into a bird-specific clade without any mammalian counterparts in the phylogenetic analysis. Taken together, these findings suggest that the Vδ genes evolved following birth/death pathways similar to those that gave rise to Ig because antigen recognition by both Ig and the γδ TCR complex is not MHC-restricted^3^. Although the distribution of T-cell populations in birds except the chicken remains to be determined, the presence of such a large number of Vδ genes as well as the expansion of the Vδ2 subgroup suggest that the duck probably belongs to the “γδ high” species.

The length distribution of the CDR3 loop has been used as a metric in assessments of the possible range of binding paratopes generated by a given TCR type and has been analysed in human, mouse^53^, Japanese flounder^36^, and nurse shark^54^, albeit the data of the latter two species were reported from a limited sample size (Table 3). In the human, mouse, and nurse shark, the CDR3δ loops display a much broader length distribution than in the other three TCR types because of the presence of multiple D gene segments (for the mouse and human) that can join together, as well as the numerous N nucleotides (for the nurse shark) inserted into the V-D and D-J junctions. Notably, although 0 to 2 putative Dδ segments were shown to incorporate into a single CDR3δ, the duck CDR3δ loop lengths are 5–19 amino acid residues, similar to the ranges for the other TCR types of duck. However, the CDR3γ loops in the human, mouse, Japanese flounder, and nurse shark display a narrow length distribution (1–12, 4–11, 5–10, and 6–12, respectively), whereas the duck CDR3γ loops exhibit a broader distribution with 2–16 amino acid residues, which is far beyond the range exhibited by the listed counterparts. Given that the γδTCR can interact with diverse ligands in various ways, it is likely that the broad length distribution of CDR3γ compensates for the narrow length distribution of CDR3δ in ducks. The CDR3α and CDR3β loops of human, mouse, and Japanese flounder have, on average, very similar lengths (9.2 vs. 9.5, 8.5 vs. 8.9, and 11.2 vs. 11.2, respectively). The average lengths of the duck CDR3α and CDR3β loops show a tendency similar to the three species, although the average CDR3α loops appear to be 0.7 amino acid residues shorter than CDR3β (9.5 vs. 10.2). However, the duck CDR3α and CDR3β loops, ranging from 4–16 and 5–17 amino acid residues, display much wider distribution than those of the three species (6–12, 6–12 and 7–15 for CDR3β, as well as 6–12, 4–13, and 7–15 for CDR3β). This indicates that duck CDR3α and CDR3β may have increased flexibility and are therefore better suited to recognize a larger number of antigenic conformations presented by MHC molecules.

## Methods

### Animal, DNA and RNA isolation and reverse transcription

A White Peking Duck aged 90 days post-hatching was purchased from Beijing Jinxin Duck Centre. Genomic DNA was extracted from blood cells following a routine phenol-chloroform protocol. Total RNA was isolated from various tissues using an RNeasy Mini Kit (Qiagen, Valencia, CA, USA). Reverse transcription was conducted using M-MLV reverse transcriptase (Invitrogen, Beijing, China) with an oligo(dT) adapter primer NotI-d(T)18 (Supplementary Table S1). Animal care was in accordance with the guidelines of China Agricultural University for animal welfare. All animal experiments in the present study were approved by the Animal Care and Use Committee of China Agricultural University.

### Bacterial artificial chromosome (BAC) genomic library

The White Peking Duck BAC (bacterial artificial chromosome) genomic library was constructed by Majorbio Co. Ltd., Shanghai, China. The BAC library was divided into two sub-libraries, each of which was prepared using blood cell genomic DNA that had been partially digested with the restriction enzymes *Hind* III or *Bam* HI. Each sub-library was composed of 49,152 clones, which were placed into 16 superpools of 8 × 384-well plates. Using pulsed-field gel electrophoresis analysis of 185 clones that were randomly selected from two sub-libraries, the average insert sizes were estimated to be 152 kb.

### BAC screening and sequencing

Positive BAC clones covering the duck TCRα/δ, β, γ and δ2 loci were isolated from the BAC library via PCR-based screening with primers (Supplementary Table S1) designed based on the available TCR mRNA constant sequences of mallard from GenBank. For TCRα/δ, the first positive BAC clone was sequenced from both ends, and the end sequences were used to design primers (Supplementary Table S1) for the next round of screening to determine the BAC clone overlap. The positive BAC clones were subjected to shotgun sequencing and assembled using the next-generation sequencing platform by BGI (Beijing, China).

### Identification of germline V, D, J and C gene segments

To determine the locations of the V gene segments, BAC sequences were screened using the IgBLAST algorithm (http://www.ncbi.nlm.nih.gov/igblast/) by similarity to homologues from human and mouse. V gene segments are named 3′ to 5′ with the subgroup number followed by the gene segment number if there was more than one member in this subgroup. The D and J gene segments were annotated by searching the recombination signal sequences (RSS) using FUZZNUC (http://embossgui.sourceforge.net/demo/fuzznuc.html) and the conserved motif FGXG encoded by the J segments manually. The exon-intron organization of the C regions was searched manually by comparing the cDNA sequence encoding the complete C region for each TCR with the duck genomic sequences. Non-TCR genes located in or flanking each TCR locus were identified using GENSCAN (http://genes.mit,edu/GENSCAN.html).

### 5′ RACE

The 5′ RACE System for Rapid Amplification of cDNA Ends (version 2.0, Life Technologies/Gibco BRL, Gaithersburg, MD, USA) was applied to thymus total RNA to obtain the expressed repertoire of each TCR type as well as the novel expressed V segments that were not located on the BAC clones. Specific primers for each constant region of the TCRα/δ, β and γ loci are listed in Supplementary Table S1. The resulting PCR products were cloned into the pMD-19T vector (TaKaRa, Dalian, China) and sequenced.

### 3′ RACE

The cDNA sequences encoding the complete C region of each TCR, including the immunoglobulin domain, Cp, Tm, Ct and 3′UTR, were obtained by nested 3′ RACE PCR using thymus cDNA. Specific primers for each TCR gene were derived from the V region sequences. For the first round of PCR, sense primer located closer to the 5′ end of the cDNA (Supplementary Table S1) were paired with the antisense primer RT-P1. For the second round of PCR, a nested primer located 3′ to the original primer (Supplementary Table S1) was paired with antisense primer RT-P2, and a dilution of the first PCR was used as the template. The resultant PCR products were cloned into the pMD-19T vector and sequenced.

### Southern blotting

Genomic DNA was digested with different restriction enzymes and loaded into a 0.9% agarose gel, electrophoresed for 6 h, and transferred to a positively charged nylon membrane (Roche, Germany) for hybridization. The conserved Cα, Vα1, Vα2, Cδ, Vδ2, Vδ5, Cβ, Vβ2, Vβ3, Cγ, Vγ1, Vγ6, VHδ and Cδ2 sequences from White Peking duck were used as probes. These cDNA fragments were labelled using a PCR DIG Probe Synthesis Kit (Roche, Beijing, China) using the primers listed in Supplementary Table S1. Hybridization and detection were performed using the DIG High Prime DNA Labeling and Detection Starter Kit II (Roche, Beijing, China) according to the manufacturer.

### Detection of gene expression in different tissues by quantitative real-time PCR

The cDNA samples from nine tissues (heart, liver, spleen, lung, kidney, small intestine, large intestine, thymus and bursa) were used to determine the expression of TCRα/δ, β, γ and δ2 by quantitative real-time PCR. PCR was performed using a LightCycler 480 and LightCycler 480 SYBR Green I Master Mix (Roche, Beijing, China). Each sample was run in triplicate. The White Peking Duck *EF1a1* gene was used as the internal control. The PCR consisted of the following conditions: 95 °C for 10 min; 35 cycles of 95 °C for 10 s, 60 °C for 20 s, and 72 °C for 15 s; and a final extension at 72 °C for 7 min. The PCR primers are listed in Supplementary Table S1. The relative expression levels of the gene were determined using the 2^−ΔΔCt^ method by comparing the values with the internal control.

### Sequence analyses

DNA and protein sequence editing, alignments, and comparisons were performed using the DNASTAR Lasergene software suite^55^ and Boxshade software (http://www.ch.embnet.org/software/BOX_form.html). Dot plot analyses of the V regions of TCRα/δ, TCRβ and TCRγ loci were conducted with the dotter program^56^. For a given TCR type, if the V region (corresponding to FR1 through FR3) of a cDNA clone shared less than 97% nucleotide identity with the germline V segments identified in the BAC as well as V regions of every other cDNA clone, the V region was considered a novel V segment^57^,^58^. The CDR3 of the rearranged TCR V domain was defined as the region between the J region–encoded FGXG motif and the nearest preceding V region–encoded cysteine, according to the IMGT unique numbering system^59^. The length of CDR3 was defined as four amino acids less than the number of amino acid residues between the J region–encoded GXG triplet, where G is glycine and X is any amino acid, and the nearest preceding V region–encoded cysteine as described in ref. 53.

### Phylogenetic analyses

The nucleotide sequences corresponding to FR1 through FR3 of all V genes were aligned for tree construction using ClustalW. Phylogenetic trees were constructed in MEGA version 5.10^60^ using the neighbour-joining method with 1,000 bootstrap replicates. The GenBank accession numbers of all sequences used are listed in Supplementary Table S2.

## Additional Information

**How to cite this article**: Yang, Z. *et al*. A comprehensive analysis of the germline and expressed TCR repertoire in White Peking duck. *Sci. Rep.* **7**, 41426; doi: 10.1038/srep41426 (2017).

**Publisher's note:** Springer Nature remains neutral with regard to jurisdictional claims in published maps and institutional affiliations.

## Supplementary Material

Supplementary Information

## Figures and Tables

**Table 1:** Number of V gene segments found in the genome and cDNA analyses.

	Subgroup	Genome	cDNA
Vα	Vα1	1 (0)^a^	4 (4)
	Vα2	4 (3)	16 (15)
	Vα3	4 (3)	20 (19)
Vδ	Vδ1	1 (1)	—
	Vδ2	14 (13)	6 (6)
	Vδ3	6 (6)	—
	Vδ4	2 (2)	—
	Vδ5	1 (1)	—
Vβ	Vβ1	4 (1)	—
	Vβ2	1 (1)	—
	Vβ3	5 (5)	2 (2)
	Vβ4	1 (1)	
Vγ	Vγ1	5 (3)	1 (1)
	Vγ2	2 (1)	
	Vγ3	3 (1)	1 (1)
	Vγ4	1 (1)	—
	Vγ5	1 (1)	—
	Vγ6	1 (1)	—

^a^The numbers of functional genes are indicated in parenthesis.

**Table 2:** Numbers of TCR V segments and subgroups in selected mammals and birds.

Species	Vα^a^	Vβ	Vγ	Vδ	Reference
Human	54 (44–47), 41 (33–35)^b^	64–67 (40–48), 30 (21–23)	12–15 (4–6), 6 (2)	3 (3), 3 (3)	61
Mouse	98 (73–84), 23 (19)	35 (21–22), 31 (19)	7 (7), 5 (5)	6 (5), 5 (4)	61
Cattle	>300 (?),>38 (>33)	>134 (>79), 24 (19)	17 (17), 10 (10)	>100 (?), 6 (6)	28,62,63
Rabbit	—	75 (59), 24 (20)	10 (10), 4 (4)	—	30,64
Dog	—	37 (20), 25 (16)	16 (8), 8 (4)	—	29,65
Opossum	68 (56), 41 (33)	36 (27), 28 (21)	9 (9), 4 (4)	6 (4), 6 (4)	31
Platypus	89 (83), 17 (16)	—	>15 (?)^c^, 3^d^	10 (10), 2 (2)	11,66
Chicken	60 (50), 2 (2)	>10 (?)^c^, 2^d^	>24 (?)^c^, 3	36 (32), 1 (1)	45,67, 68, 69
Zebra finch	>10 (>10), 3 (3)	—	—	4 (4), 4 (4)	10
Duck	~49 (~44), 3 (3)	~13 (~11), 4 (4)	~15 (~10), 6 (6)	~30 (~29), 5 (5)	—

^a^The Vα segments include the Vα expressed in either TCRα and/or TCRδ chains.

^b^Numbers preceding the comma are the V segments, and numbers following the comma are the V subgroups. The numbers of functional segments or subgroups are shown in brackets.

^c^The numbers of V segments were deduced based on the numbers of hybridizing bands with probes of specific subgroups using genomic Southern blotting.

^d^The numbers of subgroups were deduced using cDNA sequences. “—” Indicates that no relevant information was available.

**Table 3:** CDR3 length of TCR chains in selected vertebrates.

Species	TCRα	TCR β	TCR γ	TCR δ	Reference
Duck	4, 16 (9.5)^a^	5, 17 (10.2)	2, 16 (8.0)	5, 19 (11.5)	—
Human	6, 12 (9.2)	6, 12 (9.5)	1, 12 (7.2)	8, 21 (14.5)	53
Mouse	6, 12 (8.5)	4, 13 (8.9)	4, 11 (8.8)	6, 19 (12.7)	53
Japanese flounder	7, 15 (11.2)	7, 15 (11.2)	5, 10 (8.5)	9, 17 (13.3)	36
Nurse shark	3, 12 (8.0)	0, 19 (9.6)	6, 12 (9.1)	4, 27 (9.8)	54

The mean length is bracketed. The CDR3 length was defined as four amino acids less than the number of amino acid residues between the J region–encoded GXG triplet, where G is glycine and X is any amino acid, and the nearest preceding V region–encoded cysteine.

^a^Range of CDR3 length: minimum, maximum.
