# Supplementary material for: A comprehensive analysis of the germline and expressed TCR repertoire in White Peking duck
Source: Sci Rep. 2017 Jan 30;7:41426. doi: 10.1038/srep41426 (PMC5278385; doi:10.1038/srep41426)
Supplement: Supplementary Information [file srep41426-s1.pdf]

A comprehensive analysis of the germline and expressed TCR repertoire in White Peking duck

Zhi Yang[1,3,+], Yi Sun[2,+], Yonghe Ma[1,+], Zhenrong Li[1], Yu Zhao[4], Liming Ren[1], Haitang Han[1], Yunliang Jiang[2,*], Yaofeng Zhao[1,*]

1 State Key Laboratory of Agrobiotechnology, College of Biological Sciences, China Agricultural University, Beijing, 100193, China

2 Shandong Provincial Key Laboratory of Animal Biotechnology and Disease Control and Prevention, College of Animal Science and Veterinary Medicine, Shandong Agricultural University, Taian, 271018, China

3 Beijing Advanced Innovation Center for Food Nutrition and Human Health, College of Food Science and Nutritional Engineering, China Agricultural University, Beijing, 100193, China

4 National Laboratory of Biomacromolecules, Institute of Biophysics, Chinese Academy of Sciences, Beijing, 100101, China

[*] yaofengzhao@cau.edu.cn; yljiang723@aliyun.com.

[+] These authors contributed equally to this work.

A

nonamer heptamer

Jα1: **atctttggt**aaagctctttgt**cacagtg**TAACTCCATACGTGCCTATGAAACACTCAGTTTTGGAGAGGGGACAAGGCTGACTGTTCTTCCAA
N S I R A Y E T L S F G E G T R L T V L P

Jα2: **agtttatgt**aaagcaccttat**cacagtg**TACTTGGTACTTATGGAAAGTTCACTTTTGGCGGAGGAACTCGCATGTCTGTGCTTCCAA
L G T Y G K F T F G G G T R M S V L P

Jα3: **cgtttttgt**tagtggacgtgt**cactgtg**TGACTTCAGTATCCAACTGGAAAGTCACCTTTGGAAGTGGAACACAGCTGGTAGTGAAACCTG
T S V S N W K V T F G S G T Q L V V K P

Jα4: **gatttttgt**aatgggaggaaa**tgctgtg**TGAATCTGGCTTCAGTGACAAGCTGACCTTTGGCTCAGGAACCAGGCTGTCCGTCCAACCAA
E S G F S D K L T F G S G T R L S V Q P

Jα5: **gcttttgt**aatgggaggaag**tgctgtg**TGACTATGGAACCAACAGGCTGACATTTGGCTCAGGGACCAGACTGTCCGTCCAACCAA
D Y G T N R L T F G S G T R L S V Q P

Jα6: **catttttgt**aatgagatgtgc**cacagtg**CGTACACTGAGGGCAAAGTCACTTTTGGAAAAGGAACTGTGCTTTCTGTAGTTCCAG
Y T E G K V T F G K G T V L S V V P

Jα7: **gctttttgt**aggggggaggaaa**tgctgtg**TGACTACAGCGGCGACAAGCTGACCTTTGGCTCAGGGACCAGGCTGTCTGTCCTACCAA
D Y S G D K L T F G S G T R L S V L P

Jα8: **catttgtgt**aatggcctttgg**tgctgtg**GGTGCTGAATGGAACAGGCTCACCTTTGGAGCTGGATCAAAGCTTTGTGTAAAGCCTC
G A E W N R L T F G A G S K L C V K P

Jα9: **gctttttgt**actgggaggaaa**tgctgtg**TGAATGACTTAAACAAGCTGACTTTTGGCTCAGGGACCAGGCTGTCCATCCAACCAA
N D L N K L T F G S G T R L S I Q P

Jα10: **catttattt**aatggacattgt**tgctgtg**AGTGCTGTCTTGAACAGGCTGAACTTTGGAGCAGGAACAAAGCTTTGTGTAATACCAG
S A V L N R L N F G A G T K L C V I P

Jα11: **ggtttttgt**aatggaaggaaa**tgctgtg**TGGCTCTAGCTCTTCATTCGGCAAATTGACCTTTGGCTCGGGGACCAGGCTGTCTGTCCAACCAA
G S S S S F G K L T F G S G T R L S V Q P

Jα12: **ggttcttgt**tgtggcaaaaag**cactgtg**TGAACTACGGCAAGATCACCCTTGGGAGCGGAACACGACTTCAGGTTTTGCCTA
N Y G K I T L G S G T R L Q V L P

Jα13: **gctttttgt**aatgggaggaaa**tgctgtg**TGACTATGACTACACCAACAAACTGACCTTTGGCTCAGGGACCAGGCTGTCCGTCCTACCAA
D Y D Y T N K L T F G S G T R L S V L P

Jα14: **catttttgt**aatgagacgtgc**cacagtg**AGTTAAATGCGCGCACAGTCACTTTTGGGAAAGGAACTGTGCTTTCAGTTGTTCCAG
L N A R T V T F G K G T V L S V V P

Jα15: **gctttttgt**aataggaggaaa**tgctgtg**TGGCACTGGCTCTACATACGGCAAATTGACCTTTGGCTCAGGGACCAGGCTGTCTGTCCAACCAA
G T G S T Y G K L T F G S G T R L S V Q P

Jα16: **agttcttgt**tgaggcaaaaag**cactgtg**TGAACTACGACAAGTTCACATTTGGGAAGGGAACACGACTTCAGGTTTTGCCTA
N Y D K F T F G K G T R L Q V L P

Jα17: **actttttgt**aatgggagtaca**tgctgtg**TGAGTGGCAACTACAAGCTGACCTTTGGCTCAGGAACCAGGCTGTCTGTCCTACCAA
S G N Y K L T F G S G T R L S V L P

Jα18: **gctttttgt**aatgggaggaaa**tgctgtg**TGACACTGGCTACAACAAGCCTACATTTGGCTCAGGGACTAGGCTGTCTGTCCTACCAA
D T G Y N K P T F G S G T R L S V L P

Jα19: **gctttttgt**agtgggaggaaa**tgctgtg**TGATTTTGGATCCTTCAACAAGCTGATCTTTGGCTCAGGAACCAGGCTGTCCGTCCTACCCA
D F G S F N K L I F G S G T R L S V L P

Jα20: **gctttttgt**aatgggaggaaa**tactgtg**TGGCTATGGCTCTGCATACGGCAGACCAACCTTTGGCACAGGAACCAGGCTTTCTGTCCAACCAA
G Y G S A Y G R P T F G T G T R L S V Q P

Jα21: **gctttttgt**aatgggaggaaa**tgctgtg**TGACTCTGGCTATGGCAACAAGCTGACCTTTGGCTCAGGAACCAGGCTGTCCGTCCTACCAA
D S G Y G N K L T F G S G T R L S V L P

Jα22: **ggtttttgc**tagtggatgtgt**cactgtg**TGACTTCAACGTCCACCTGGAAAGCCACCTTTGGAAGTGGGACACAGCTGGTGGTGAAACCTG
T S T S T W K A T F G S G T Q L V V K P

Jα23: **gctttttgt**aatgggaggaaa**tgctgtg**TGAATCTGGCTTCACCAAACTGACCTTTGGCTCAGGGACCCGGCTGTCCGTCCTACCAA
E S G F T K L T F G S G T R L S V L P

Jα24: **gctttttgt**tgtgggaagaaa**tgctgtg**TCACTACAGCAGTGACAAGCTGACCTTTGGCTCAGGGACCAGGCTGTCCGTCCTACCAA

H Y S S D K L T F G S G T R L S V L P

Jα25: **gcttttgt**aggggaggaaa**tattgtg**TGACTACAGCGGTGACAAGCTGACCTTTGGCTCAGGGACCAGGCTGTCTGTCCTACCAA
D Y S G D K L T F G S G T R L S V L P

Jα26: **catttgtgt**aatggcctttgg**tgctgtg**GGTGCTGACTGGAACAGGCTCACCTTTGGAGCTGGAACAAAGCTTTGTGTAAAGCCTC
G A D W N R L T F G A G T K L C V K P

Jα27: **cattttgt**aatgagacgtgc**cattgtg**TGTCCAATGCGTATGCAGTCACTTTTGGAAAAGGAACTGTGCTTTCAGTTGTACCAG
S N A Y A V T F G K G T V L S V V P

Jα28: **gcttttgt**aatgggaggaaa**tgctgtg**TGGCTATGGCTCTGGATATGACAGAATGACTTTTGGCTCAGGAACCAGGCTGTCTGTCCAACCAA
G Y G S G Y D R M T F G S G T R L S V Q P

Jα29: **agttcttgt**tgaggcaaaaag**cactgtg**TGAACTACGAGAAGCTCACATTTGGGAAGGGAACACGACTTCAGGTTTTGCCTA
N Y E K L T F G K G T R L Q V L P

Jα30: **acttttgt**aatgggagtaaa**tgctgtg**TGAGTGGCAACTACAAGCTGACCTTTGGCTCAGGAACCAGGCTGTCTGTCCTACCAA
S G N Y K L T F G S G T R L S V L P

Jα31: **gcttttgt**aatgggaggaaa**tgctgtg**TGACACTGGCTACAACAAGCCTACATTTGGCTCAGGGACTAGGCTGTCTGTCCTACCAA
D T G Y N K P T F G S G T R L S V L P

Jα32: **gcttttgt**aggggaggaaa**tgctgtg**TGATTCTGGATCCTTAAACAAGCCGACCTTTGGCTCAGGAACCAGGCTGTCCGTCCTACCCA
D S G S L N K P T F G S G T R L S V L P

Jα33: **cgtttattt**aatggacattgt**tgctgtg**AGTGCTGTCTTGAACAGGCTGAACTTTGGAGCAGGAACAAAGCTTTGTGTAATACCAG
S A V L N R L N F G A G T K L C V I P

Jα34: **gcttttgt**aatgggaggaaa**tactgtg**TGGCTATGGCTCTGCATACGGCAGACCAACCTTTGGCACAGGGACCAGGCTGTCTGTCCAACCAA
G Y G S A Y G R P T F G T G T R L S V Q P

Jα35: **gcttttgt**aatgggaggaaa**tgctgtg**TGACTTTGGCTCTGGCAACAAGCTGACCTTTGGCTCAGGAACCAGGCTGTCCGTCCTACCAA
D F G S G N K L T F G S G T R L S V L P

Jα36: **ggttttgc**tagtggatgtgt**tactgtg**TGACTTCAACGTCCACCTGGAAAGCCACCTTTGGAAGTGGGACACAGCTGGTGGTGAAACCTG
T S T S T W K A T F G S G T Q L V V K P

Jα37: **gcttttgt**aatgggaggaag**tgctgtg**TGAATCTGGCTTCACCAAACTGACCTTTGGCTCAGGGACCAGGCTGTCCGTCCTACCAA
E S G F T K L T F G S G T R L S V L P

Jα38: **gcttttgt**tgtgggaagaaa**tgctgtg**TCACTACAGCAGTGACAAGCTGACCTTTGGCTCAGGGACCAGGCTGTCCGTCCTACCAA
H Y S S D K L T F G S G T R L S V L P

Jα39: **catttgtgt**aatggcctttgg**tgctgtg**GGTGCTGAATGGAACAGGCTCACCTTTGGAGCTGGAACAAAGCTTTGTGTAAAGCCTC
G A E W N R L T F G A G T K L C V K P

Jα40: **cattttgt**aatgagacgtgc**cattgtg**TGTCCAATGCGTATGCAGTCACTTTTGGAAAAGGAACTGTACTTTCAGTTGTACCAG
S N A Y A V T F G K G T V L S V V P

Jα41: **ggttttgt**aatgggaggaaa**tggtgtg**TGGCTCTGGCTCTTCATATGGCAAATTGACCTTTGGCTCAGGGACCAGGCTGTCTGTCCATCCAA
G S G S S Y G K L T F G S G T R L S V H P

Jα42: **gcttttgt**aatgggaggaaa**tgctgtg**TGACTAACTTATACAAGCTGACTTTTGGCTCAGGGACCAGGCTGTCTGTCCTACCAA
T N L Y K L T F G S G T R L S V L P

Jα43: **gcttttgt**agtgggaggaaa**tgctgtg**TGATTCTGGATCCTACAACAAGCTGACCTTTGGCTCAGGAACCAGGCTGTCTGTCAAACCAA
D S G S Y N K L T F G S G T R L S V K P

Jα44: **agttttgt**aatgaaaggaaa**tggtgtg**TGGCTCTGGCTCTTCATATGGCAAATTGACCTTTGGCTCAGGGACCAGGCTGTCTGTCCATCCAA
G S G S S Y G K L T F G S G T R L S V H P

Jα45: **cattttgt**aatgagacatac**cacagtg**AGTTAAATGCACGCACAGTCACTTTTGGGAAAGGAACTGTGCTTTCAGTTGTTCCAG
L N A R T V T F G K G T V L S V V P

Jα46: **gcttttgt**aatgggaggaaa**tgctgtg**TGGCTATGGCTCTGGATATGACAGACTGACTTTTGGCTCAGGAACCAGGCTGTCTGTCCAACCAA
G Y G S G Y D R L T F G S G T R L S V Q P

Jα47: **agttcttgt**tgaggcaaaaag**cactgtg**TGAACTACGACAAGTTCACATTTGGGAAGGGAACACGACTTCAGGTTTTGCCTA
N Y D K F T F G K G T R L Q V L P

Jα48: **gcttttgt**aatgggagtaca**tgctgtg**TGAGTGGCAACTACAAGCTGACCTTTGGCTCAGGAACCAGGCTGTCTGTCCTACCAA
S G N Y K L T F G S G T R L S V L P

Jα49: **gcttttgt**aatgggaagaaa**tgctgtg**TGACACTGGCTACAACAAGCTGACCTTTGGCTCAGGGACTAGGCTGTCTGTCCTACCAA
D T G Y N K L T F G S G T R L S V L P

Jα50: **gcttttgt**aggggaggaaa**tgttgtg**TGACTGGCAACAACAACAAGCTGACCTTTGGCTCAGGAACCAGGCTGTCCGTCCTACCCA
T G N N N K L T F G S G T R L S V L P

Jα51: **catttattt**aatggacattgt**tgctgtg**AGTGCTGTCTTGAACAGGCTGAACTTTGGAGCAGGAACAAAGCTTTGTGTAAAGCCTG
S A V L N R L N F G A G T K L C V K P

Jα52: **gcttttgc**aatgggaggaaa**tactgtg**TGGCTATGGCTCTGCATACAACAAACCAACTTTTGGGTCAGGGACCAGGCTTTCTGTCCAACCAA
G Y G S A Y N K P T F G S G T R L S V Q P

Jα53: **gcttttgt**aatgggaggaaa**tgctgtg**TGACTTTGGCTCTGGCAACAAGCTGACCTTTGGCTCAGGAACCAGGCTGTCCGTCCTACCAA
D F G S G N K L T F G S G T R L S V L P

Jα54: **catttgtgt**agtggtctttgg**tgctgtg**AGTAATATTTTGAACAGGCTCATCTTTGGAGCTGGAACAAAACTTTGTGTAAACCGTG
S N I L N R L I F G A G T K L C V N R

Jα55: **catttgtgt**agtggtctttgg**tgctgtg**AGTAATATTTTGAACAGGCTCATCTTTGGAGCTGGAACAAAACTTTGTGTAAACCGTG
S N I L N R L I F G A G T K L C V N R

Jα56: **atttttgt**aatgagatgttc**cacagtg**TATACAATGCGGGCAAAGTCACTTTTGGAAAAGGAACTGTGCTTTCAGTTGTACCAG
Y N A G K V T F G K G T V L S V V P

Jα57: **gcttttgt**aatgggtggaaa**tgctgtg**CGACTATGGGAACAACAAACTGACCTTTGGCTCAGGGACCAGGCTGTCTGTCCAACCAA
D Y G N N K L T F G S G T R L S V Q P

Jα58: **catttttgt**aatgagatatgc**cacagtg**TGTATGATGACCGCACAGTCACTTTTGGAAAAGGAACTGTGCTTTCAGTTGTACCAG
Y D D R T V T F G K G T V L S V V P

Jα59: **tcttttgt**aatgggaagaaa**tgctgtg**TGGCTCTAGCTCTGGATATGGCAAACTAAACTTTGGCTCTGGAACCAGGCTGTCTGTCCATCCAA
G S S S G Y G K L N F G S G T R L S V H P

Jα60: **gattttgt**aatgtgaggaaa**tgctgtg**TGACTATGGGAACTACAAACTGACCTTTGGCTCAGGGACCAGGCTGTCCGTCCAACCAA
D Y G N Y K L T F G S G T R L S V Q P

Jα61: **gcttttgt**aatgggtggaaa**tgctgtg**TGTTTGGCTACAGCCAGCTGACTTTTGGCTCAGGGACCAGGCTGTCCGTCCAACCAA
F G Y S Q L T F G S G T R L S V Q P

Jα62: **cttttgcat**aatggtatttgt**tactgtg**AGTGCTGACAGTAAGACGCTCACCTTTGGAGCTGGAACAAAGGTTTGTGTAAAGCCTC
S A D S K T L T F G A G T K V C V K P

Jα63: **ttttttgt**tatgggaggaaa**tgctgtg**TGGCTATGGCTCTGGATATGGCAAACTGACCTTTGGCTCAGGGACCAGGCTGTCTGTCCAACCAA
G Y G S G Y G K L T F G S G T R L S V Q P

Jα64: **ggttttgt**tggcttgtttt**cattgtg**TGAATGTTGGCTATACCATCATCTTTGGAAAAGGGACAAAGCTTCTTGTGAAACCAA
N V G Y T I I F G K G T K L L V K P

Jα65: **ccattttgt**aaggacctgtca**cacagtg**CTGATTCTGGAGGATGGGGAAAATATACATGGGGGAGTGGAACAAAACTTCTTGTTGCACCAG
D S G G W G K Y T W G S G T K L L V A P

Jα66: **gattttgc**tatggcaaacaa**cacagtg**TGTCTTTGGATACCTACAAGCTAACCTTTGGAGATGGAACAAGGCTCATGGTAAAACCAA
S L D T Y K L T F G D G T R L M V K P

Jα67: **ggatattgt**agaggcctgtgc**cattgtg**TAACTACGGTTACAAATTTACATTTGGATCTGGGACAAGACTTCTGGTTCTGCCAG
N Y G Y K F T F G S G T R L L V L P

Jα68: **catttttgt**agtgattggcat**cagtgtg**CCAATCAGGGATATGTGAAACTGGCATTTGGAGCTGGCACCCAACTTCTTGTTATCCCAA
N Q G Y V K L A F G A G T Q L L V I P

B

```
     nonamer              heptamer          heptamer                          nonamer
Dδ1: tgtttttgtagaaccgtctaccactgtgTTGACTACGTACcacagtgactgaaacagactgaactgctgcacacaaact
                                 L  T  T  Y
                                  .  L  R
                                   D  Y  V

Dδ2: agtttttgtaatgaaatatatcacagtgTTTTGGGATTGGAGTACcacaatgatataaacaatataggcccctgtacaaaaact
                                 F  W  D  W  S
                                  F  G  I  G  V
                                   L  G  L  E  Y

Jδ1: atttattggactaggtattaacagtgtgATAACAGATAAGCTTGTCTTTGGAAGTGGAACCACTCTCACAGTCGAACCAA
                                 I  T  D  K  L  V  F  G  S  G  T  T  L  T  V  E  P

Jδ2: ctttttgatttacccattgtaattgtgTGTTACTGCTCCTCTTATCTTCGGGAAGGGGACTCAACTGACCGTGGAACCAA
                                  V  T  A  P  L  I  F  G  K  G  T  Q  L  T  V  E  P
```

C

```
       nonamer              heptamer          heptamer                           nonamer
Dβ   : tgtttttgtatgaggttgtatcgttgtgGGGACAGGGGATCcacaatgatatattctccaggaggtctttacaaaaaccc
                                   G  T  G  D
                                    G  Q  G  I
                                     D  R  G

Jβ1.1: gcttataatacaacagtttgtcaacgtgACCAACACTAAGGTGATTTTTGGTTTTGGCACAAAAGTTACAGTGATGG
                                   T  N  T  K  V  I  F  G  F  G  T  K  V  T  V  M

Jβ1.2: ggttttactgcagctgcaaaatagtgtgAATAATGAAAAACTGTTCTTTGGCACTGGGACAAAACTCACTGTCATAG
                                   N  N  E  K  L  F  F  G  T  G  T  K  L  T  V  I

Jβ2.1: attttgagagagcctctgcagtactgtgATACATATGAGGTTGAGTTTGGTCCTGGGACCCACATAACAGTACTAG
                                     T  Y  E  V  E  F  G  P  G  T  H  I  T  V  L

Jβ2.2: ctttttagtggcacttcaaaatactgtgCAATCTCAACAACTGCATTTTGGAAAGGGCACCCAGCTGACAGTGCTTG
                                   Q  S  Q  Q  L  H  F  G  K  G  T  Q  L  T  V  L

Jβ2.3: agttttgatattagactgtgagactgtgTTTTATCAATACCCAGTATTTTGGAGAAGGAACAAAAATAACAGTTCTGG
                                    F  I  N  T  Q  Y  F  G  E  G  T  K  I  T  V  L

Jβ2.4: cattttggatcaaaaataagatagtgtgTTATCAAAGACAGAGAGTACATTTTGGAGGAGGAATACACTTTAAAGTTCTGT
                                    Y  Q  R  Q  R  V  H  F  G  G  G  I  H  F  K  V  L
```

D

```
      nonamer             heptamer
Jγ1: gcttattgcaaagtcaaaacatagtgtgGACCACATGGATTAAATACTTCGGTACAGGAACTAAGCTAATTATCTCCG
                                  T  T  W  I  K  Y  F  G  T  G  T  K  L  I  I  S

Jγ2: agatattgctaagtagtatcagactgtgGGATGCTGATGTTAAAATATTTGGAACTGGAACTAAGCTTATTGTTTCAG
                                  D  A  D  V  K  I  F  G  T  G  T  K  L  I  V  S

Jγ3: tgttattgcaaagactttatgcactgtgTCTGGTTATTACTACAAAGTGTTTGGCACCGGTACAAAGCTCATCGTGTCAG
                                 S  G  Y  Y  Y  K  V  F  G  T  G  T  K  L  I  V  S

Jγ4: ggtttgtgtaaggaaatatagcagtgtgGGCAATCTGGTCTTTGGCACAGGCACTAAACTCATCGTTTCCA
                                 G  N  L  V  F  G  T  G  T  K  L  I  V  S

Jγ5: aatttgtgtaaggcaatatagcagtgtgGGCAATCTGGTCTTTGGCACAGGCACTAAACTCATCGTTTCCA
                                 G  N  L  V  F  G  T  G  T  K  L  I  V  S
```

E

```
     nonamer             heptamer                heptamer                       nonamer
Dδ2: acttttgtaactaagtatatcactgtgTTGAGAACTTGGAGTAGcacaatagcacaaacactctaaggcctcatacaaaaacc
                                 L  R  T  W  S
                                  .  E  L  G  V
                                   E  N  L  E  .

Jδ2: tgttttctgactatgcagaaacactgtgGAAACAGACAAGCTTGTATTTGGAAGTGGGATTGCTTTCTCAGTTGAACCAA
                                 E  T  D  K  L  V  F  G  S  G  I  A  F  S  V  E  P
```

Supplementary Figure S1 Nucleotide and deduced amino acid sequences of duck D and J gene segments, Jα segments (A), Dδ and Jδ segments (B), Dβ and Jβ segments (C), Jγ segments, and D and J segments in the TCRδ2 locus (E).

A

<----------FR1-IMGT---------><CDR1-><----FR2-IMGT---><CDR2-><------------FR3-IMGT------------>

$V_{\alpha}1.2$: HAQ-DSVVQFAQETAIQVGHNATLHCNFSTSSSLPYIFWYQQHLTQSPQFLLQVSKFKPHVHSERISSVLSMENSQVLLHVQDAKLQDSAVYLC
$V_{\alpha}1.3$: HAQKDTVVQFAQETAIQVGHNATLHCNFSTSTISPYIFWYQQHLTQSPQFLLQVSKFKPHVHSERISSVLSMENSQVLLHVQDAKLQDSAVYLC
$V_{\alpha}1.4$: HAQKDSVVQFAQETAIQVGHNATLHCNFSTSTSTPYIFWYQQRLTQSPQFLLQVSKFKPHVHSERISSVLSMENSQVLLHVQDAKLQDSAVYLC
$V_{\alpha}1.5$: HGQKDSVVQFAQETAIQVGHNATLHCNFSTSFSAPYIFWYQQRLTQSPQFLLQVSKFKPHVHSGRISSVLSMENSQVLLHVQDAKLQDSAVYLC

$V_{\alpha}2.1$: AAGRAQVQQ-EPWAQTTEGTGINIACSHPNIQSNDYIYWYRQFPGQGPAFLVLAKSGSRDVTDPAGRLSVAGDRRSSTLLLTQPRLRDTAVYYC
$V_{\alpha}2.2$: AAGRAQVQQ-EPWSQTREGSGIDIACSHPNIQSNDYIYWYRQFPGQGPAFLVTAYSRSQDVTDPAGRLSVAGDRRSSTLRLTRPRLRDAAVYYC
$V_{\alpha}2.3$: AAGRAQVQQ-EPWAQTREGSGIDIACSHPNIQSNDYIYWYRQFPGQGPAFLVRAYSGSEDVTDPAGRLSVAGDRRSSTLRLARPRLRDAAVYYC
$V_{\alpha}2.4p$: AAGRAQVQQ-EPWAHTTEGTGINIACSHPNIQSNDYIYWYRQFPGQGPAFLVWAKSGSRDVTDPAGRLSVAGDRRSSTLLLT*PRLRDAAVYYC
$V_{\alpha}2.5$: AVCRAQVQQ-EPWAETREGTGINITCSHPNMQTGYSIYWYRHLPGQGPTFLVFGTRGSNALRDLPGWLVVAADRRSSALWLTDPRLRDAAVYYC
$V_{\alpha}2.6$: AVCRAQVQQ-EPRAQTTEGTSINITCSHPNMQTGYSIYWYRHLPGQGPTSLVFGTRGSNPLRDLPGWLVVAADRRSSALWLTDPRLRDAAVYYC
$V_{\alpha}2.7$: AVCRAQVQQ-EPWAETRENTGINITCSHPNIQTGEVIYWYRHLPGQGPASLAFGTRGSNALRDLPGWLVVAADRRSSALWLTEPQLRDAAVYYC
$V_{\alpha}2.8p$: AAGRAQVQQ-EPRAQTTEGTGINIACSHPNIQSSDYIYWYR*FPGREPTFLVWAYSGSKDVRNPAGRLSVTGDRRSSALWLTDPRLRDAAVYYC
$V_{\alpha}2.9$: AVCRAQVQQ-EPWAQTTEGTSINITCSHPNIQIGDSIYWYRHVPGQGPTFLVLGTKGSNPLRDLPGWLVVAADRRSSALWLTDPRLRDAAVYYC
$V_{\alpha}2.10$: AVGRAQVQQ-EPWAETTEGTGINITCSHPNIQTGEVIHWYRHLPGQGPAFLVFGTGGSRALTDLPGWLVVAADRRSSALWLTKPRLRDAAVYYC
$V_{\alpha}2.11$: AVCRAQVQQ-EPQAQTTEGTSINITCSHPNMQTGYSIYWYRHLPGQGPTFLVFGTKGSNALRDLPGWLVVAADRRSSALWLTEPRLRDAAVYYC
$V_{\alpha}2.12$: AVCRAQVQQ-EPWAETREGTSINITCSHPNIQTGEVIHWCRHLPGQGPAFLMSAFSGSRELTDLPGWLVVAADRRSSALWLTDPRLRDAAVYYC
$V_{\alpha}2.13$: AVCRAQVQQ-EPRAQTTEGTSINITCSHPNIQTGEVIYWYRHLPGQGPASLAFGTRGSNALRDLPGWLVVAGDRRSSALWLTDPRLRDAAVYYC
$V_{\alpha}2.14$: AAGRAQVQQ-KPWAQTREGTGINIACSHPNIQSFDIIYWYRQFPGREPAFLVSAQSGSRDVTDPAGRLSVAGDRRSSTLWLARPRLRDAAVYYC
$V_{\alpha}2.15$: AVCRAQVQQ-EPRAQTTEGTGINIACSHPNIQSYDIIYWYRHLPGREPAFLVSAQSGSRDVTDPAGRLSVAGDRRSSALWLARPRLRDAAVYYC
$V_{\alpha}2.16$: AVCRAQVQQ-EPWAQTTDSTGINITCSHPNIQITEVIYWYRHLPGQGPAFLMSAFSGSRELTDLPGWLVVAADRRSSALWLTKPRLRDAAVYYC
$V_{\alpha}2.17$: AAGRAQVQQ-EPWAQTREGSGIDIACSHPNIQSNDYIYWYRQFPGQGPAFLVRAYSGSEDVTDPAGRLSVAADRRSSALWLTDPRLRDAAVYYC
$V_{\alpha}2.18$: AAGRAQVQQ-EPWAQTTEGTGINIACSHPNIQSNDYIYWYRQFPGQGPAFLVLAKSGSRDVTDPVGRLSVAADRRSSTLWLARPRLRDAAVYYC
$V_{\alpha}2.19$: AVCRAQVQQ-EPRAQTREGSGIDITCSHPNIQSYDIIYWYRQFPGREPAFLVSARSGSQDVTDPAGRLSVAADRRSSALWLTKPRLRDAAVYYC
$V_{\alpha}2.20$: AAGRAQVQQ-EPRAQTREGSGINIACSHPNIQSYDYIYWFRQFPGRGPAFLVSARSGSQDVTDPAGRLSVAGDRRSSALWLTQPRLRDAAVYYC

$V_{\alpha}3.1p$: AMGQTSVTQQEGQVTVKQKETFQTTCTYQIPSLYA-LY*YQQKKGQAPQLVIYHTRAG-TKQSGRFTMELNTVGKSSILRLKEVELSDSALYLC
$V_{\alpha}3.2$: AMGQTSVTQQEGQVTVKQKETFQTTCTYKSSYFYA-LYWYQQKKGQAPQLVIYHTSAG-TKQSGRFTMELNTVGKSSILRLKEVELSDSALYLC
$V_{\alpha}3.3$: AMGQTSVTQQEGQVTVKQKETFQTTCTYQIPSLYG-LYWYQQKKGQAPQLLAYYTTTG-SMQNIHFTMEMNTVGKSSILQLKEAELSDSALYLC
$V_{\alpha}3.4$: AMGQTSVTQQEGQVTVKQKETFQTTCTYQIPTFYA-LYWYQQKKGQAPQLVTYHTEAG-TKQSGRFTTGLNTVGKSSILRLKEVELSDSALYLC
$V_{\alpha}3.5$: TTGQVSVTQEEGQVSVEQGNTFQTNCTYETSSFNG-LLWYEQKKGQAPQLITHRAIAG-TKQKDRFTTELNTKGKSSVLRLKEVELSDSALYLC
$V_{\alpha}3.6$: ATGQVSVIQQEGQVSVEHGNTFQTNCTYETSNFNG-LLWYQQKKGQAPQRISYQAGAG-IKQIDRFTTELNTSDKSSVLQLKEVELSDSALYLC
$V_{\alpha}3.7$: IMGQVSVTQQEGQVTVEQGNAFETTCTYETSYFRG-LLWYEQKKSQGPQLLSNQAIAG-TKQGDCFTTELNITEKSSVLQLKEVELSDSALYLC
$V_{\alpha}3.8$: TMGQVSVKQQEGQVTVEHRNTFQTTCTYQSSNFQG-LFWYQQKKGQAPQLITRQAGAG-TKQRDRLTTELNTERKSSVLQLKEVELSDSALFFC
$V_{\alpha}3.9$: TTGQVSVVQQEGQVTVEQGNTFQTNCTYETSNFKG-LLWYQQKKGQAPQLISYQAGAG-TKQRDRLTTELNTERKSSVLQLKEVELSDSALFFC
$V_{\alpha}3.10$: TMGQLSVIQQEGQVTVEQGNTFQTHCTYQSSTPDA-WLWYQQKKGQAPQRISYQAGAG-TKQSDRLTTELNTERKSSVLCLKEVEPSDSALYFC
$V_{\alpha}3.11p$: TMGQVSVKQQEGQVTVEHRNTFQTTCTYQSSNFQG-LFWY*QKKGQVPQLISHQGIVG-TKQKDRFTTELNTERKSSVLQLKEVELSDSALYLC
$V_{\alpha}3.12$: TMGQVSITQEEGQVSVEQGNTFQTNCTYETSYFQG-LLWYKQKKGQAPQLITHQAVAG-TKQKDRFTTELNTKGKSSVLHLKEVELSDSALYLC
$V_{\alpha}3.13$: TTGQVSVIQQEGQVSVEHGNTFQTNCTYETSNFNG-LLWYQQKKGQAPQRISYQAGAG-TKQIDRLTTELNTERKSSVLRLKEVELSDSALYFC
$V_{\alpha}3.14$: TTGQVSVTQQEGQVSVEHGNTFQTNCTYQSSTPDA-WLWYQQKKGQAPQLISYQAGAG-TKQRDRLTTELNSERRSSVLRLKEVKLSDSALYFC
$V_{\alpha}3.15$: TMGQLSVIQQEGQVTVEQGNTFQTHCTYQSSTPDA-WLWYQQKKGQAPQLVVYHTSAG-TKQSGRFTMELNTERKSSILRLKEVELSDSALYLC
$V_{\alpha}3.16$: -MGQTSVTQQEGQVTVKQKETFQATCTYQIPSLYA-LYWYQQKKGQAPQLVIYHTSAG-TKQSGRFTMELNTERKSSVLRLKEVELSDSALYLC
$V_{\alpha}3.17$: TVEQVSVTQEEGQVSVEQGNTFQTNCTYETSNFNG-LLWYQQKKGQAPQRISYQAGAG-TKQSDRLTTELNTKGKSSVLHLKEVELSDSALYLC
$V_{\alpha}3.18$: IMGQVSVTQQEGQVSVEHGNTFQTNCTYETSYFRG-LLWYKQKKSQGPQLLSNQAIAG-TKQGDRFTTELNITEKSSVLQLKEVELSDSALYLC
$V_{\alpha}3.19$: TTGQVSITQHEGQVTVEQGNTFQTNCTYETSRFNG-LLWYQQKKGQAPQRISYQAGAG-TKQSDRLTTELNTERKSSVLRLKEVELSDSALYFC
$V_{\alpha}3.20$: TTGQVSVIQQEGQVSVEQGNTFQTNCTYQSSTPDA-WFWYQQKKGQAPQRISYQAGAG-TKQKDRLTTELNTERKSSVLRLKEVELSDSALYFC
$V_{\alpha}3.21$: TTGQVSVIQQEGQVTVEQGNTFQTNCTYETSNFNG-LLWYQQKKGQAPQLITRQAGAG-TKQKDRLTTELNTERKSSVLHLKEVELSDSALYFC
$V_{\alpha}3.22$: TMGQVSVTQEEGQVSVEQRNTFQTNCTYQSSNFYG-LFWYQQKKGQAPQRISYQAGAG-TKQNGRFTTELNTSDKSSVLQLKEVELSDSALYLC
$V_{\alpha}3.23$: TTGQVSVTQEEGQVSVKQGNTFQTNCTYETSRFYG-LLWYEQKKGQAPQLISYQAGAG-TKQRDRFTMELNTEEKSSVLRLKEVKLSDSALYLC
$V_{\alpha}3.24$: TTGQVSVTQQDGQVTVEQGNTFQTNCTYETSNFDG-LPWYQQKKGQAPRLITRQAGAG-TKQKDRFTTELNTERKSSVLQLKEVELSDSALYFC

B

<----------FR1-IMGT---------><-CDR1-><----FR2-IMGT---><-CDR2-><--------------FR3-IMGT------------->

$V_{\delta}1$: VCAELMLVESGGRLR-VPGESVHLSCWGSDFNFAIYSVLWYRQALGGSLQWLSYIN-PDSSYIRYGSAVKGRATASRDNSKSKTALTLNNLHLQDSARYFC

$V_{\delta}2.1$: VWAQWRLVESGGGVK-TPGNSVKLSCEGSGFTFSSFAIRWYRQAPGGSLEWVSFIN-TYGSTVEYGKAVKGRASVSRDNSQSKSSLSLSSLVPQDSAHYFC
$V_{\delta}2.2$: VWAQLSLVESGGGLR-APGDSVHLSCQGSGFSFKDYYVYWYRQAPGGSLEWVSFIS-SGGSTVEYGKAVKGRASVSRDNSQSKSSLSLSSLVPQDSAQYFC
$V_{\delta}2.3$: VWTQLSVVESGGGLR-APGESVHLSCQGSGFSFKDYYVLWYRQAPGGSLEWLSFN--YDDSTVEYGKAVEGRASISRDNSQSKSSLSLSSLVPQDSAHYFC
$V_{\delta}2.4$: VWTQLSLVESGGGLR-APGDSVQLSCQGSGFTFGSYYVLWYRQAPSGSLEWVSFID-FYGSTVEYGKAVKGRASVSRDNSQSKSSLSLSSLVPQDSAQYFC
$V_{\delta}2.5$: VWAELKLVESGGGLR-APGDSVQLSCQGSGFTFGNYHVWWYRQAPGGSLEWVSFIY-YDGSTVEYGKAVKGRASVSRDNSQSKSSLSLSSLVPQDSAQYFC
$V_{\delta}2.6p$: VWAQWKLVESGGGLQ-AAGNSVHLSCQGSGFNFRSYHVLWYRQAPFGRIEWVSFIN-AFGNTKDYGKVVEGRANVSRDNSQSKAYMSLHALLSQDSARYFS
$V_{\delta}2.7$: VWAELKLVESGGGLR-APGDSVHLSCQGSGFTFENYHVLWYRQAPSGSLEWVSFIN-YDGRIREYDKAVEGRASVSRDNSQSKSSLSLSALVPQDSAQYFC
$V_{\delta}2.8$: VWTQFSLVDSGGGLP-APGDSVHLSCQGSGFPFGSYHVLWYRQAPGGSLEWVSYIN-YQGRITKYGKAVEGRAIVSRDNSQSKSSLSLSSLVPQDSAHYFC
$V_{\delta}2.9$: VWAQWRLMVSGGGLR-APGDSVHLSCQGSGFPFGSYLVLWYRQAPGGSLEWVSYIN-SQGGIKKYGKAVEGRASVSRDNSQSKSSLSLRALVPKDSAHYFC
$V_{\delta}2.10$: VWAQLIVVESGGGLR-AAGDSVQLSCQGSGFTLGNHDVWWYRQAPGGTLEWMSYLN-YDGSTLEYSKAVEGRASVSRDNSQSKAYLSLRALLPQDSAHYFC
$V_{\delta}2.11$: -AAQWKLLESGGGLR-APGDSVQLSCKGSGFRFQDHSVWWYRQAPVGSLEWVSYIK-FDVRTTKYGKAVEGRVTASKDNSQSKAYLSLHALLPQDSAHYFC
$V_{\delta}2.12$: VWAELKLVESGGGLR-APGDSVQLSCQGSGFTFGNYHVWWYRQAPGGSLEWLSFIN-TYGSTVEYGKAVEGRASVSRDNSQSKSSLSLSSLVPQDSAHYFC
$V_{\delta}2.13$: VWTQLSVVESGGGLR-APGDSVHLSCQGSGFTFGSYHVLWYRQAPGGSLEWVSYIN-YQGRITKYGKAVEGRAIVSRDNSQSKSSLSLSSLVPQDSAHYFC
$V_{\delta}2.14$: VWAQWRLMVSGGGLR-APGDSVHLSCQGSGFTFGSYYVLWYRQAPGGSLEWVSYIN-SQGGIKTYGKAVEGRAIVSRDNSLSKSFLSLHALVPKDSAHYFC
$V_{\delta}2.15$: VCSELKLVESGGGLR-APGDSVQLSCQGSGFSFQDYAILWYRQAPGGSLEWVSFI--YYD--AEYGKAVEGRASVSRDNSQSKSSLSLSSLVPQGSAHYFC
$V_{\delta}2.16$: -WAELKLVESGGGLR-APGDSVHLSCQGSGISFKDYHVYWYRQAPGLSLEWVSFIS-YDGSTVEYGKAVKGRASVSRDNSQSKSSLSLSSLVPQDSAHYFC
$V_{\delta}2.17$: IWAELKLVESGGGLR-APGDSVHLSCQGSGFTFENYPVLWYRQAPGGSLEWVSFIN-FYGSTVEYGKAVKGRASVSRDDSQSKSSLSLSSLVPQDSAHYFC
$V_{\delta}2.18$: VWAELKLVESGGGLQ-AAGNSMQLSCQGSGLSFEYYDVWWYRQAPGGNLEWMSYLN-YDGSTVEYSKAVEGRASVSRDNSQSKSSLSLSALEPQDSAYYFC
$V_{\delta}2.19$: VWAQS-LVESGGGLR-APGDSVHLSCQGSGFTFGSYPVWWYRQARGGSLEWLSAIT-YDGSSVQYGKAVKGRATASRDNSQSKSSLSLSSLVPQDSAHYFC
$V_{\delta}2.20$: VWAELKLVESGGGLR-APGDSVQLSCQGSGFRFQDFSVWWYRQALGGSLEWVSYIN-FDVRTTKYGKAVEGRVTASKDNSQSKAYLSLRALLPQDSAHYFC

$V_{\delta}3.1$: VWTQLSLVESGGGLR-ASGDSMQLSCQVSVPNFEDYSVRWFRQARGGGLEWVSYISSRSGTVKQYGATVDGRAAASRNNSQAKSSLSLQHLHVGDSGRYFC
$V_{\delta}3.2$: VWAQWRLVESGGGVQ-APGNTVHLSCRGSGFTFSSFAIQWYRQAPDGCLEGVSYISSRSGSTKQYGAAVQGRATASRDNSRSESSLSLQHLHVGDSGRYFC
$V_{\delta}3.3$: VWAQLSLVESGGGLR-APGDSVRLSCQGSGLSFEDYYVLWYRQARGGGLEWVSYISGPSGTVLKYGAAVEGRATASRDNSRAESSLSLQHLHVGDSGRYFC
$V_{\delta}3.4$: VWAQLSLVESGGGLR-APGDSVRLSCQGSGLSFEDYYVLWYRQARGGGLEWVSYISGPSGTEKQYGAAVEGRATASRDNSRSKSSLSLQHLHVGDSAHYFC
$V_{\delta}3.5$: VWAQWRLVESGGGVK-TPGNSVHLSCQGSGFTFSSFAIRWYRQAPGGSPEWVSYIGGLSGATRQYGAAVQGRATASRDNSRSESSLFLQHLHVGDSGRYFC
$V_{\delta}3.6$: VWAQWRLVESGGGVR-TRGNSVNLSCHGSGFNLSLFAIQWYRQAPGGSLEWVSYISMPYGTEKQYGAAVQGRATASRDNSRAESSLSLHPLHVGDSARYFC

$V_{\delta}4.1(4.2)$: --QGDEIQPTRTTVWGQAGQTETLQCT-YSSNASYIYVAWYRQHPNGSLQYLLQSK-GRGGSYIHTAPFARKRFSGKAD-ESSGTLFIHALELKDNALYYC

$V_{\delta}5$: VWAQPRVVESGGGLR-APGDSVLLSCQGYALTLINYYFWWYRQAPSGRLECLSYIS-YDSSDIHYLPGIKGRAMTSRNNSRSVAFLLLHGLHPQDSAHYFC

C

```
                  <--------FR1-IMGT-------><CDR1-><-----FR2-IMGT----><CDR2-><-------------FR3-IMGT------------->

Vβ1.1           : KLITQWPEETLKRAGETVDISCYQNNSHQLAYMFWYQQPPG--SSLKLVASTSLWLQKSYAEGYSEAKFEISRDNSELSVMTIKNVTRKDAATYFC
Vβ1.2p(1.3/1.4) : KLITQWPEETLKRAGETVDISCYQNNSHQLAYMFR*QQPPG--SSLKLVASTSLWLQKSYAEGYSEAKFEISRDNSELSVIAIKNVTRKDAATYFC

Vβ2             : WALQQSPDTVVYE-GDALILNCSEKKESAFLTMYWYKLPVGKNATLQLIVNSVEGGRAEIEKEFIN-HFQSSGTKSSCLSVETTHVLLNDSGTYFC

Vβ3.1(3.4)      : SALEQSPDTVIWQ-GEKMSLTCS-KMTPSYIYMYWYKMPLGNGSGMILMVTAIKGSKASVEAAFQS-HFTSSGIQGDGMTLSTEGAFLNDSGIYYC
Vβ3.2           : LALEQSPDTVIWQ-GEKMNVICS-KMKSSSTVMYWYKMPLGNGSGMILMVTAIKGVKASVEAAFQS-HFMSSDIQGDGMTLSTEGAFLNDSGIYYC
Vβ3.3           : SALEQSPDTVIWQ-GEKMSLTCS-KMKPSYYNMYWYKMPLGNGSGMILMVTAVKGAKASVEAAFQS-HFMSSGIQGDGMTLSTEGAFLNDSGIYYC
Vβ3.5           : LALEQSPDTVIWQ-GEKMSLTCS-KMNPSYYTMYWYKMPLGNGSGMILMVTAVKGAKASVEAAFQS-HFTSSGIQGDGMTLSTEGAFLNDSGIYYC
Vβ3.6           : LALEQSPDTVIWQ-GEKMSLTCS-KMKSSSTVMYWYKMPLGNGSGLILVVTALKVGKASVEAAFQS-HFTSSDIQGDGMTLSTEGAFLNDSGTYYC
Vβ3.7           : LALEQSPDTVIWQ-GEKMSLTCS-KMKPSSDIMYWYKMPLGNGSGLILVVTAVKVGKASVEAAFQS-HFTSSGIQGDGMTLSTEGAFLNDSGIYYC

Vβ4             : AKITQTSSLVLKEDGE-ATLKCS--QNDNHNYMSWYLQQPG--KGLQLLYYSIGADQEAVGDTHPG--YKATRLNLSDFHLVIKPVKMNHSADYFC
```

D

```
          <-----------FR1-IMGT----------><-CDR1-><---FR2-IMGT----><-CDR2-><---------------FR3-IMGT------------>

Vγ1.1   : GFAQEIPIQTPVSITMSQG-TARLQCHFKDVSANFDNTVIHWYQQKENREPVRMLYISSGTTTVENNFQ-RNRYMVQNLSNQKICILIIKNIGPDDAATYYC
Vγ1.2   : GFAQEIPIQTPISITKSQG-STRLQCHFKEISANFDSTTIHWYQQKENKAPEWMFYISTGTTKADKSLQ-GRTYTIERVSRQKICTLTIKNIAPDAAATYYC
Vγ1.3   : GLAQEIPIQTPVSITKSQG-STRVLCHF*RVSANFDNTIIHWYQQKGNKALEWMFYVTTRITRADKSFQ-GRRYTVETVSDQKMCTLIIKSIVPDDTATYYC
Vγ1.4   : GFAQEIPIQAPISITKFQG-STRLQCHFKDVSTNFDSIVIHWYQQKENKAPEWMFYITTGKTSVEENFQ-GRTYTIERISRKKICTLTISNIIPDDTAIYYC
Vγ1.5p  : VFAQEIPTQTPISITKSRG-TTRLQCHFKGVSTNFDDTIIH*YQQKENEAPERMFYILTGTTAVDESFQ-SRTYTIERVSKQKICTLIIKSIVPDDTATYYC
Vγ1.6   : GFAQEIPIQAPISITKFQG-STRLQCHFKDVSTNFDSIVIHWYQQKENKAPEWMFYITTGKTAVDESFQ-GRTYTIERVSKQKICTLTIKNIAPDVAATYYC

Vγ2.1   : VFAQEIPIQTPISITKFKK-TARMYCEIKNLPTSFDSTVIHWYKRKENEAPERLLFFAEGSTSVENGFQ-KDRYRAERVSDQNRCVLMIKDVIPDDAGMYYC
Vγ2.2p  : GFSLEIPLQNSISISKFKK-IS*LSCEIKSLPTIFDSTVLHWHKWKENEAPERLLLFAEGKTSVESGFQ-RDRYRAERVSEQNRCVLMTRDVIPADTSTYH-

Vγ3.1   : GDTQAVPVQTLVTRTQAEGRSASMACQLT------KEDTVHWYKQLPGEPPKRILYVSGQTPVFDDSSD-RQKYQVRKRASEPLYTLQINNVNQRDAGTYYC
Vγ3.2p  : GDTQAVPVQSPAMRTQAEGRSASMACRLT------KEDTVHWYKQLPGQPIKRILYVSGQIPAFDDSSD-RQKYQVRKRASEPLYSLQINNVNQRDAGT*YC
Vγ3.3p  : GDAQAVPVQSPAMQRQVKSISARMECRLS------SNAIIHWYKQLPGEPPKRILYMSEMNPVFDDGSD-RQKYQV*KRASEPLYSLTIDFLTQRDAGTYYC
Vγ3.4   : GDAQVVPVQSPAVRRQVKGSSARMECRLS------SNTIVHWYKQLPGEPPKRILYVSGQSPTFDDGSD-GRKYQVWKHASQPLYSLTIDFLNQRDTGTYYC

Vγ4     : --QVLLQQRQPSLTKRTSS-TAVIDCKVEGID-DFQDAYIHWYRHIPSRAPERLLYVTSTAQISYDKDSYKDKYHSSK-RGKNTCTLSVKDIREDDEGTYYC

Vγ5     : SPARALQQSPVSVTKPESK-TVLITCHVS-VP-DFDKAFIHWYRTRPGAAPERVAYMASTLFLANRED--EGKFSIEKDVGKSLCTLTVTKVTLRDAGTYYC

Vγ6     : GAAQILRQPKPSVRRVAYS-TARIDCHFS-GS-DFPNAYIHWYQQKPGEAPQHLLYVQKEAAAYYDQS-YRETFGAEKKKTEPICTLTIKRVTKEQEATYYC
```

Supplementary Figure S2 Amino acid sequence alignments of the duck V genes, Vα segments (A), Vδ segments (B), Vβ segments (C) and Vδ segments (D). Only functional genes and in-frame pseudogenes are shown. The FRs and CDRs were described according to the IMGT unique numbering for the V-REGION. Canonical cysteines and tryptophans are shaded. The pseudogenes are indicated by “p”.

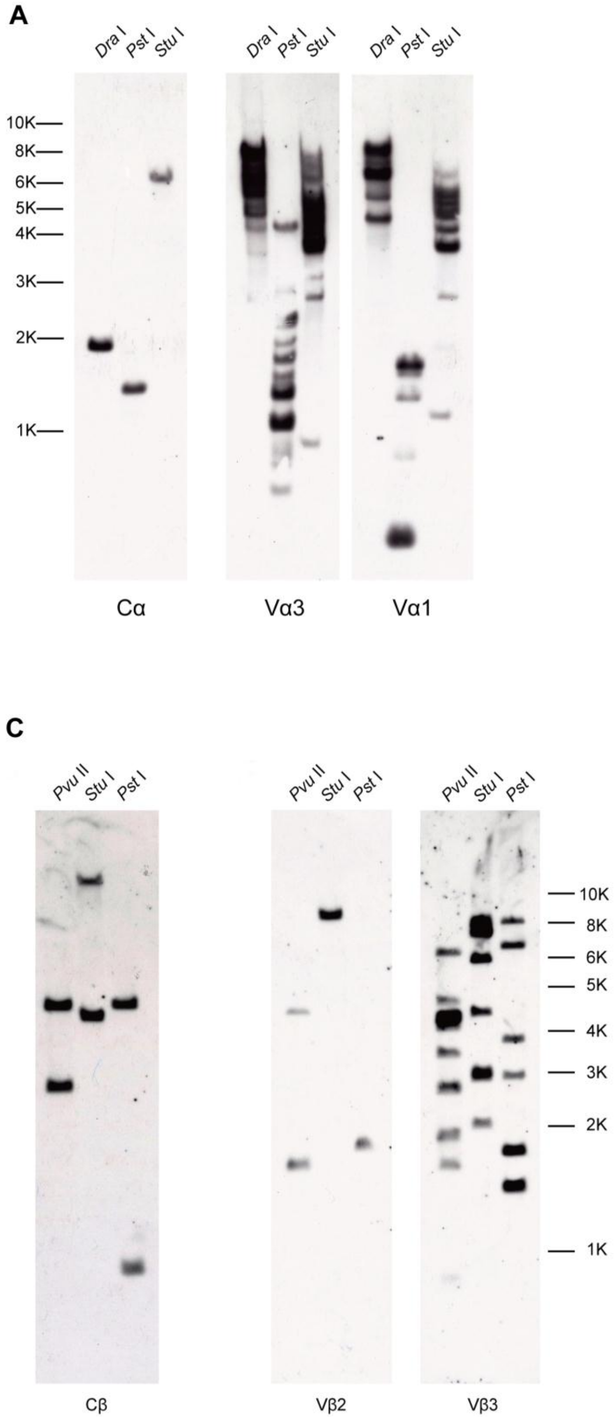
A
Dra I
Pst I
Stu I
10K
8K
6K
5K
4K
3K
2K
1K
Cα
Vα3
Vα1
B
Cδ
Vδ2
Vδ5
C
Pvu II
Cβ
Vβ2
Vβ3

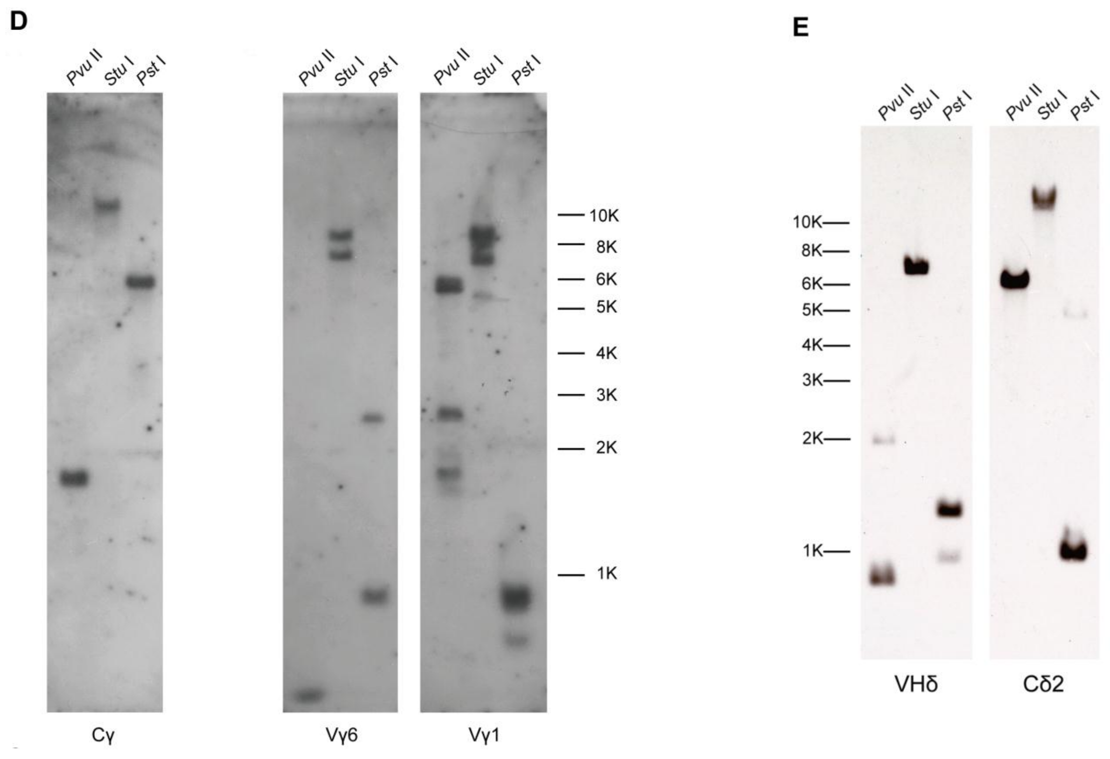

Supplementary Figure S3 Southern blotting detection of duck genomic DNA using the TCR C and V specific probes, Cα and Vα (A), Cδ and Vδ (B), Cβ and Vβ (C), Cγ and Vγ (D), and Cδ2 and VHδ (E). The probes used are indicated below each panel. The restriction enzymes used are indicated above each panel. The DNA marker is shown on the left.

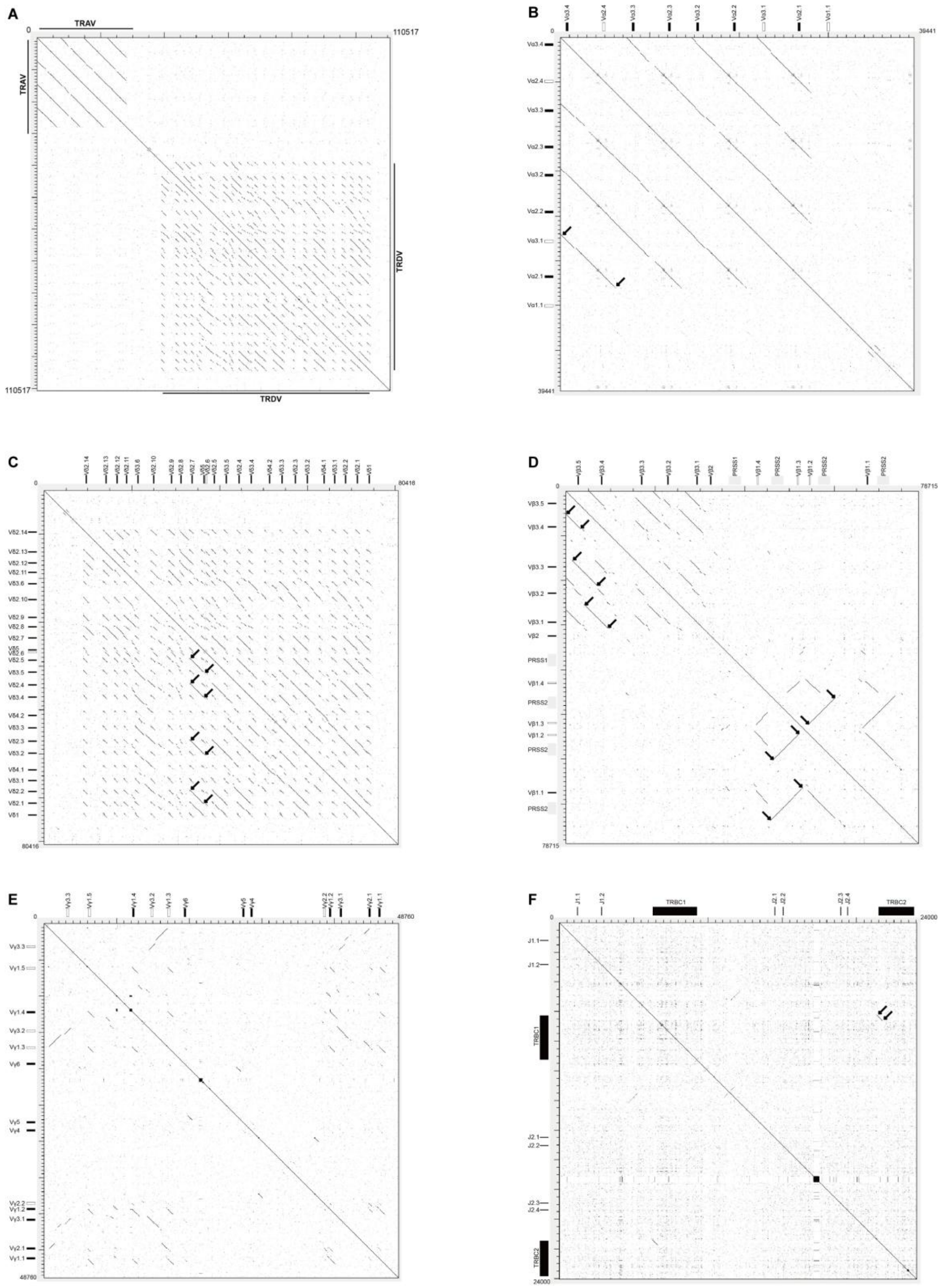

Supplementary Figure S4 Dotplot analyses of the V regions of TCRα/δ, TCRβ and TCRγ. Approximate V segment locations are shown on each axis. Functional V segments, pseudogenes and non-TCR genes are marked as in Fig. 1. (A) The Vα/δ

region of TCRα/δ locus. (B) The incomplete Vα region of TCRα/δ locus. Lines parallel to the main diagonal reflect the homology unit containing one Vα2 and one Vα3 segment. (C) The Vδ region of TCRα/δ locus. The repeated unit of ~ 4 kb length composed of V segments from Vδ2 and Vδ3 subgroups. (D) The Vβ region of TCRβ locus. The 5' duplicated region corresponds to Vβ3 subgroup. One *PRSS2* gene and one Vβ1 segment formed a homology unit, which has three copies. Lines perpendicular to the main diagonal reflect the *PRSS2* gene and Vβ1 segment with an opposite transcriptional orientation to the relevant C region. (E) The Vγ region of TCRγ locus. (F) The J-C region of TCRβ locus. Except the well-conserved first exon of two Cb genes, no homologous regions containing coding sequences were found in J-C region of TCRb locus. The solid black region on the diagonal reflects an ~400 bp area of short tandem repeats.

A

```
                                                                                                                              CDR3(nt)
          Y  L  C                                                                  F  G  S  G
Vα3.4    TACCTCTGTGCTGTGAATGA--------------------------TGACTGGCAACAACAACAAGCTGACCTTTGGCTCAGGAACCAGGCTGTCCGTCCTACCCA  Jα50
Vα3.4-1: TACCTCTGTGCTGTG-------CGTGATCGATCTGGATCCTTA-----------------AACAAGCTGACCTTTGGCTCAGGAACCAGGCTGTCCGTCCTACCCA              30

                                                                                   F  G  K  G
                                                          CGTACACTGAGGGCAAAGTCACTTTTGGAAAAGGAACTGTGCTTTCTGTAGTTCCAG  Jα6
Vα3.4-2: TACCTCTGTGCTGTGA-------------TCG-----------------CGTACACTGAGGGCAAAGTCACTTTTGGAAAAGGAACTGTGCTTTCTGTAGTTCCAG              24


                                                                                   F  G  S  G
             Y  L  C                                  TGACTTTGGCTCTGGCAACAAGCTGACCTTTGGCTCAGGAACCAGGCTGTCCGTCCTACCAA  Jα35
Vα1.2-1   : TACCTGTGT  GCACTGGCCGG-------------------------TGGCTCTGGCAACAAGCTGACCTTTGGCTCAGGAACCAGGCTGTCCGTCCTACCAA  (*)         24

                                                                                   F  G  S  G
             Y  L  C                              TGGCTATGGCTCTGGATATGACAGACTGACTTTTGGCTCAGGAACCAGGCTGTCTGTCCAACCAA  Jα46
Vα1.2-2   : TACCTGTGT  GCACTGAGCCGGCGCCT-------------CTATGGCTCTGGATATGACAGACTGACTTTTGGCTCAGGAACCAGGCTGTCTGTCCAACCAA              34
Vα1.2-4   : TACCTGTGT  GCACTGAGCCCCGGGGA-------------CTATGGCTCTGGATATGACAGACTGACTTTTGGCTCAGGAACCAGGCTGTCTGTCCAACCAA              34
Vα1.2-5   : TACCTGTGT  GCACTGAGGGGGC------------------ATGGCTCTGGATATGACAGACTGACTTTTGGCTCAGGAACCAGGCTGTCTGTCCAACCAA  (*)         30

                                                                                   F  G  S  G
             Y  L  C                                         TGAACTACGAGAAGCTCACATTTGGGAAGGGAACACGACTTCAGGTTTTGCCTA  Jα29
Vα1.2-3   : TACCTGTGT  GCACCCCCGGGGAAG-----------------------TGAACTACGAGAAGCTCACATTTGGGAAGGGAACACGACTTCAGGTTTTGCCTA  (*)         24

                                                                                   F  G  S  G
             Y  L  C                                      TAACTACGGTTACAAATTTACATTTGGATCTGGGACAAGACTTCTGGTTCTGCCAG  Jα67
Vα1.2-6   : TACCTGTGT  GCGAGCCGCTC-----------------------------CTACGGTTACAAATTTACATTTGGATCTGGGACAAGACTTCTGGTTCTGCCAG              21

                                                                                   F  G  S  G
             Y  L  C                                        TGAATCTGGCTTCACCAAACTGACCTTTGGCTCAGGGACCAGGCTGTCCGTCCTACCAA  Jα37
Vα1.2-7   : TACCTGTGT  GCACTGAGGAC----------------------------CTGGCTTCACCAAACTGACCTTTGGCTCAGGGACCAGGCTGTCCGTCCTACCAA  (*)         22

                                                                                   F  G  S  G
             Y  L  C                                     TGACTTCAACGTCCACCTGGAAAGCCACCTTTGGAAGTGGACACAGCTGGTGGTGAAACCTG  Jα22
Vα1.2-8   : TACCTGTGT  GCACTGAGCCGTCGT------------------ACTTCAACGTCCACCTGGAAAGCCACCTTTGGGAGTGGACACAGCTGGTGGTGAAACCTG              33

                                                                                   F  G  S  G
             Y  L  C                               TGGCTATGGCTCTGCATACAACAAACCAACTTTGGGTCAGGGACCAGGCTTTCTGTCCAACCAA  Jα52
Vα1.2-9   : TACCTGTGT  GCACTGGGGGTC-----------------GGGTATGGCTCTGCATACAACAAACCAACTTTGGGTCAGGGACCAGGCTTTCTGTCCAACCAA              33

                                                                                   F  G  K  G
             Y  L  C                                          TGTCCAATGCGTATGCAGTCACTTTTGGAAAAGGAACTGTGCTTTCAGTTGTACCAG  Jα27
Vα1.2-10  : TACCTGTGT  GCACTGTACCC-------------------------GTCCAATGCGTATGCAGTCACTTTTGGAAAAGGAACTGTGCTTTCAGTTGTACCAG  (*)         24


                                                                                   F  G  S  G
             Y  L  C                                          TGAATGACTTAAACAAGCTGACTTTTGGCTCAGGGACCAGGCTGTCCATCCAACCAA  Jα9
Vα1.3-1   : TACCTGTGT  GCACTGAG----------------------------GAATGACTTAAACAAGCTGACTTTTGGCTCAGGGACCAGGCTGTCCATCCAACCAA              21

                                                                                   F  G  S  G
             Y  L  C                              TGGCTCTAGCTCTTCATTCGGCAAATTGACCTTTGGCTCGGGGACCAGGCTGTCTGTCCAACCAA  Jα11
Vα1.3-2   : TACCTGTGT  GCACTGAGCC-------------------GCTCTAGCTCTTCATTCGGCAAATTGACCTTTGGCTCGGGGACCAGGCTGTCTGTCCAACCAA              30

                                                                                   F  G  S  G
             Y  L  C                                     TGACTCTGGCTATGGCAACAAGCTGACCTTTGGCTCAGGAACCAGGCTGTCCGTCCTACCAA  Jα21
Vα1.3-3   : TACCTGTGT  GCACGTC----------------------------CTGGCTATGGCAACAAGCTGACCTTTGGCTCAGGAACCAGGCTGTCCGTCCTACCAA              21

                                                                                   F  G  S  G
             Y  L  C                                     TGACTATGACTACACCAACAAACTGACCTTTGGCTCAGGGACCAGGCTGTCCGTCCTACCAA  Jα13
Vα1.3-4   : TACCTGTGT  GCACTGAGACGG----------------------TATGACTACACCAACAAACTGACCTTTGGCTCAGGGACCAGGCTGTCCGTCCTACCAA              25
Vα1.3-7   : TACCTGTGT  GCACTGGACC-------------------------ATGACTACACCAACAAACTGACCTTTGGCTCAGGGACCAGGCTGTCCGTCCTACCAA              24

                                                                                   F  G  S  G
             Y  L  C                                          TGAGTGGCAACTACAAGCTGACCTTTGGCTCAGGAACCAGGCTGTCTGTCCTACCAA  Jα17
Vα1.3-5   : TACCTGTGT  GCACTGGA----------------------------GAGTGGCAACTACAAGCTGACCTTTGGCTCAGGAACCAGGCTGTCTGTCCTACCAA              21

                                                                                   F  G  S  G
             Y  L  C                                  TGACTTTGGCTCTGGCAACAAGCTGACCTTTGGCTCAGGAACCAGGCTGTCCGTCCTACCAA  Jα35
Vα1.3-6   : TACCTGTGT  GCACTGAGCCGGGA-----------------TGACTTTGGCTCTGGCAACAAGCTGACCTTTGGCTCAGGAACCAGGCTGTCCGTCCTACCAA              33


                                                                                   F  G  S  G
             Y  L  C                                          TGAGTGGCAACTACAAGCTGACCTTTGGCTCAGGAACCAGGCTGTCTGTCCTACCAA  Jα17
Vα1.4-1   : TACCTGTGT  GCACTGAGCCGACCCCC------------------------TGGCAACTACAAGCTGACCTTTGGCTCAGGAACCAGGCTGTCTGTCCTACCAA              25

                                                                                   F  G  T  G
             Y  L  C                              TGGCTATGGCTCTGCATACGGCAGACCAACCTTTGGCACAGGGACCAGGCTGTCTGTCCAACCAA  Jα34
Vα1.4-2   : TACCTGTGT  GCACTGAGAAGTGAC-------------GGCTATGGCTCTGCATACGGCAGACCAACCTTTGGCACAGGGACCAGGCTGTCTGTCCAACCAA              36
Vα1.4-3   : TACCTGTGT  GCACTGAGCCT-------------------CTATGGCTCTGCATACGGCAGACCAACCTTTGGCACAGGGACCAGGCTGTCTGTCCAACCAA              30

                                                                                   F  G  S  G
             Y  L  C                                          TGACTAACTTATACAAGCTGACTTTTGGCTCAGGGACCAGGCTGTCTGTCCTACCAA  Jα42
Vα1.4-4   : TACCTGTGT  GCACTGATCGCGG-------------------------CTAACTTAAACAAGCTGACTTTTGGCTCAGGGACCAGGCTGTCTGTCCTACCAA              24

                                                                                   F  G  S  G
             Y  L  C                                     TGACTATGACTACACCAACAAACTGACCTTTGGCTCAGGGACCAGGCTGTCCGTCCTACCAA  Jα13
Vα1.4-5   : TACCTGTGT  GCACTGAG----------------------TGACTATGACTACACCAACAAACTGACCTTTGGCTCAGGGACCAGGCTGTCCGTCCTACCAA              27

                                                                                   F  G  S  G
             Y  L  C                                     TGATTCTGGATCCTTAAACAAGCCGACCTTTGGCTCAGGAACCAGGCTGTCCGTCCTACCCA  Jα32
Vα1.4-6   : TACCTGTGT  GCACTGGC-------------------------TTCTGGATCCTTAAACAAGCTGACCTTTGGCTCAGGAACCAGGCTGTCCGTCCTACCCA              24


                                                                                   F  G  K  G
             Y  L  C                                          TGAATGTTGGCTATACCATCATCTTTGGAAAAGGGACAAAGCTTCTTGTGAAACCAA  Jα64
Vα1.4-7   : TACCTGTGT  GCACTGAGGTG-------------------------GAATGTTGGCTATACCATCATCTTTGGAAAAGGGACAAAGCTTCTTGTGAAACCAA              24
```

```
                                                                                 F  G  S  G
            Y  L  C                               TGGCTCTGGCTCTTCATATGGCAAATTGACCTTTGGCTCAGGGACCAGGCTGTCTGTCCATCCAA  Jα41
Vα1.4-8   : TACCTGTGT  GCACTGACCCTCGCA----------------TCTAGCTCTTCATATGGCAAATTGACCTTTGGCTCAGGGACCAGGCTGTCTGTCCATCCAA         33

                                                                                 F  G  S  G
            Y  L  C                                       TGAATGACTTAAACAAGCTGACTTTTGGCTCAGGGACCAGGCTGTCCATCCAACCAA  Jα9
Vα1.4-9   : TACCTGTGT  GCACTGAGAGAGG-------------------------ATGACTTAAACAAGCTGACTTTTGGCTCGGGGACCAGGCTGTCCATCCAACCAA         24

                                                                                 F  G  S  G
            Y  L  C                                     TGACACTGGCTACAACAAGCCTACATTTGGCTCAGGGACTAGGCTGTCTGTCCTACCAA  Jα18
Vα1.4-10  : TACCTGTGT  GCACTGAGGAGGATGG----------------------CTGGCTACAACAAGCCTACATTTGGCTCAGGGACTAGGCTGTCTGTCCTACCAA         27

                                                                                 F  G  S  G
            Y  L  C                                     TGACTATGGAACCAACAGGCTGACATTTGGCTCAGGGACCAGACTGTCCGTCCAACCAA  Jα5
Vα1.4-11  : TACCTGTGT  GCACTGGCCTTATC----------------------CTATGGAACCAACAGGCTGACATTTGGCTCAGGGACCAGACTGTCCGTCCAACCAA         27


                                                                                 F  G  S  G
            Y  L  C                                        TAACTACGGTTACAAATTTACATTTGGATCTGGGACAAGACTTCTGGTTCTGCCAG  Jα67
Vα1.5     : TACCTGTGT  GCACTGGAGCGC-------------------------AACTACGGTTACAAATTTACATTTGGATCTGGGACAAGACTTCTGGTTCTGCCAG         24


                                                                                 F  G  K  G
            Y  Y  C                                       TGTATGATGACCGCACAGTCACTTTTGGAAAAGGAACTGTGCTTTCAGTTGTACCAG  Jα58
Vα2.5-1   : TATTACTGC  GCCCTGAGAACCGGAGG-------------------GTATGATGACCGCACAGTCACTTTTGGAAAAGGAACTGTGCTTTCAGTTGTACCAG         30

                                                                                 F  G  A  G
            Y  Y  C                                    CCAATCAGGGATATGTGAAACTGGCATTTGGAGCTGGCACCCAACTTCTTGTTATCCCAA  Jα68
Vα2.5-2   : TATTACTGC  GCCCTGAGAGCTGC-------------------------GGGATATGTGAAACTGGCATTTGGAGCTGGCACCCAACTTCTTGTTATCCCAA         24

                                                                                 F  G  K  G
            Y  Y  C                                       TGAATGTTGGCTATACCATCATCTTTGGAAAAGGGACAAAGCTTCTTGTGAAACCAA  Jα64
Vα2.5-3   : TATTACTGC  GCCCTGCCGGGGCC-------------------------------CTATACCATCATCTTTGGAAAAGGGACAAAGCTTCTTGTGAAACCAA         18

                                                                                 F  G  S  G
            Y  Y  C                               TGGCTATGGCTCTGGATATGACAGACTGACTTTTGGCTCAGGAACCAGGCTGTCTGTCCAACCAA  Jα46
Vα2.5-4   : TATTACTGC  GCCCCCCT----------------------CTATGGCTCTGGATATGACAGACTGACTTTTGGCTCAGGAACCAGGCTGTCTGTCCAACCAA         27

                                                                                 F  G  S  G
            Y  Y  C                                  TGACTTTGGCTCTGGCAACAAGCTGACCTTTGGCTCAGGAACCAGGCTGTCCGTCCTACCAA  Jα35
Vα2.5-5   : TATTACTGC  GCCCTGCTCCCGA----------------------TTGGCTCTGGCAACAAGCTGACCTTTGGCTCAGGAACCAGGCTGTCCGTCCTACCAA         27

                                                                                 F  G  S  G
            Y  Y  C                                       TGTTTGGCTACAGCCAGCTGACTTTTGGCTCAGGGACCAGGCTGTCCGTCCAACCAA  Jα61
Vα2.5-6   : TATTACTGC  GCCCACTACGCGG-------------------------TTGGCTACAGCCAGCTGACTTTTGGCTCAGGGACCAGGCTGTCCGTCCAACCAA         24

                                                                                 F  G  S  G
            Y  Y  C                               TGGCTCTAGCTCTTCATTCGGCAAATTGACCTTTGGCTCGGGGACCAGGCTGTCTGTCCAACCAA  Jα11
Vα2.5-7   : TATTACTGC  GCCCTGAGAGCCCCGCGGGCGGA----TGGCTCTAGCTCTTCATTCGGCAAATTGACCTTTGGCTCGGGGACCAGGCTGTCTGTCCAACCAA         45

                                                                                 F  G  S  G
            Y  Y  C                                  TGACTATGACTACACCAACAAACTGACCTTTGGCTCAGGGACCAGGCTGTCCGTCCTACCAA  Jα13
Vα2.5-8   : TATTACTGC  GCCCTGAGAGCGCG---------------------ATGACTACACCAACAAACTGACCTTTGGCTCAGGGACCAGGCTGTCCGTCCTACCAA  (*)  28

                                                                                 F  G  S  G
            Y  Y  C                                     TGACTATGGGAACTACAAACTGACCTTTGGCTCAGGGACCAGGCTGTCCGTCCAACCAA  Jα60
Vα2.5-9   : TATTACTGC  GCCCTGTGGCTCGTGG----------------------ATGGGAACTACAAACTGACCTTTGGCTCAGGGACCAGGCTGTCCGTCCAACCAA         27


                                                                                 F  G  S  G
            Y  Y  C                                  TGATTCTGGATCCTTAAACAAGCCGACCTTTGGCTCAGGAACCAGGCTGTCCGTCCTACCCA  Jα32
Vα2.6-1   : TATTACTGC  GCCCTGAGAGCCGCGGG------------------CTGGATCCTTAAACAAGCCGACCTTTGGCTCAGGAACCAGGCTGTCCGTCCTACCCA  (*)  31

                                                                                 F  G  K  G
            Y  Y  C                                       TGTATGATGACCGCACAGTCACTTTTGGAAAAGGAACTGTGCTTTCAGTTGTACCAG  Jα58
Vα2.6-2   : TATTACTGC  GCCCTGAACGCGGAGG----------------------ATGATGACCGCACAGTCACTTTTGGAAAAGGAACTGTGCTTTCAGTTGTACCAG         27

                                                                                 F  G  S  G
            Y  Y  C                               TGGCTATGGCTCTGGATATGACAGACTGACTTTTGGCTCAGGAACCAGGCTGTCTGTCCAACCAA  Jα46
Vα2.6-3   : TATTACTGC  GCCCC-------------------------CTATGGCTCTGGATATGACAGACTGACTTTTGGCTCAGGAACCAGGCCGTCTGTCCAACCAA         24

                                                                                 F  G  S  G
            Y  Y  C                                  TGACTTTGGCTCTGGCAACAAGCTGACCTTTGGCTCAGGAACCAGGCTGTCCGTCCTACCAA  Jα35
Vα2.6-4   : TATTACTGC  GCCCTGAGACGACGCCC----------------CTTTGGCTCTGGCAACAAGCTGACCTTTGGCTCAGGAACCAGGCTGTCCGTCCTACCAA         33


                                                                                 F  G  K  G
            Y  Y  C                                       TGAATGTTGGCTATACCATCATCTTTGGAAAAGGGACAAAGCTTCTTGTGAAACCAA  Jα64
Vα2.7     : TATTACTGC  GCCCTGAGGGACG-------------------------ATGTTGGCTATACCATCATCTTTGGAAAAGGGACAAAGCTTCTTGTGAAACCAA         24


                                                                                 F  G  E  G
            Y  Y  C                               TAACTCCATACGTGCCTATGAAACACTCAGTTTTGGAGAGGGGACAAGGCTGACTGTTCTTCCAA  Jα1
Vα2.8     : TATTACTGC  GCCCTGATGAGATT---------------------TACGTGCCTATGAAACACTCAGTTTTGGAGAGGGGACAAGGCTGACTGTTCCTCCAA  (*)  28


                                                                                 F  G  K  G
            Y  Y  C                                       TGAATGTTGGCTATACCATCATCTTTGGAAAAGGGACAAAGCTTCTTGTGAAACCAA  Jα64
Vα2.9-1   : TATTACTGC  GCCTGGGG------------------------------ATGTTGGCTATACCATCATCTTTGGAAGAGGGACAAAGCTTCTTGTGAAACCAA  (*)  19

                                                                                 F  G  A  G
            Y  Y  C                                    CCAATCAGGGATATGTGAAACTGGCATTTGGAGCTGGCACCCAACTTCTTGTTATCCCAA  Jα68
Vα2.9-2   : TATTACTGC  GCCCTGAGAGCGCCAGTACGC------------------GGGATATGTGAAACTGGCATTTGGAGCTGGCACCCAACTTCTTGTTATCCCAA  (*)  31
```

```
                                                                                F  G  T  G
           Y  Y  C                               TGGCTATGGCTCTGCATACGGCAGACCAACCTTTGGCACAGGAACCAGGCTTTCTGTCCAACCAA  Jα20
Vα2.9-3  : TATTACTGC  GCCCCCTATC-------------------GTTATGGCTCTGCATACGGCAGACCAACCTTTGGCACAGGAACCAGGCTTTCTGTCCAACCAA            30


                                                                                F  G  S  G
           Y  Y  C                                       TGTTTGGCTACAGCCAGCTGACTTTTGGCTCAGGGACCAGGCTGTCCGTCCAACCAA  Jα61
Vα2.10-1 : TATTACTGC  GCCCTGAGAGCCCTCTG-------------------GTTTGGCTACAGCCAGCTGACTTTTGGCTCAGGGACCAGGCTGTCCGTCCAACCAA            30

                                                                                F  G  S  G
           Y  L  C                                        TAACTACGGTTACAAATTTACATTTGGATCTGGGACAAGACTTCTGGTTCTGCCAG  Jα67
Vα2.10-2 : TATTACTGC  GCCCTGAGAGCTAACTACGGAGGT-------------------GGTTACAAATTTACATTCGGTTCTGGGACAAGACTTCTGGTTCTGCCAG            30


                                                                                F  G  S  G
           Y  Y  C                                     CGACTATGGGAACAACAAACTGACCTTTGGCTCAGGGACCAGGCTGTCTGTCCAACCAA  Jα57
Vα2.11   : TATTACTGC  GCCCTGAGTGCCCCG----------------------TATGGGAACAACAAACTGACCTTTGGCTCAGGGACCAGGCTGTCTGTCCAACCAA            27


                                                                                F  G  S  G
           Y  Y  C                                     TGACACTGGCTACAACAAGCCTACATTTGGCTCAGGGACTAGGCTGTCTGTCCTACCAA  Jα18
Vα2.12-1 : TATTACTGC  GCCCTGGATTGGG-------------------------CTGGCTACAACAAGCCTACATTTGGCTCAGGGACTAGGCTGTCTGTCCTACCAA            24

                                                                                F  G  S  G
           Y  Y  C                               TGGCTCTGGCTCTTCATATGGCAAATTGACCTTTGGCTCAGGGACCAGGCTGTCTGTCCATCCAA  Jα41
Vα2.12-2 : TATTACTGC  GCCCTGAG-------------------------TGGCTCTTCATATGGCAAATTGACCTTTGGCTCAGGGACCAGGCTGTCTGTCCAACCAA            24

                                                                                F  G  S  G
           Y  Y  C                                     TGAATCTGGCTTCACCAAACTGACCTTTGGCTCAGGGACCCGGCTGTCCGTCCTACCAA  Jα23
Vα2.12-3 : TATTACTGC  GCTCTCGTACGG----------------------GAATCTGGCTTCACCAAACTGACCTTTGGCTCAGGAACCCGGCTGTCCGTCCTACCAA            27


                                                                                F  G  S  G
           Y  Y  C                                        TAACTACGGTTACAAATTTACATTTGGATCTGGGACAAGACTTCTGGTTCTGCCAG  Jα67
Vα2.13-1 : TATTACTGC  GCCCTGAGAGCGGCTTCATGGCGGGGGA--------TAACTACGGTTACAAATTTACATTTGGATCTGGGACAAGACTTCTGGTTCTGCCAG  (*)       41

                                                                                F  G  S  G
           Y  Y  C                               TGGCTATGGCTCTGGATATGACAGACTGACTTTTGGCTCAGGAACCAGGCTGTCTGTCCAACCAA  Jα46
Vα2.13-2 : TATTACTGC  GCCCTGAGAGCGCTA-------------------GGCTCTGGATATGACAGACTGACTTTTGGCTCAGGAACCAGGCTGTCTGTCCAACCAA            30

                                                                                F  G  T  G
           Y  Y  C                               TGGCTATGGCTCTGCATACGGCAGACCAACCTTTGGCACAGGAACCAGGCTTTCTGTCCAACCAA  Jα20
Vα2.13-3 : TATTACTGC  GCCCTGAGGCGG-------------------TATGGCTCTGCATACGGCAGACCAACCTTTGGCACAGGAACCAGGCTTTCTGTCCGACCAA            30


                                                                                F  G  S  G
           Y  Y  C                                     TGACTATGGAACCAACAGGCTGACATTTGGCTCAGGGACCAGACTGTCCGTCCAACCAA  Jα5
Vα2.14   : TATTACTGC  GCCCCGCAC---------------------------CTATGGAACCAACAGGCTGACATTTGGCTCAGGGACCAGACTGTCCGTCCAACCAA  (*)       22

                                                                                F  G  S  G
           Y  Y  C                               TGGCTATGGCTCTGGATATGGCAAACTGACCTTTGGCTCAGGGACCAGGCTGTCTGTCCAACCAA  Jα63
Vα2.15   : TATTACTGC  GCCCTGA----------------------GCTATGGCTCTGGATATGGCAAACTGACCTTTGGCTCAGGGACCAGGCTGTCTGTCCAACCAA            27


                                                                                W  G  S  G
           Y  Y  C                                 CTGATTCTGGAGGATGGGGAAAATATACATGGGGGAGTGGAACAAAACTTCTTGTTGCACCAG  Jα65
Vα2.16-1 : TATTACTGC  GCCCTGATGCTGGCG-------------------TCTGGAGGATGGGGAAAATATACATGGGGGAGTGGAACAAAACTTCTCGTTGCACCAG            30


                                                                                F  G  A  G
           Y  L  C                                    CCAATCAGGGATATGTGAAACTGGCATTTGGAGCTGGCACCCAACTTCTTGTTATCCCAA  Jα68
Vα3.5-1  : TACCTCTGT  GCTGCCCGGGCGCTC----------------------CAGGGATATGTGAAACTGGCATTTGGAGCTGGCACCCAACTTCTTGTTATCCCAA            27
Vα3.5-4  : TACCTCTGT  GCTGTCA-------------------------------AGGGATATGTGAAACTGGCATTTGGAGCTGGCAGCCAACTTCTTGTTATCCCAA            18

                                                                                F  G  S  G
           Y  L  C                               TGGCTATGGCTCTGGATATGGCAAACTGACCTTTGGCTCAGGGACCAGGCTGTCTGTCCAACCAA  Jα63
Vα3.5-2  : TACCTCTGT  GTGGCGGTCCCGAGCAC-------------CTATGGCTCTGGATATGGCAAACTGACCTTTGGCTCAGGGACCAGGCTGTCTGTCCAACCAA            36
Vα3.5-6  : TACCTCTGT  GCTGTGAGGTC-------------------CTATGGCTCTGGATATGGCAAACTGACCTTTGGCTCAGGGACCAGGCTGTCTGTCCAACCAA            30
                                                                                F  G  S  G
           Y  L  C                                  TGACTTTGGCTCTGGCAACAAGCTGACCTTTGGCTCAGGAACCAGGCTGTCCGTCCTACCAA  Jα35
Vα3.5-3  : TACCTCTGT  GCTGTGAGACTCCCGGGAG----------------TTGGCTCTGGCAACAAGCTGACCTTTGGCTCAGGAACCAGGCTGTCCGTCCTACCAA            33

                                                                                F  G  K  G
           Y  L  C                                       TGAATGTTGGCTATACCATCATCTTTGGAAAAGGGACAAAGCTTCTTGTGAAACCAA  Jα64
Vα3.5-5  : TACCTCTGT  GCTGTGAGAG----------------------------ATGCTGGCTATACCATCATCTTTGGAAAAGGGACAAAGCTTCTTGTGAAACCAA            21

                                                                                F  G  S  G
           Y  L  C                                    TGACTGGCAACAACAACAAGCTGACCTTTGGCTCAGGAACCAGGCTGTCCGTCCTACCCA  Jα50
Vα3.5-7  : TACCTCTGT  GCTCTCCGAT----------------------TGACTGGCAACAACAACAAGCTGACCTTTGGCTCAGGAACCAGGCTGTCCGTCCTACCCA            27


                                                                                F  G  K  G
           Y  L  C                                       TGTCCAATGCGTATGCAGTCACTTTTGGAAAAGGAACTGTACTTTCAGTTGTACCAG  Jα40
Vα3.5-8  : TACCTCTGT  GCTGTGAGGCCCGCGA-------------------TGTCCAATGCGTATGCAGTCACTTTTGGAAAAGGAACTGTACTTTCAGTTGTACCAG            30


                                                                                F  G  S  G
           Y  L  C                               TGGCTCTAGCTCTGGATATGGCAAACTAAACTTTGGCTCTGGAACCAGGCTGTCTGTCCATCCAA  Jα59
Vα3.6    : TACCTCTGT  GCTGTGAGTGCCCGGCTAGGAGACGC----CTCTAGCTCTGGATATGGCAAACTAAACTTTGGCTCTGGAACCAGGCTGTCTGTCCATCCAA            45
```

```
                                                                                F  G  S  G
           Y  L  C                               TGGCTATGGCTCTGCATACAACAAACCAACTTTTGGGTCAGGGACCAGGCTTTCTGTCCAACCAA  Jα52
Vα3.7-1  : TACCTCTGT  GCTGTGAGTACAGA-------------TGGCTATGGCTCTGCATACAACAAACCAACTTTTGGGTCAGGGACCAGGCTTTCTGTCCAACCAA            36

                                                                                F  G  S  G
           Y  L  C                                       TGAATGACTTAAACAAGCTGACTTTTGGCTCAGGGACCAGGCTGTCCATCCAACCAA  Jα9
Vα3.7-2  : TACCTCTGT  GCTGTGAGTCGACCGGCCCC-----------------AATGACTTAAACAAGCTGACTTTTGGCTCGGGGACCAGGCTGTCCATCCAACCAA  (*)       32

                                                                                F  G  S  G
           Y  L  C                               TGGCTATGGCTCTGGATATGGCAAACTGACCTTTGGCTCAGGGACCAGGCTGTCTGTCCAACCAA  Jα63
Vα3.7-3  : TACCTCTGT  GCTGTGAGTGCGCCCC-------------GCTATGGCTCTGGATATGGCAAACTGACCTTTGGCTCAGGGACCAGGCTGTCTGTCCAACCAA            36

                                                                                F  G  S  G
           Y  L  C                                  TGACTTTGGCTCTGGCAACAAGCTGACCTTTGGCTCAGGAACCAGGCTGTCCGTCCTACCAA  Jα35
Vα3.7-4  : TACCTCTGT  GCTG-------------------------------TTGGCTCTGGCAACAAGCTGACCTTTGGCTCAGGAACCAGGCTGTCCGTCCTACCAA            18

                                                                                F  G  S  G
           Y  L  C                                  TGATTCTGGATCCTACAACAAGCTGACCTTTGGCTCAGGAACCAGGCTGTCTGTCAAACCAA  Jα43
Vα3.7-5  : TACCTCTGT  GCTGTGAGTGATCCCCGGGGCCGGGTC-------TCTGGATCCTACAACAAGGTGACCTTTGGCTCAGGAACCAGGCTGACTGTCAAACCAA            42

                                                                                F  G  K  G
           Y  L  C                                          TGAACTACGACAAGTTCACATTTGGGAAGGGAACACGACTTCAGGTTTTGCCTA  Jα16
Vα3.7-6  : TACCTCTGT  GCTGTGACCCCTC-------------------------TGAACTACGACGAGCTCACATTTGGGAAGGGAACACGACTTCAGGTTTTGCCTA            24

                                                                                F  G  K  G
           Y  L  C                                       TGAATGTTGGCTATACCATCATCTTTGGAAAAGGGACAAAGCTTCTTGTGAAACCAA  Jα64
Vα3.7-7  : TACCTCTGT  GCTGTGAGTGACGATG----------------------ATGTTGGCTATACCATCATCTTTGGAAAAGGGACAAAGCTTCTTGTGACACCAA            27

                                                                                F  G  K  G
           Y  L  C                                       TGTCCAATGCGTATGCAGTCACTTTTGGAAAAGGAACTGTGCTTTCAGTTGTACCAG  Jα27
Vα3.7-8  : TACCTCCGT  GCTGTGAGTGATCCCCGGC----------------------------ATGCAGTCACTTTTGGAAAAGGAACTGTGCTTTCAGTTGTACCAG            21


                                                                                F  G  S  G
           F  F  C                                     TGAATCTGGCTTCACCAAACTGACCTTTGGCTCAGGGACCCGGCTGTCCGTCCTACCAA  Jα23
Vα3.8    : TTCTTCTGT  GCTGTGAGTGCGGG--------------------GAATCTGGCTTCACCAAACTGACCTTTGGCTCAGGGACCCGGCTGTCCGTCCTACCAA  (*)       29


                                                                                F  G  S  G
           F  F  C                               TGGCTCTGGCTCTTCATATGGCAAATTGACCTTTGGCTCAGGGACCAGGCTGTCTGTCCATCCAA  Jα41
Vα3.9-1  : TTCTTCTGT  GCTGTGACGATGC-------------------CTGGCTCTTCATATGGCAAATTGACCTTTGGCTCAGGGACCAGGCTGTCTGTCCTACCAA            30
Vα3.9-7  : TTCTTCTGT  GGTGTGAGTGTACCCGGCGAC-------GGCTCTGGCTCTTCATATGGCAAATTGACCTTTGGCTCAGGGACCAGGCTGTCTGTCCATCCAA            42

                                                                                F  G  S  G
           F  F  C                                  TGATTTTGGATCCTTCAACAAGCTGATCTTTGGCTCAGGAACCAGGCTGTCCGTCCTACCCA  Jα19
Vα3.9-2  : TACCTCTGT  GCCCGTATGACGC----------------------TTGGATCCTTCAACAAGCTGATCTTTGGCTCAGGAACCAGGCTGTCCGTCCTACCCA            27

                                                                                F  G  S  G
           F  F  C                                     TGACTATGGAACCAACAGGCTGACATTTGGCTCAGGGACCAGACTGTCCGTCCAACCAA  Jα5
Vα3.9-3  : TTCTTCTGT  GCTGTGG-------------------------------ATGGAACCAACAGGCTGACATTTGGCTCAGGGACCAGACTGTCCGTCCAACCAA            18

                                                                                F  G  S  G
           F  F  C                                     TGACACTGGCTACAACAAGCTGACCTTTGGCTCAGGGACTAGGCTGTCTGTCCTACCAA  Jα49
Vα3.9-4  : TTCTTCTGT  GCTGTGAGAGA----------------------TGACACTGGCTACAACAAGCTGACCTTTGGCTCAGGGACTAGGCTGTCTGTCCTACCAA            27

                                                                                F  G  S  G
           F  F  C                                       TGAATGACTTAAACAAGCTGACTTTTGGCTCAGGGACCAGGCTGTCCATCCAACCAA  Jα9
Vα3.9-5  : TTCTTCTGT  GCTGTGACGGGCG-------------------------ATGACTTAAACAAGCTGACTTTTGGCTCAGGGACCAGGCTGTCCATCCAACCAA            24
Vα3.9-6  : TACTTCTGT  GCTG-------------------------------TGAATAACTTAAACAAGCTGACTTTTGGCTCAGGGACCAGGCTGTCCGTCCTACCAA            18

                                                                                F  G  T  G
           F  F  C                               TGGCTATGGCTCTGCATACGGCAGACCAACCTTTGGCACAGGGACCAGGCTGTCTGTCCAACCAA  Jα34
Vα3.9-8  : TTCTTCTGT  GCTGTGAGTGGAGA-------------TGGCTATGGCTCTGCATACGGCAGACCAACCTTTGGCACAGGGACCAGGCTGTCTGTCCAACCAA            36

                                                                                F  G  S  G
           F  F  C                                       TGAGTGGCAACTACAAGCTGACCTTTGGCTCAGGAACCAGGCTGTCTGTCCTACCAA  Jα17
Vα3.9-9  : TTCTTCTGT  GCTGTGGCCCCC------------------------GAGTGGCAACTACAAGCTGACCTTTGGCTCAGGAACCAGGCTGTCTGTCCCACCAA  (*)       25
                                                                                F  G  S  G
           F  F  C                                  TGACTCTGGCTATGGCAACAAGCTGACCTTTGGCTCAGGAACCAGGCTGTCCGTCCTACCAA  Jα21
Vα3.9-10 : TTCTTCTGT  GCTGTGCGTCGTACACGA----------------TCTGGCTATGGCAACAAGCTGACCTTTGGCTCAGGAACCAGGCTGTCCGTCCTACCAA            33

                                                                                W  G  S  G
           F  F  C                                 CTGATTCTGGAGGATGGGGAAAATATACATGGGGGAGTGGAACAAAACTTCTTGTTGCACCAG  Jα65
Vα3.9-11 : TTCTTCTGT  GCTGTGAGTG--------------------TGATTCTGGAGGATGGGGAAAATATACATGGAGGAGTGGAACAAAACTTCTTGTTGCACCAG  (*)       29


                                                                                F  G  S  G
           Y  F  C                                  TGACTCTGGCTATGGCAACAAGCTGACCTTTGGCTCAGGAACCAGGCTGTCCGTCCTACCAA  Jα21
Vα3.10-1 : TACTTCTGT  GCTGTGAGTGACGGAACG-------------GACTCTGGCTATGGCAACAAGCTGACCTTTGGCTCAGGAACCAGGCTGTCCGTCCTACCAA            36


                                                                                F  G  S  G
           Y  F  C                                       TGACTAACTTATACAAGCTGACTTTTGGCTCAGGGACCAGGCTGTCTGTCCTACCAA  Jα42
Vα3.10-2 : TACTTCTGT  GCTGTGGGGGGA-----------------------TGACTAACTTAAACAAGCTGACTTTTGGCTCAGGGACCAGGCTGTCTGTCCTACCAA            27

                                                                                F  G  S  G
           Y  F  C                                        TAACTACGGTTACAAATTTACATTTGGATCTGGGACAAGACTTCTGGTTCTGCCAG  Jα67
Vα3.10-3 : TACTTCTGT  GCTGTGGGAGATAACTACGGAGGT-------------------GGTTACAAATTTACATTCGGCTCTGGGACAAGACTTCTGGTTCTGCCAG  (*)       30

                                                                                F  G  A  G
           Y  F  C                                    CCAATCAGGGATATGTGAAACTGGCATTTGGAGCTGGCACCCAACTTCTTGTTATCCCAA  Jα68
Vα3.10-4 : TACTTCTGT  GCTGTGAGGATCCCCGTGG-------------CCAATCAGGGATATGTGAAACTGGCATTTGGAGCTGGCACCCAACTTCTTGTTATCCCAA            36

                                                                                F  G  S  G
           Y  F  C                                       TGAATGACTTAAACAAGCTGACTTTTGGCTCAGGGACCAGGCTGTCCATCCAACCAA  Jα9
Vα3.10-5 : TACTTCTGT  GCTGTGCGAATCCCCCAGA----------------TGAATGACTTAAACAAGCTGACCTTTGGCTCAGGGACCAGGCTGTCCGTCCAACCAA  (*)       33
```

```
                                                                       F  G  S  G
           Y  F  C                                    TGAATGACTTAAACAAGCTGACTTTTGGCTCAGGGACCAGGCTGTCCATCCAACCAA  Jα9
Vα3.11   : TACCTCTGT  GCTGTGCGTGAAGAGACGCCCCT-------------------CTTAAACAAGCTGACTTTTGGCTCAGGGACCAGGCTGTCCATCCAACCAA  (*)      30


                                                                       F  G  S  G
           Y  L  C                                  CGACTATGGAACAACAAACTGACCTTTGGCTCAGGGACCAGGCTGTCTGTCCAACCAA  Jα57
Vα3.12-1 : TACCTCTGT  GCTGTGCGTGGGGGA-------------------GACTATGGAACAACAAACTGACTTTTGGCTCAGGGACCAGGCTGTCTGTCCAACCAA           30
Vα3.12-8 : TACCTCTGT  GCTGTGCGCCG----------------------CGACTATGGAACAACAAACTGACCTTTGGCTCAGGGACCAGGCTGTCTGTCCTACCAA           27

                                                                       F  G  S  G
           Y  L  C                                     TAACTACGGTTACAAATTTACATTTGGATCTGGGACAAGACTTCTGGTTCTGCCAG  Jα67
Vα3.12-2 : TACCTCTGT  GCTGTGCGTGAGACCCGCGGAGGT-------------------GGTTACAAATTTACATTCGGCTCTGGGACAAGACTTCTGGTTCTGCCAG           30

                                                                       F  G  S  G
           Y  L  C                          TGGCACTGGCTCTACATACGGCAAATTGACCTTTGGCTCAGGGACCAGGCTGTCTGTCCAACCAA  Jα15
Vα3.12-3 : TACCTCTGT  GCTGTGCGTGATGACAGG----------GGCTCTGGCTCTACATACGGCAAATTGACCTTTGGCTCAGGAACCAGGCTGTCCGTCCATCCAA           39

                                                                       F  G  S  G
           Y  L  C                             TGACTTTGGCTCTGGCAACAAGCTGACCTTTGGCTCAGGAACCAGGCTGTCCGTCCTACCAA  Jα35
Vα3.12-4 : TACCTCTGT  GCTGTGCGTGCGCCGCC----------------CTTTGGCTCTGGCAACAAGCTGACCTTTGGCTCAGGAACCAGGCTGTCCGTCCTACCAA           33

                                                                       F  G  D  G
           Y  L  C                                TGTCTTTGGATACCTACAAGCTAACCTTTGGAGATGGAACAAGGCTCATGGTAAAACCAA  Jα66
Vα3.12-5 : TACCTCTGT  GCTGTGCGTGGGA-------------------TGTCTCTGGATACCTACAAGCTAACCTTTGGAGATGGAACAAGGCTCATGGTAAAACCAA           30

                                                                       F  G  K  G
           Y  L  C                                    TGAATGTTGGCTATACCATCATCTTTGGAAAAGGGACAAAGCTTCTTGTGAAACCAA  Jα64
Vα3.12-6 : TACCTCTGT  GCTGTGCGTGACCC----------------------GAATGCTGGCTATACCATCATCTTTGGAAAAGGGACAAAGCTTCTTGTGAAACCAA           27

                                                                       L  G  S  G
           Y  L  C                                       TGAACTACGGCAAGATCACCCTTGGGAGCGGAACACGACTTCAGGTTTTGCCTA  Jα12
Vα3.12-7 : TACCTCTGT  GCCGC-------------------------------------CTACGGCAAGATCACCCTTGGGAGCGGAACACGACTTCAGGTTTTGCCTA           12

                                                                       F  G  S  G
           Y  L  C                             TGATTCTGGATCCTACAACAAGCTGACCTTTGGCTCAGGAACCAGGCTGTCTGTCAAACCAA  Jα43
Vα3.12-9 : TACCTCTGT  GCTGTGCGTGACCGCCCGGCGTGGGTCGA–TGATTCTGGATCCTACAACAAGCTGACCTTTGGCTCAGGAACCAGGCTGTCTGTCAAACCAA           48

                                                                       F  G  S  G
           Y  L  C                                  TGACTACAGCGGCGACAAGCTGACCTTTGGCTCAGGGACCAGGCTGTCTGTCCTACCAA  Jα7
Vα3.12-10: TACCTCTGT  GCTGTGCGTGCTAACTTAT----------------------------ACAAGCTGACTTTTGGCTCAGGGACCAGGCTGTCTGTCCTACCAA           21

                                                                       F  G  S  G
           Y  L  C                             TGACTATGACTACACCAACAAACTGACCTTTGGCTCAGGGACCAGGCTGTCCGTCCTACCAA  Jα13
Vα3.12-11: TACCTCTGT  GCTGTGCGTGATGCCGC----------------CTATGACTACACCAACAAACTGACCTTTGGCTCAGGGACCAGGCTGTCCGTCCTACCAA           33


                                                                       F  G  A  G
           Y  F  C                                CCAATCAGGGATATGTGAAACTGGCATTTGGAGCTGGCACCCAACTTCTTGTTATCCCAA  Jα68
Vα3.13-1 : TACTTCTGT  GCTGTGAGAGG---------------------CCAATCAGGGATATGTGAAACTGGCATTTGGAGCTGGCACCCAACTTCTTGTTATCCCAA  (*)      28

                                                                       F  G  D  G
           Y  F  C                                TGTCTTTGGATACCTACAAGCTAACCTTTGGAGATGGAACAAGGCTCATGGTAAAACCAA  Jα66
Vα3.13-2 : TACTTCTGT  GCTGTGGTTC----------------------------TGGATACCTACAAGCTAACCTTTGGAGATGGAACAAGGCTCATGGTAAAACCAA           21


                                                                       F  G  K  G
           Y  F  C                                    AGTTAAATGCGCGCACAGTCACTTTTGGGAAAGGAACTGTGCTTTCAGTTGTTCCAG  Jα14
Vα3.14-1 : TACTTCTGT  GCTGTGAGTGGACGGGACC----------------------ATGCGCGCACAGTCACTTTTGGGAAAGGAACTGTGCTTTCAGTTGTTCCAG           27

                                                                       F  G  S  G
           Y  F  C                          TGGCTCTGGCTCTTCATATGGCAAATTGACCTTTGGCTCAGGGACCAGGCTGTCTGTCCATCCAA  Jα41
Vα3.14-2 : TACTTCTGT  GCTGTGAGCCATCGTCA----------TGGCTCTGGCTCTTCATATGGCAAATTGACCTTTGGCTCAGGGACCAGGCTGTCTGTCCATCCAA           39

                                                                       F  G  S  G
           Y  F  C                                  TGACACTGGCTACAACAAGCTGACCTTTGGCTCAGGGACTAGGCTGTCTGTCCTACCAA  Jα49
Vα3.14-3 : TACTTCTGT  GCTGTGTCCC-------------------------ACACTGGCTACAACAAGCTGACCTTTGGCTCAGGGACTAGGCTGTCTGTCCTACCAA           24

                                                                       F  G  S  G
           Y  F  C                          TGGCTATGGCTCTGGATATGACAGAATGACTTTTGGCTCAGGAACCAGGCTGTCTGTCCAACCAA  Jα28
Vα3.14-4 : TACTTCTGT  GCTGTAGACCT-------------------CTATGGCTCTGGATATGACAGAATGACTTTTGGCTCAGGAACCAGGCTGTCTGTCCAACCAA           30

                                                                       F  G  S  G
           Y  L  C                             TGACTTTGGCTCTGGCAACAAGCTGACCTTTGGCTCAGGAACCAGGCTGTCCGTCCTACCAA  Jα35
Vα3.14-5 : TACTTCTGT  GCTGTGATGACCCCCACTA----------------TTGGCTCTGGCAACAAGCTGACCTTTGGCTCAGGAACCAGGCTGTCCGTCCTACCAA           33
Vα3.14-6 : TACTTCTGT  GCTGTGAGGCGTTGGAGAGGAC----------------GCTCTGGCAACAAGCTGACCTTTGGCTCAGGAACCAGGCTGTCCGTCCTACCAA           33
                                                                       F  G  S  G
           Y  L  C                             TGACTATGACTACACCAACAAACTGACCTTTGGCTCAGGGACCAGGCTGTCCGTCCTACCAA  Jα13
Vα3.15   : TACCTCTGT  GCTGTGAAGGA-------------------TGACTATGACTACACCAACAAACTGACCTTTGGCTCAGGGACCAGGCTGTCCGTCCTACCAA           30


                                                                       F  G  S  G
           Y  L  C                          TGGCTCTAGCTCTTCATTCGGCAAATTGACCTTTGGCTCGGGGACCAGGCTGTCTGTCCAACCAA  Jα11
Vα3.16-1 : TACCTCTGT  GCTGTGCC----------------------CTCTAGCTCTTCATTCGGCAAATTGACCTTTGGCTCGGGGACCAGGCTGTCTGTCCAACCAA           27

                                                                       F  G  S  G
           Y  L  C                                  TGACTATGGAACCAACAGGCTGACATTTGGCTCAGGGACCAGACTGTCCGTCCAACCAA  Jα5
Vα3.16-2 : TACCTCTGT  GCTGTGCGTGAAGA-------------------TGACTATGGAACCAACAAGCTGACATTTGGCTCAGGGACCAGACTGTTCGTCCAACCAA  (*)      27

                                                                       F  G  S  G
           Y  L  C                                TGACTGGCAACAACAACAAGCTGACCTTTGGCTCAGGAACCAGGCTGTCCGTCCTACCCA  Jα50
Vα3.16-3 : TACCTCTGT  GCTGTGCGTGATACGG-------------------------------ACAAGCTGACCTTTGGCTCAGGAACCAGGCTGTCCGTCCTACCCA           18


                                                                       F  G  S  G
           Y  L  C                          TGGCTATGGCTCTGGATATGACAGACTGACTTTTGGCTCAGGAACCAGGCTGTCTGTCCAACCAA  Jα46
Vα3.17-1 : TACCTCTGT  GCTGTGCGTGACGGG-------------GGCTATGGCTCTGGATATGACAGACTGACTTTTGGCTCAGGAACCAGGCTGTCTGTCCAACCAA           36
```

```
                                                                                 F  G  S  G
            Y  L  C                                    TCACTACAGCAGTGACAAGCTGACCTTTGGCTCAGGGACCAGGCTGTCCGTCCTACCAA  Jα24
Vα3.17-2 : TACCTCTGT  GCTGTGCGGG----------------------------ACAGCAGTGACAAGCTGACCTTTGGCTCAGGGACCAGGCTGTCCGTCCTACCAA                21


                                                                                 F  G  K  G
            Y  L  C                                      TATACAATGCGGGCAAAGTCACTTTTGGAAAAGGAACTGTGCTTTCAGTTGTACCAG  Jα56
Vα3.18   : TACCTCTGT  GCTGTAGACACGC----------------------TATACAATGCGGGCAAAGTCACTTTTGGGAAAGGAACTGTGCTTTCAGTTGTACCAG                27


                                                                                 F  G  S  G
            Y  F  C                                    CGACTATGGGAACAACAAACTGACCTTTGGCTCAGGGACCAGGCTGTCTGTCCAACCAA  Jα57
Vα3.19   : TACTTCTGT  GCTGTGAGCGAGGGTGG----------------CGACTATGGGAACAACAAACTGACCTTTGGCTCAGGGACCAGGCTGTCTGTCCAACCAA                33


                                                                                 F  G  A  G
            Y  F  C                                   CCAATCAGGGATATGTGAAACTGGCATTTGGAGCTGGCACCCAACTTCTTGTTATCCCAA  Jα68
Vα3.20-1 : TACTTCTGT  GCTGTGAGGGG-------------------------TCAGGGATATGTGAAGCTGGCATTTGGAGCTGGCAGCCAACTTCTTGTTATCCCAA                24

                                                                                 F  G  S  G
            Y  F  C                                    TGACTACAGCGGTGACAAGCTGACCTTTGGCTCAGGGACCAGGCTGTCTGTCCTACCAA  Jα25
Vα3.20-2 : TACTTCTGT  GCTGTGAGTGGCCCGTT-------------------CTACAGCCGTGACAAGCTGACCTTTGGCTCAGGGACCAGGCTGTCTGTCCTACCAA                30
Vα3.20-3 : TACTTCTGT  GCTGTCCAGT--------------------------CTACAGCCGTGACAAGCTGACCTTTGGCTCAGGGACCAGGCTGTCTGTCCTACCAA  (*)           23

                                                                                 F  G  S  G
            Y  F  C                                      TGAGTGGCAACTACAAGCTGACCTTTGGCTCAGGAACCAGGCTGTCTGTCCTACCAA  Jα17
Vα3.20-4 : TACTTCTGT  GCTGTGAAGGGGGGTGCGAC-------------------TGGCAACTACAAGCTGACCTTTGGCTCAGGAACCAGGCTGTCTGTCCTACCAA                30

                                                                                 F  G  S  G
            Y  F  C                                    TGACACTGGCTACAACAAGCCTACATTTGGCTCAGGGACTAGGCTGTCTGTCCTACCAA  Jα18
Vα3.20-5 : TACTTCTGT  GCTGTGAGTGATGCG----------------------ACTGGCTACAACAAGCCTACATTTGGCTCAGGGACTAGGCTGTCTGTCCTACCAA                27

                                                                                 F  G  S  G
            Y  F  C                                 TGATTCTGGATCCTACAACAAGCTGACCTTTGGCTCAGGAACCAGGCTGTCTGTCAAACCAA  Jα43
Vα3.20-6 : TACTTCTGT  GCTGTGGCGCGA-------------------------GGATCCTTCAACAAGCTGACCTTTGGCTCAGGAACTAGGCTGTCTGTCAAACCAA                33


                                                                                 F  G  S  G
            Y  F  C                                         TGAACTACGAGAAGCTCACATTTGGGAAGGGAACACGACTTCAGGTTTTGCCTA  Jα29
Vα3.21   : TACTTCTGT  TCTGTGAGTGATGCCCCCGGC----------------------------AAGCTCACATTTGGGAAGGGAACACGACTTCAGGTTTTGCCTA                21


                                                                                 F  G  S  G
            Y  L  C                                 TGATTCTGGATCCTACAACAAGCTGACCTTTGGCTCAGGAACCAGGCTGTCTGTCAAACCAA  Jα43
Vα3.22-1 : TACCTCTGT  GCTGTGAGGGGGCGCC----------------ATTCTGGATCCTACAACAAGCTGACCTTTGGCTCAGGAACCAGGCTGTCTGTCAAACCAA                33

                                                                                 F  G  S  G
            Y  L  C                                    TGACACTGGCTACAACAAGCTGACCTTTGGCTCAGGGACTAGGCTGTCTGTCCTACCAA  Jα49
Vα3.22-2 : TACCTCTGT  GCTCCGTA-------------------------TGACACTGGCTACAACAAGCTGACCTTTGGCTCAGGGACTAGGCTGTCTGTCCTACCAA                24

                                                                                 F  G  S  G
            Y  L  C                                 TGATTTTGGATCCTTCAACAAGCTGATCTTTGGCTCAGGAACCAGGCTGTCCGTCCTACCCA  Jα19
Vα3.22-3 : TACCTCTGT  GCTGTGCGCCTCG----------------------TTGGATCCTTCAACAAGCTGATCTTTGGCTCAGGAACCAGGCTGTCCGTCCTACCCA                27


                                                                                 F  G  K  G
            Y  L  C                                      TATACAATGCGGGCAAAGTCACTTTTGGAAAAGGAACTGTGCTTTCAGTTGTACCAG  Jα56
Vα3.23   : TACCTCTGT  GCTGTGAGTGATCCCCAAG-------------------ACAATGCGGGCAAAGTCACTTTTGGAAAAGGAACTGTGCTTTCAGTTGTACCAG                30
```

B

```
                                                Dδ1                           Dδ2
                                            TTGACTACGTAC               TTTTGGGATTGGAGTAC                                                                                          CDR3(nt)

            Y  F  C                                                                                                                    F  G  S  G
Vδ1       : TATTTCTGTGCTGTTCA                                                                                        ATAACAGATAAGCTTGTCTTTGGAAGTGGAACCACTCTCACAGTCGAACCAA  Jδ1
            TATTTCTGTGCTGTTC--------TAA-----TTGACTACGTAC-------G---------TTGGGATTGG-------------------------------------ACAGATAAGCTTGTCTTTGGAAGTGGAACCACTCTCACAGTCGAACCAA            39

            Y  F  C                                                                                                                    F  G  S  G
Vδ2.1     : TACTTCTGTGCAGTTCG                                                                                        ATAACAGATAAGCTTGTCTTTGGAAGTGGAACCACTCTCACAGTCGAACCAA  Jδ1
            TACTTCTGTGCAGTTCG--------C--------------------------------------------GAGTAC-----------------------------------GATAAGCTCGTCTTTGGAAGTGGAACCACTCTCACAGTCGAACCAA            18

            Y  F  C                                                                                                                    F  G  S  G
Vδ2.2     : TACTTCTGTGCAGTTCG                                                                                        ATAACAGATAAGCTTGTCTTTGGAAGTGGAACCACTCTCACAGTCGAACCAA  Jδ1
            TACTTCTGTGCAGTT-----------------------------------------------------------------GGGGGACGACTTTAC-------------ACAGATAAGCTTGTCTTTGGAAGTGGAACCACTCTCACAGTCGAACCAA            27

            Y  F  C                                                                                                                    F  G  S  G
Vδ2.3     : TACTTCTGTGCAGTTCC                                                                                        ATAACAGATAAGCTTGTCTTTGGAAGTGGAACCACTCTCACAGTCGAACCAA  Jδ1
Vδ2.3-1   : TACTTCTGTGCAGTGC----AGAGCGCCGAC--------------------------------GGGGTTGGAG---------------------------------CCCTGGATAAGCTTGTCTTTGGAAGTGGAACCACTCTCACAGTCGAACCAA            36
Vδ2.3-2   : TACTTCTGTGCAGTTC----GCCAACGCGGGG-------------------------------------GGAGTA-------------------------------------ATAAGCTTGTCTTTGGAAGTGGAACCACTCTCACAGTCGAACCAA            27

            Y  F  C                                                                                                                    F  G  S  G
Vδ2.10    : TACTTTTGTGCAGTTCC                                                                                        ATAACAGATAAGCTTGTCTTTGGAAGTGGAACCACTCTCACAGTCGAACCAA  Jδ1
Vδ2.10-1  : TACTTTTGTGCAGTTCC-------CGG-------GACTACG------------------TTTTGGGATTGGAGTAC---------GTCTG--------------------CAGATAAGCTTGTCTTTGGAAGTGGAACCACTCTCACAGTCGAACCAA            45
Vδ2.10-2  : TACTTCTGTGCAGT---------ACTC-------GACTACGTA------GCCGA-------TTGGGGTTGGAGTAC---------GGGCT----------------------ATAAGCTTGTCTTTGGAAGTGGAACCACTCTCACAGTCGAACCAA            45

            Y  F  C                                                                                                                    F  G  S  G
Vδ2.12    : TACTTCTGTGCAGTTCC                                                                                        ATAACAGATAAGCTTGTCTTTGGAAGTGGAACCACTCTCACAGTCGAACCAA  Jδ1
            TACTTCCGTGCAGTTC--------GAACG------------------------------TTTTGGGATTGGAG------------GTGG----------------------GATAAGCTTGTCTTTGGAAGTGGAACCACTCTCACAGTCGAACCAA            33

            Y  F  C                                                                                                                    F  G  S  G
Vδ2.14    : TACTTCTGTGCAGTTCG                                                                                        ATAACAGATAAGCTTGTCTTTGGAAGTGGAACCACTCTCACAGTCGAACCAA  Jδ1
            TACTTCTGTGCAGTTCG-----TATGGGGC---TGACTAC----------ACC-----------GGATTGGAGTAC-----------G--------------------ACAGATAAGCTTGTCTTTGGAAGTGGAACCACTCTCACAGTCGAACCAA            45

            Y  F  C                                                                                                                    F  G  K  G
Vδ3.1     : TATTTCTGTGCTGTTCG                                                                                       TGTTACTGCTCCTCTTATCTTCGGGAAGGGGACTCAACTGACCGTGGAACCAA  Jδ2
Vδ3.1-1   : TATTTCTGTGCTGTT--------------------ACTACGTA-------------------TGGGATTGGAGTAC---------GCCG------------------TACTGCTCCTCTTATCTTCGGGAAGGGGACTCAACTGACCGTGGAACCAA            39
Vδ3.1-2   : TATTTCTGTGCTGTTC-------ACGTA---------TACGTAC-------GG----------GGGATTGGAGTAC---------------------------------CTGCTCCTCTTATCTTCGGGAAGGGGACTCAACTGACCGTGGAACCAA            39
                                                                                                                                       F  G  S  G
                                                                                                                     ATAACAGATAAGCTTGTCTTTGGAAGTGGAACCACTCTCACAGTCGAACCAA  Jδ1
Vδ3.1-3   : TATTTCTGTGCTGT----------CCCC-----------CGTAC-----TTTCT------------ATTGG----------------------------------------GATAAGCTTGTCTTTGGAAGTGGAACCACTCTCACAGTCGAACCAA            27

            Y  F  C                                                                                                                    F  G  S  G
Vδ3.2     : TATTTCTGTGCTGTTCG                                                                                        ATAACAGATAAGCTTGTCTTTGGAAGTGGAACCACTCTCACAGTCGAACCAA  Jδ1
            TATTTCTGTGC-------------CCGA----TTGACTACGTA------------------------------------------TCCTC---------------------GATAAGCTTGTCTTTGGAAGCGGAACCACTCTCACAGTCGAACCAA  (*)       25

            Y  F  C                                                                                                                    F  G  S  G
Vδ3.3     : TATTTCTGTGCTGTTCG                                                                                        ATAACAGATAAGCTTGTCTTTGGAAGTGGAACCACTCTCACAGTCGAACCAA  Jδ1
            TATTTCTGTGCTGT---------CGAGAC--------------------------------------TTGGAG------------CCGTTC---------------------ATAAGCTTGTCTTTGGAAGTGGAACCACTCTCACAGTCGAACCAA  (*)       25
```

```
          Y  F  C                                                                                                                    F  G  S  G
Vδ5     : TATTTCTGCGTGGTTCG                                                                                        ATAACAGATAAGCTTGTCTTTGGAAGTGGAACCACTCTCACAGTCGAACCAA  Jδ1
Vδ5-1   : TATTTCTGCGTGGT----------AACG--------------------------------TTTGGGGTTGGA-------------TAGTG-----------------------TAAGCTTGTCTTTGGAAGTGGAACCACTCTCACAGTCGAACCAA  (*)      27
Vδ5-2   : TATTTCTGCGTGGTTCG-----------------------------------------------------------------GGTTGCCGTCC----------------CAGATAAGCTTGTCTTTGGAAGTGGAACCACTCTCACAGTCGAACCAA           24

          Y  L  C                                                                                                                    F  G  S  G
Vα1.1p  : TACCTGTGTGCACTGAGCC                                                                                      ATAACAGATAAGCTTGTCTTTGGAAGTGGAACCACTCTCACAGTCGAACCAA  Jδ1
          TACCTGTGTGCACTGAGC------GGAA----TTGACTACG-------CACGAGG------------TTGGA-------------AGGTG------------------------AAGCTTGTCTTTGGAAGTGGAACCACTCTCACAGTCGAACCAA  (*)      39

          Y  Y  C                                                                                                                    F  G  S  G
Vα2.1   : TATTACTGCGCCCTGAGAGC                                                                                     ATAACAGATAAGCTTGTCTTTGGAAGTGGAACCACTCTCACAGTCGAACCAA  Jδ1
          TATTACTGCGCCCTGAG--------TCA---------TACGTAC-----GGTCTCC------TGGGATTGGAGTAC---------GGAGG-----------------AACAGATAAGCTTGTCTTTGGAAGTGGAACCACTCTCACAGTCGAACCAA           51

          Y  Y  C                                                                                                                    F  G  S  G
Vα2.2   : TATTACTGCGCCCTGAGAGC                                                                                     ATAACAGATAAGCTTGTCTTTGGAAGTGGAACCACTCTCACAGTCGAACCAA  Jδ1
          TATTACTGCGCCCTGAGAGC--GAGGAGCTG---GACTACGT--------CCG-------TTTGGGA------------------CCTT-------------------ACAGATAAGCTTGTCTTTGGAAGTGGAACCACTCTCACAGTCGAACCAA           48

          Y  Y  C                                                                                                                    F  G  S  G
Vα2.3   : TATTACTGCGCCCTGAGAGC                                                                                     ATAACAGATAAGCTTGTCTTTGGAAGTGGAACCACTCTCACAGTCGAACCAA  Jδ1
Vα2.3-1 : TATTACTGCGCCCTGAGAGC-GCGACGATCAC--------------------------------GGATTGG-------------GATTGG--------------------AGATAAGCTTGTCTTTGGAAGTGGAACCACTCTCACAGTCGAACCAA           39
Vα2.3-2 : TATTACTGCGCCCTGAGAGC---------------ACTACGTAC-------C------------GGATTGGA---------------C--------------------------AAGCTTGTCTTTGGAAGTGGAACCACTCTCACAGTCGAACCAA           30
Vα2.3-3 : TATTACTGCGCCCTGAGAG-----GAAG---------TACGT--------TCC---------------TGGGGT----------TGGACG--------------------AGATAAGCTTGTCTCTGGAAGTGGAACCACTCTCACAGTCGAACCAA  (*)      38

          Y  L  C                                                                                                                    F  G  K  G
Vα3.2   : TACCTCTGTGCTGTGAATGA                                                                                    TGTTACTGCTCCTCTTATCTTCGGGAAGGGGACTCAACTGACCGTGGAACCAA  Jδ2
Vα3.2-1 : TACCTCTGTGCTGTGA--------------------------------------------------------------------CCCCGACC-------------------GCTCCTCTTATCTTCGGGAAGGGGACTCAACTGACCGTGGAACCAA           18
Vα3.2-2 : TACCTCTGTGCTGTGAATG--------------------------------------------------------GGCGAGGCGGACTCTACGGGGGAATAGA-TGTTACTGCTCCTCTTATCTTCGGGAAGGGGACTCAACTGACCGTCGAACCAA           48

          Y  L  C                                                                                                                    F  G  S  G
Vα3.3   : TACCTCTGTGCTGTGAATGA                                                                                     ATAACAGATAAGCTTGTCTTTGGAAGTGGAACCACTCTCACAGTCGAACCAA  Jδ1
Vα3.3-1 : TACCTCTGTGCTGTGA---------GTG---------TACGTAC------AAGG--------------TGGAGTA-----------AGG--------------------------AGCTTGTCTTTGGAAGTGGAACCACTCTCACAGTCGAACCAA           30
Vα3.3-2 : TACCTCTGTGCTGTGAATGA-----TAT-------------------------------------GATTGG---------------CCG---------------------------GCTTGTCTTTGGAAGTGGAACCACTCTCACAGTCGAACCAA           21

                                                                                                                                     F  G  K  G
                                                                                                                TGTTACTGCTCCTCTTATCTTCGGGAAGGGGACTCAACTGACCGTGGAACCAA  Jδ2
Vα3.3-3 : TACCTCTGTGCT------------AGGGG---------------------------------TGGGATTGGAGTAC----------GGG-------------------ACTGCTCCTCTTATCTTCGGGAAGGGGACTCAACTGACCGTGGAACCAA  (*)      31

          Y  L  C                                                                                                                    F  G  S  G
Vα3.4   : TACCTCTGTGCTGTGAATGA                                                                                     ATAACAGATAAGCTTGTCTTTGGAAGTGGAACCACTCTCACAGTCGAACCAA  Jδ1
Vα3.4-1 : TACCTCTGTGCTGTGA-------GACCGG---------ACGTAC--------------------------------------GGCCGAGGTAC------------------GATAAGCTTGTCTTTGGAAGCGGAACCACTCTCACAGTCGAACCAA           33
Vα3.4-2 : TACCTCTGTGCTGTGAA-------GCAC-----TGACTACGT--------CC--------TTTGGGATTGGAGT--------TCGCCTCCTAAT-----------ATAACAGATAAGCTTGTCTTTGGAAGTGGAACCACTCTCACAGTCGAACCAA  (*)      58
```

	V		J	CDR3 (nt)
		**D$_\delta$1** TTGACTACGTAC ; **D$_\delta$2** TTTTGGGATTGGAGTAC		**CDR3 (nt)**
	Y F C	F G S G		
		**ATAACAGATAAGCTTGTCTTTGGAAGTGGAACCACTCTCACAGTCGAACCAA**	**J$_\delta$1**	
V$_\delta$2.15-1 :	TACTTCTGT	GCAGTTCGAACCACGGG--TTGGAGTA---------GTCGT--------ATAACAGATAAGCTTGTCTTTGGAAGTGGAACCACTCTCACAGTCGAACCAA		39
V$_\delta$2.15-2 :	TACTTCTGT	GCACCCTTCTCCGAGGGG--ACAGATAAGCTTGTCTTTGGAAGTGGAACCACTCTTACAGTCGAACCAA		24
V$_\delta$2.15-3 :	TACTTCTGT	GCAGTCCCCTCC--------------GACTAC---------TTCC------TTTTGGG*G*TTGGAGTAC--------CCCAAC--------------------------TTGGAAGTGGAACCACTCTCACAGTCGAACCAA	(*)	36
	Y F C	F G K G		
		**TGTTACTGCTCCTCTTATCTTCGGGAAGGGGACTCAACTGACCGTGGAACCAA**	**J$_\delta$2**	
V$_\delta$2.16 :	TACTTCTGT	GCAGTA--------------------GACTAC--------TCGACC-------TTGGG*G*TTGGAGT-----------CGC----------------CTCCTCTTATCTTCGGGAAGGGGACTCAACTGACCGTGGAACCAA		36
	Y F C	F G S G		
		**ATAACAGATAAGCTTGTCTTTGGAAGTGGAACCACTCTCACAGTCGAACCAA**	**J$_\delta$1**	
V$_\delta$2.17 :	TACTTCTGT	GCAGTTCGCCGA--------------GACTACG----------G---------------TTGGAG-----------AACTC------------CAGATAAGCTTGTCTTTGGAAGTGGAACCACTCTCACAGTCGAACCAA		36
	Y F C	F G S G		
		**ATAACAGATAAGCTTGTCTTTGGAAGTGGAACCACTCTCACAGTCGAACCAA**	**J$_\delta$1**	
V$_\delta$2.18-1 :	TACTTTTGT	GCAGTTAAAC---GGG*G*TT---------------CGAGT-----------ACGTATAAGCTTGTCTTTGGAAGTGGAACCACTCTCACAGTCGAACCAA		27
		F G K G		
		**TGTTACTGCTCCTCTTATCTTCGGGAAGGGGACTCAACTGACCGTGGAACCAA**	**J$_\delta$2**	
V$_\delta$2.18-2 :	TACTTTTGT	GCAGTTCCGCGTG--------------------------------------TTTTGGG*G*TTGGA-------------CCGA-----------ACTGCTCCTCTTATCTTCGGGAAGGGGACTCAACTGACCGTGGAACCAA		36
	Y F C	F G K G		
		**TGTTACTGCTCCTCTTATCTTCGGGAAGGGGACTCAACTGACCGTGGAACCAA**	**J$_\delta$2**	
V$_\delta$2.19-1 :	TACTTCTGT	GCAGTTCGCGAGTACGAACTGC-------------------------------------TTGGAGTAC--------GTTCTA--------TTACTGCTCCTCTTATCTTCGGGAAGGGGACTCAACTGACCGTGGAACCAA	(*)	45
		F G S G		
		**ATAACAGATAAGCTTGTCTTTGGAAGTGGAACCACTCTCACAGTCGAACCAA**	**J$_\delta$1**	
V$_\delta$2.19-2 :	TACTTCTGT	GCAGTCCTACG--TTGGAG------------AAGT--------ATAACAGATAAGCTTGTCTTTGGAAGTGGAACCACTCTCACAGTCGAACCAA		30
	Y F C	F G S G		
		**ATAACAGATAAGCTTGTCTTTGGAAGTGGAACCACTCTCACAGTCGAACCAA**	**J$_\delta$1**	
V$_\delta$2.20 :	TACTTCTGT	GCAGTTAAAGTCGTA---------TTGACTAC---------CCG--------TTTGGG*G*TTGGAGTA---------AGTAGG-----------------AGCTTGTCTTTGGAAGTGGAACCACTCTCACAGTCGAACCAA	(*)	46
	Y Y C	F G S G		
		**ATAACAGATAAGCTTGTCTTTGGAAGTGGAACCACTCTCACAGTCGAACCAA**	**J$_\delta$1**	
V$_\alpha$2.5 :	TATTACTGC	GCCCTGAGA---------------TTGACTACGTAC--GATGGC-------------GATAAGCTTGTCTTTGGAAGTGGAACCACTCTCACAGTCGAACCAA		30
	Y Y C	F G K G		
		**TGTTACTGCTCCTCTTATCTTCGGGAAGGGGACTCAACTGACCGTGGAACCAA**	**J$_\delta$2**	
V$_\alpha$2.8 :	TATTACTGC	GCCCT---GAGTAC---------TCCA-----------ACTGCTCCTCTTATCTTCGGGAAGGGGACTCAACTGACCGTGGAACCAA		21
	Y Y C	F G K G		
		**TGTTACTGCTCCTCTTATCTTCGGGAAGGGGACTCAACTGACCGTGGAACCAA**	**J$_\delta$2**	
V$_\alpha$2.12-1 :	TATTACTGC	GCCCTCC--TTGGG*G*TTG---------------CGCA-------TGTTACTGCTCCTCTTATCTTCGGGAAGGGGACTCAACTGACCGTGGAACCAA		30
	Y Y C	F G S G		
		**ATAACAGATAAGCTTGTCTTTGGAAGTGGAACCACTCTCACAGTCGAACCAA**	**J$_\delta$1**	
V$_\alpha$2.12-2 :	TATTACTGC	GCCCTGAGAGCAATTTCTCG-----TGACTACGTA--------G-----------GGG*G*TTGG---------------C-----------TAACAGATAAGCTTGTCTTTGGAAGTGGAACCACTCTCACAGTCGAACCAA		48

```
                                                                                                                                 F  G  S  G
           Y  Y  C                                                                                             ATAACAGATAAGCTTGTCTTTGGAAGTGGAACCACTCTCACAGTCGAACCAA  Jδ1
Vα2.15   : TATTACTGC  GCCCTGAGAGCG--------------GACTACGTAC-----------------------------------------CG----------------GATAAGCTTGTCTTTGGAAGTGGAACCACTCTCACAGTCGAACCAA           27

                                                                                                                                 F  G  S  G
           Y  Y  C                                                                                             ATAACAGATAAGCTTGTCTTTGGAAGTGGAACCACTCTCACAGTCGAACCAA  Jδ1
Vα2.17-1 : TATTACTGC  GCCCTGTATC------------------CTACGT-------------------------------------------CGGA----------------------TGTCTTTGGAAGTGGAACCACTCTCACAGTCGAACCAA           15
Vα2.17-2 : TATTACTGC  GCCCTGAGACCC------------TTGACTACGTAC----------------------------------------GGGTTG-------------GATAAGCTTGTCTTTGGAAGTGGAACCACTCTCACAGTCGAACCAA           33
Vα2.17-4 : TATTACTGC  GCCCTAAC------------------GACTACGT--------------------TGGGGTTGGAGT------------CG-------------CAGATAAGCTTGTCTTTGGAAGTGGAACCACTCTCACAGTCGAACCAA  (*)      35
                                                                                                                                 F  G  K  G
                                                                                                              TGTTACTGCTCCTCTTATCTTCGGGAAGGGGACTCAACTGACCGTGGAACCAA  Jδ2
Vα2.17-3 : TATTACTGC  GCCCTGATCACTCC--------------------------------------TTTGGGGTTGGA-------------TCC-----------TACTGCTCCTCTTATCTTTGGGAAGGGGACTCAACTGACCGTGGAACCAA           36

                                                                                                                                 F  G  K  G
           Y  Y  C                                                                                            TGTTACTGCTCCTCTTATCTTCGGGAAGGGGACTCAACTGACCGTGGAACCAA  Jδ2
Vα2.18-1 : TATTACTGC  GCCCTGAGAGGAGG-----------------------------------------GGGAT-----------------GGAG----------TACTGCTCCTCTTATCTTCGGGAAGGGGACTCAACTGACCGTGGAACCAA           30
Vα2.18-3 : TATTACTGC  GCCCTGACTGAG----------------CTACGTAC------ATA------TTTTGGGGTTGGAGTAC-------GGGGTGTC------GTTACTGCTCCTCTTATCTTCGGGAAGGGGACTCAACTGACCGTGGAACCAA           57
                                                                                                                                 F  G  S  G
                                                                                                               ATAACAGATAAGCTTGTCTTTGGAAGTGGAACCACTCTCACAGTCGAACCAA  Jδ1
Vα2.18-2 : TATTACTGC  GCCC---------------------TGACTACGT------------------------------------------TGGCCC--------------ATAAGCTTGTCTTTGGAAGTGGAACCACTCTCACAGTCGAACCAA           21
Vα2.18-4 : TATTACTGC  GCCCTGAGGGG-----------------CTACGTAC-----GTTTA---------GGGGTTG---------------ACC-------------CACAGCCACTTGTCTTTGGAAGTGGAACCACTCTCACAGTCGAACCAA           39

                                                                                                                                 F  G  S  G
           Y  Y  C                                                                                             ATAACAGATAAGCTTGTCTTTGGAAGTGGAACCACTCTCACAGTCGAACCAA  Jδ1
Vα2.19   : TATTACTGC  GCCCTGAGAGGG--------------GACTAC--------------------TTTGGGGTTGGAGTAC--------------------------TCCTAAGCCTGTCTTTGGAAGTGGGACCACTCTCACAGTCGAACCAA  (*)      38

                                                                                                                                 F  G  S  G
           Y  Y  C                                                                                             ATAACAGATAAGCTTGTCTTTGGAAGTGGAACCACTCTCACAGTCGAACCAA  Jδ1
Vα2.20   : TATTACTGC  GCCC---------------------TGACTACGTA------G-------------GGGGTTGGAGT------------C-------------ACAGATAAGCTTGTCTTTGGAAGTGGAACCACTCTCACAGTCGAACCAA  (*)      33

                                                                                                                                 F  G  S  G
           Y  L  C                                                                                             ATAACAGATAAGCTTGTCTTTGGAAGTGGAACCACTCTCACAGTCGAACCAA  Jδ1
Vα3.11-1 : TACCTCTGT  GCTG-------------------------TACGT--------GTC----------GGGATTGGA--------AGGGTTATAGAG--------ACAGATAAGCTTGTCTTTGGAAGTGGAACCACTCTCGCAGTCGAACCAA           39
Vα3.11-2 : TACTTCTGT  GCTGTGCGTTCCGGGCGAA-----TTGACTACG-------CACCCC---------GGGGTTGGAGTAC----------GC----------------ATAAGCTTGTCTTTGGAAGTGGAACCACTCTCACAGTCGAACCAA           51
Vα3.11-3 : TACCTCTGT  GCTGTGCGTA-------------------------------------------TTGGGATTGGA------------CTTCGG----------ACAGATAAGCTTGTCTTTGGAAGTGGAACCACTCTCACAGTCGAACCAA           33

                                                                                                                                 F  G  S  G
           Y  L  C                                                                                             ATAACAGATAAGCTTGTCTTTGGAAGTGGAACCACTCTCACAGTCGAACCAA  Jδ1
Vα3.12   : TACCTCTGT  GCTGTGCGTGAGCGGGGCCCCC--------------------------------------------------------------------------ATAAGCTTGTCTTTGGAAGTGGAACCACTCTCACAGTCGAACCAA           24

                                                                                                                                 F  G  S  G
           Y  F  C                                                                                             ATAACAGATAAGCTTGTCTTTGGAAGTGGAACCACTCTCACAGTCGAACCAA  Jδ1
Vα3.13-1 : TACTTCTGT  GCTGTGAGTGACAC-------------------------------------TTTTGG-------------------GTTGG----------AACAGATAAGCTTGTCTTTGGAAGTGGAACCACTCTCACAGTCGAACCAA  (*)      32
Vα3.13-2 : TACTTCTGT  GCTGTGAGGCTCCGTCAC------------------------------------TGGGGTTG-------------TTCGTGCT--------------TAAGCTTGTCTTTGGAAGTGGAACCACTCTCACAGTCGAACCAA  (*)      35

                                                                                                                                 F  G  K  G
           Y  L  C                                                                                            TGTTACTGCTCCTCTTATCTTCGGGAAGGGGACTCAACTGACCGTGGAACCAA  Jδ2
Vα3.16-1 : TACCTCTGT  GCTGTCCAACCCA-----------TTGACTACG-------------------------------------------GGGGA--------GTTACTGCTCCTCTTATCTTCGGGAAGGGGACTCAACTGACCGTGGAACCAA           36
Vα3.16-2 : TACCTCTGT  GCTGGTACGCTTGTCGTATCG-----------------------------------------------------------------------ACTGCTCCTCTTATCTTCGGGAAGGGGACTCAACTGACCGTGGAACCAA           27
Vα3.16-5 : TACCTCTGT  GCTGTGCGTGTC--------------GACTACG------------------------------------------GTCCTTC-----------CTGCTCCTCTTATCTTCGGGAAGGGGACTCAACTGACCGTGGAACCAA  (*)      31
Vα3.16-9 : TACCTCTGT  GCTGTGCGTGATGGACG------------------------------------------TTGGAG------------GGTTA--------------CTCCTCTTATCTTCGGGAAGGGGACTCAACTGACCGTGGAACCAA           30
Vα3.16-10: TACCTCTGT  GCTGTGAA---------------------------------------------TTGGGATTGGA-----------TTATGTC---------TACTGCTCCTCTTATCTTCGGGAAGGGGACTCAACTGACCGTGGAACCAA           33
```

```
                                                                                                                          F  G  S  G
                                                                                          ATAACAGATAAGCTTGTCTTTGGAAGTGGAACCACTCTCACAGTCGAACCAA  Jδ1
Vα3.16-3 : TACCTCTGT  GCTGTGCGTGATCGGCG-----------CTACG----------G---------------TTGGAGTA---------TCCGC--------------GATAAGCTTGTCTTTGGAAGTGGAACCACTCTCACAGTCGAACCAA        39
Vα3.16-4 : TACCTCTGT  GCTGTGAAGGGGC------------------------------------------------GGAGTAC--------GTCTC------------CAGATAAGCTTGTCTTTGGAAGTGGAACCACTCTCACAGTCGAACCAA        30
Vα3.16-6 : TACCTCTGT  GCTGTGCGTGAG------------TTGAC--------------G---------TTGGGGTTGGAGT-----------GGA----------------------------TTGGAAGTGGAACCACTCTCACAGTCGAACCAA  (*)   25
Vα3.16-7 : TACCTCTGT  GCTGCCCCCCGGT----------------TACGTAC----GGGGCCG-----------ATTGGAGTAC---------AAAT-------------AGATAAGCTTGTCTTTGGAAGTGGAACCACTCTCACAGTCGAACCAA  (*)   45
Vα3.16-8 : TACCTCTGT  GCTGTGCGTGATCGCTC------------------------------------TTGGGGTTGGAGTAC--------G---------------ACAGATAAGCTTGTCTTTGGAAGTGGAACCACTCTCACAGTCGAACCAA        39
Vα3.16-11: TACCTCTGT  GTCGTAG----------------------TACGTAC-------------------------------------ACGGTGGGTTGG---------AGATAAGCTTGTCTTTGGAAGTGGAACCACTCTCACAGTCGAACCAA        30
Vα3.16-12: TACCTCTGT  GCTGTGAATGATC-------------GACTACGT---------------------GGGATTGGAG-------------------------TAACAGATAAGCTTGTCTTTGGAAGTGGAACCACTCTCACAGTCGAACCAA        39
Vα3.16-13: TACCTCTGT  GCTGTGCGTGACAAC----------------------------------------GGGGTTGGA-----------CCGCGTATG--------ACAGATAAGCTTGTCTTTGGAAGTGGAACCACTCTCACAGTCGAACCAA        39
Vα3.16-14: TACCTCTGT  GCTGTGCGTGACAGG--------------------------------------------TTGGAGTAC--------CTCAGT-------ATAACAGATAAGCTTGTCTTTGGAAGTGGAACCACTCTCACAGTCGAACCAA        39
```

```
                                                                                                                          F  G  S  G
            Y  F  C                                                                       ATAACAGATAAGCTTGTCTTTGGAAGTGGAACCACTCTCACAGTCGAACCAA  Jδ1
Vα3.24   : TACTTCTGT  GCTGTGGCGTTCCTCCTAA-----TTGACTACGTAC------------------------TGGAG------------GAG---------------GATAAGCTTGTCTTTGGAAGTGGAACCACTCTCACAGTCGAACCAA        42
```

C

```
                                                     Dβ
                                                GGGACAGGGGATC                                                                                 CDR3(nt)
                 Y  F  C                                                                                F  G  T  G
Vβ2          :   TATTTCTGCGCTAAGCAAGATA                                                         AATAATGAAAAACTGTTCTTTGGCACTGGGACAAAACTCACTGTCATAG   Jβ1.2
Vβ2-1(1)     :   TATTTCTGCGCTAAGGA--------GATTAAAGGACGATGG--------------------------------------------GAAAAACTGTTCTTTGGCACTGGGACAAAACTCACTGTCATAG         27
Vβ2-4(1)     :   TATTTCTGCGCTAAG----------TTGCACCGGGACAG------------------------------------------TAATGAAAAACTGTTCTTTGGCACTGGGACAAAACTCACTGTCATAG         27
Vβ2-6(1)     :   TATTTCTGCGCTAAGCAAGATA---------TGC----------------CAGGGGATC-----TCGCGCTGGATGCG-------GAAAAACTGTTCTTTGGCACTGGGACAAAACTCACTGTCATAG         42
Vβ2-8(1)     :   TATTTCTGCGCTAAGCAAGAT-----------GG------------------GGGAT----------GGGTAG--------TAATGAAAAACTGTTCTTTGGCACTGGGACAAAACTCACTGTCATAG         33
Vβ2-9(1)     :   TATTTCTGCGCTAAGCAAGA-------GAAGGAGGGCG-----------------------------------------------GAAAAACTGTTCTTTGGCACTGGGACAAGACTCACTGTCATAG  (*)    25
Vβ2-10(1)    :   TATTTCTGCGCTAAGCAAGATA------GGAGCTGG----------------GGGGATC-------GTCCCCG-----------TGAAAAACTGTTCTTTGGCACTGGGACAAAACTCACTGTCATAG         39
Vβ2-12(1)    :   TATTTCTGCGC----------------CACGGGCCGA--------------AGGGGATC-----GGGGTTAGCGAC--------TGAAAAACTGTCCTTTGGCACTGGGACAAAACTCACTGTCATAG         36
Vβ2-13(1)    :   TATTTCTGCGCTAAGCAAGAT----CTCGCTTCCGTTCGC------GGGACAGGGGAT---------TACGGG---------AATGAAAAACTGTTCTTTGGCACTGGGACAAAACTCACTGTCATAG         51
Vβ2-14(1)    :   TATTTCTGCGCTAAGCAAGAT-----CCGGCCGACAGTC-------GGGACAGGG------------CTACCA-------------------------TTGGCACTGGGACAAAACTCACTGTCATAG  (*)    30

                 Y  F  C                                                                                F  G  F  G
Vβ2          :   TATTTCTGCGCTAAGCAAGATA                                                         ACCAACACTAAGGTGATTTTTGGTTTTGGCACAAAAGTTACAGTGATGG   Jβ1.1
Vβ2-2(1)     :   TATTTCTGCGC-----------------CGCGGCGGTAC-----------------------------------------CCAACACTAAGGTGATTTTTGGTTTTGGCACAAAAGTTACAGTGATGG         21
Vβ2-3(1)     :   TATTTCTGCGCTAAGCA--------------GGGT----------------AGGGGA--------CACGGACTAT--------ACACTAAGGTGATTTTTGGTTTTGGCTCAAAAGTTACAGTGATGG  (*)    33
Vβ2-5(1)     :   TATTTCTGCGCTAAGCAAGA---------CGCCTAC----------GGGACAG-------------CCTTACTT-----ACCAACACTAAGGTGATTTTTGGTTTTGGCACAAAAGTTACAGTGATGG         42

                 Y  F  C                                                                                F  G  K  G
Vβ2          :   TATTTCTGCGCTAAGCAAGATA                                                         CAATCTCAACAACTGCATTTTGGAAAGGGCACCCAGCTGACAGTGCTTG   Jβ2.2
Vβ2-21(2)    :   TATTTCTGCGCTA-----------------TCGAC-----------GGGAC------------------G---------CAATCTCAACAACTGCATTTTGGAAAGGGCACCCAGCTGACAGTGCTTG         24

                 Y  F  C                                                                                F  G  E  G
Vβ2          :   TATTTCTGCGCTAAGCAAGATA                                                        TTTTATCAATACCCAGTATTTTGGAGAAGGAACAAAAATAACAGTTCTGG   Jβ2.3
Vβ2-15(2)    :   TATTTCTGCGCTAAGCAAGATA--CCTTACGACGGACCACTGC--------------------------------------TATCAATACCCAGTATTTTGGAGAAGGAACAAAAATAACAGTTCTGG  (*)    39
Vβ2-16(2)    :   TATTTCTGCGCTAAGCA--------------TC---------------GACAGGGGA----------CGGAG-------------AATACCCAGTATTTTGGAGAAGGAACAAAAATAACAGTTCTGG         27
Vβ2-18(2)    :   TATTTCTGCGCTAAGCA------------CCGGGCG----------GGGACA----------------CTCT-----------TCAATACCCAGTATTTTGGAGAAGGAACAAAAATAACAGTTCTGG  (*)    30
Vβ2-19(2)    :   TATTTCTGCGCTAAGCAAGAT-------------------------GGGACAGG-----------TCCTTCGGGC-------------ACCCAGTATTTTGGAGAAGGAACAAAAATAACAGTTCTGG         30
Vβ2-20(2)    :   TATTTCTGCGCTAAGCAAGAT-------GAGACGAGGC------------CAGGG------------AGTAG------------CAATACCCAGTATTTTGGAGAAGGAACAAAAATAACAGTTCTGG         36

                 Y  Y  C                                                                                F  G  P  G
Vβ2          :   TATTTCTGCGCTAAGCAAGATA                                                          ATACATATGAGGTTGAGTTTGGTCCTGGGACCCACATAACAGTACTAG   Jβ2.1
Vβ2-17(2)    :   TATTTCTGCGCTAAG------------AGGTCCGAAGCT---------------------------------------------ATATGAGGTTGAGTTTGGTCCTGGGACCCACATAACTGTACTAG  (*)    22
Vβ2-22(2)    :   TATTTCTGCGCTAAGCAAGATA-------------------------GGACAGG-------------ACGGG--------------ATGAGGTTGAGTTTGGTCCTGGGACCCACATAACAGTACTAG         27
Vβ2-23(2)    :   TATTTCTGCGCTAAGC-------------GGGGTC-------------GACAGG---------------CGG--------ATACATATGAGGTTGAGTTTGGTCCTGGGACCCACATAACAGTACTAG  (*)    30


                 Y  Y  C                                                                                F  G  E  G
Vβ3.1(3.4)   :   TATTACTGTGCTGAAAGTGAAA                                                        TTTTATCAATACCCAGTATTTTGGAGAAGGAACAAAAATAACAGTTCTGG   Jβ2.3
Vβ3.1-7(2)   :   TATTACTGTGCTGAA--------------GTAACCGATC-------GGGACAGG-------------------------------AATACCCAGTATTTTGGAGAAGGAACAAAAATAACAGTTCTGG         27

                 Y  Y  C                                                                                F  G  T  G
Vβ3.1(3.4)   :   TATTACTGTGCTGAAAGTGAAA                                                         AATAATGAAAAACTGTTCTTTGGCACTGGGACAAAACTCACTGTCATAG   Jβ1.2
Vβ3.1-2(1)   :   TATTACTGTGCTGAAA------------AGGGAGTACGCG------------GGGGA----------------------------GAAAAACTGTTCTTTGGCACTGGGACAAAACTCACTGTCATAG         27
Vβ3.1-3(1)   :   TATTACTGTGCTGAAAGTG---------GCGTCCGGGACTC----------------------------------------TAATGAAAAACTGTTCTTTGGCACTGGGACAAAACTCACTGTCATAG         30
Vβ3.1-4(1)   :   TATTACTGTGCTGAAAGTGAA----------GCCGGT-----------GACAGGGGATC---------GGG-------------TGAAAAACTGTTCTTTGGCACTGGGACAAAACTCACTGTCATAG         36
Vβ3.1-5(1)   :   TATTACTGTGCTGAAAGTGAA------GCGACCGAGCGAGG-------------------------------------------TGAAAAACTGTTCTTTGGCACTGGGACAAAACTCACTGTCATAG         30

                 Y  Y  C                                                                                F  G  P  G
Vβ3.1(3.4)   :   TATTACTGTGCTGAAAGTGAAA                                                          ATACATATGAGGTTGAGTTTGGTCCTGGGACCCACATAACAGTACTAG   Jβ2.1
Vβ3.1-6(2)   :   TATTACTGTGCTGAA---------------GTCTTTGC---------------GGGAT----------TC---------------------GTTGAGTTTGGTCCTGGGACCCACATAACAGTACTAG         18
Vβ3.1-10(2)  :   TATTACTGTGCTGAAAGT--------------CCG---------------CAGGG-----------CCCTCT------------------GGTTGAGTTTGGTCCTGGGACCCACATAACAGTACTAG         21
Vβ3.1-13(2)  :   TATTACTGTGCTGAAAGTGAA--------------------------------GGGATC---------GG-----------------TGAGGTTGAGTTTGGTCCTGGGACCCACATAACAGTACTAG   (*)   21

                 Y  Y  C                                                                                F  G  K  G
Vβ3.1(3.4)   :   TATTACTGTGCTGAAAGTGAAA                                                         CAATCTCAACAACTGCATTTTGGAAAGGGCACCCAGCTGACAGTGCTTG   Jβ2.2
Vβ3.1-8(2)   :   TATTACTGTGCTGA-----------GCTCGATCAGGTGAGGG--------------------------------------------AACAACTGCATTTTGGAAAGGGCACCCAGCTGACAGTGCTTG         24
Vβ3.1-9(2)   :   TATTACTGTGCTGAAAGT--------------CCC-----------GGGACAGGGGATC------CTCAGAGAG------------AACAACTGCATTTTGGAAAGGGCACCCAGCTGACAGTGCTTG         36
```

```
Vβ3.1-11(2)    : TATTACTGTGCTGAAAGTGA---------GAGGCGGGAC--------------GGGAT-------TAACTATCC-------ATCTCAACAACTGCATTTTGGAAAGGGCACCCAGCTGACAGTGCTTG               42
Vβ3.1-12(2)    : TATTACTGTGCTGAAAGTGAA--------TGCGATTCGG------------AGGGG------------CAC--------------CAACAACTGCATTTTGGAAAGGGCACCCAGCTGACAGTGCTTG               33


                 Y  Y  C                                                                                          F  G  T  G
Vβ3.2          : TATTACTGTGCTGAAAGTGAAA                                                         AATAATGAAAAACTGTTCTTTGGCACTGGGACAAAACTCACTGTCATAG  Jβ1.2
Vβ3.2-1(1)     :                       --------CGCGGGAC--------GGGAC--------------GCGTCCG---------TAATGAAAAACTGTTCTTTGGCACTGGGACAAAACTCACTGTCATAG  (*)    27
Vβ3.2-2(1)     : TATTACTGTGCTGAAAGTG-----------CCGGGGCC----------GACAG-----------ACCCGGGGGC--------AATGAAAAACTGTTCTTTGGCACTGGGACAAAACTCACTGTCATAG         39
Vβ3.2-3(1)     : TATTACTGTGCTGAAAGTG-------CCGGGTCTGAAGCGG---------CAGGGGAT-------AGCGAGGG-----------TGAAAAACTGTTCTTTGGCACTGGGACAAAACTCACTGTCATAG         45
Vβ3.2-5(1)     : TATTACTGTGCTGAAAGT-----------TCGAGTCCCC-------GGGAC----------------------------------GAAAAACTGTTCTTTGGCACTGGGACAAAACTCACTGTCATAG         27
Vβ3.2-6(1)     : TATTACTGTGCTGAA----------------GGTAGCC--------GGGACAG-------------CCGCG------------ATGAAAAACTGTTCTTTGGCACTGGGACAAAACTCACTGTCATAG         30
Vβ3.2-7(1)     : TATTACTGTGCTGAAAGTG------CCTCGGCGGCTGACGGCAG-------------------------------------TAATGAAAAACTGTTCTTTGGCACTGGGACAAAACTCACTGTCATAG         30
Vβ3.2-8(1)     : TATTACTGTGCTGAAAGTGAA------------CA------------------GGGATC---------G----------------GAAAAACTGTTCTTTGGCACTGGGACAAAACTCACTGTCATAG         24
Vβ3.2-9(1)     : TATTACTGTGCTGAA---------------CTACGGGG-----------------------------------------------GAAAAACTGTTCTTTGGCACTGGGACAAAACTCACTGTCATAG         18

                 Y  Y  C                                                                                          F  G  E  G
Vβ3.2          : TATTACTGTGCTGAAAGTGAAA                                                        TTTTATCAATACCCAGTATTTTGGAGAAGGAACAAAAATAACAGTTCTGG  Jβ2.3
Vβ3.2-12(2)    : TATTACTGTGCTGAAAGTGA---CGGTCGAGAGGGCCCGACCAGA----------------------------------------AATACCCAGTATTTTGGAGAAGGAACAAAAATAACAGTTCTGG         36
Vβ3.2-15(2)    : TATTACTGTGCTGAAACTGA-----TACGCGACCGGGGGTTCCG-----------------------------------------AATACCCAGTATTTTGGAGAAGGAACGAAAATAACAGTTCTGG         33

                 Y  Y  C                                                                                          F  G  P  G
Vβ3.2          : TATTACTGTGCTGAAAGTGAAA                                                          ATACATATGAGGTTGAGTTTGGTCCTGGGACCCACATAACAGTACTAG  Jβ2.1
Vβ3.2-10(2)    : TATTACTGTGCTGAAAGTGAA-----CGGATGGCGCCCTTCGG--------------------------------------------TGAGGTTGAGTTTGGTCCTGGGACCCACATAACAGTACTAG         30
Vβ3.2-13(2)    : TATTACTGTGCTGAAAGTGAA-----------GGG--------------ACAGGGG------------------------------------TTGAGTTTGGTCCTGGGACCCACATAACAGTACTAG  (*)    18
Vβ3.2-14(2)    : TATTACTGTGCTGAAA---------------CTCGG---------------AGGGGATC---------CGA-------------ATATGAGGTTGAGTTTGGTCCTGGGACCCACATAACAGTACTAG         27

                 Y  Y  C                                                                                          F  G  K  G
Vβ3.2          : TATTACTGTGCTGAAAGTGAAA                                                         CAATCTCAACAACTGCATTTTGGAAAGGGCACCCAGCTGACAGTGCTTG  Jβ2.2
Vβ3.2-11(2)    : TATTACTGTGCTGAAAGTGAA----------GATCGG---------------GGGGATC---------------------------AACAACTGCATTTTGGAAAGGGCACCCAGCTGACAGTGCTTG         27


                 Y  Y  C                                                                                          F  G  T  G
Vβ3.3          : TATTACTGTGCTGAAAGTGAAA                                                         AATAATGAAAAACTGTTCTTTGGCACTGGGACAAAACTCACTGTCATAG  Jβ1.2
Vβ3.3-1(1)     : TATTACTGTGCTGAAAGTG------------CTAAGG-------------CAGGGGA----------CGTC-----------AATGAAAAACTGTTCTTTGGCACTGGGACAAAACTCACTGTCATAG         33
Vβ3.3-2(1)     : TATTACTGTGCTGAAAG-----------------C------------GGACAGGG-------------CGG----------------AAAACTGTTCTTTGGCACTGGGACAAAACTCACTGTCATAG         21

                 Y  Y  C                                                                                          F  G  P  G
Vβ3.3          : TATTACTGTGCTGAAAGTGAAA                                                          ATACATATGAGGTTGAGTTTGGTCCTGGGACCCACATAACAGTACTAG  Jβ2.1
Vβ3.3-6(2)     : TATTACTGTGCTGAA----------------GCGC-----------GGGACAGGG-------------AAT---------ATACATATGAGGTTGAGTTTGGTCCTGGGACCCACATAACAGTACTAG         30
Vβ3.3-8(2)     : TATTACTGTGCTGAAAG--------------CGAC-----------GGGAC-------------TACTACGGGC--------------GAGGTTGAGTTTGGTCCTGGGACCCACATAACAGTACTAG         27
Vβ3.3-9(2)     : TATTACTGTGCTGAAAG------------CTACTCCC---------GGGAC--------------GGGGAACTC--------------GAGGTTGAGTTTGGTCCTGGGACCCACATAACAGTACTAG         30

                 Y  Y  C                                                                                          F  G  F  G
Vβ3.3          : TATTACTGTGCTGAAAGTGAAA                                                         ACCAACACTAAGGTGATTTTTGGTTTTGGCACAAAAGTTACAGTGATGG  Jβ1.1
Vβ3.3-5(1)     : TATTACTGTG-----------------------T---------------ACAGGGGA--------------------------ACACTAAGGTGATTTTTGGTTTTGGCACAAAAGTTACAGTGATGG         15

                 Y  Y  C                                                                                          F  G  E  G
Vβ3.3          : TATTACTGTGCTGAAAGTGAAA                                                        TTTTATCAATACCCAGTATTTTGGAGAAGGAACAAAAATAACAGTTCTGG  Jβ2.3
Vβ3.3-7(2)     : TATTACTGTGCTGAAAG--------------CGCC-----------GGGACAGGGG------------CG---------------AATACCCAGTATTTTGGAGAAGGAACAAAAATAACAGTTCTGG         27

                 Y  Y  C                                                                                          F  G  K  G
Vβ3.3          : TATTACTGTGCTGAAAGTGAAA                                                         CAATCTCAACAACTGCATTTTGGAAAGGGCACCCAGCTGACAGTGCTTG  Jβ2.2
Vβ3.3-10(2)    : TATTACTGTGCTGAAAGTGAAA---------CGGTT----------------GGGGATC------GCCCACAGA------AATCACAACAACTGCATTTTGGAAAGGGCACCCAGCTGACAGTGCTTG         42


                 Y  Y  C                                                                                          F  G  T  G
Vβ3.5          : TATTACTGTGCTGAAAGTGAAA                                                         AATAATGAAAAACTGTTCTTTGGCACTGGGACAAAACTCACTGTCATAG  Jβ1.2
Vβ3.5-1(1)     : TATTACTGTGCTGAAAGTGAAA-----TTGGTCGGGGG--------------------------------------------AATGAAAAACTGTTCTTTGGCACTGGGACAAAACTCACTGTCATAG         30
```

```
Vβ3.5-2(1)   : TATTACTGTGCTGAAAGTGA-------CCCTCTATTTGG-------------------------------------------------AAAACTGTTCTTTGGCACTGGGACAAAACTCACTGTCATAG          24
Vβ3.5-6(1)   : TATTACTGTGCTGAAAGTG------------CT----------------ACAGGGGATC----------GG------------AATGAAAAACTGTTCTTTGGCGCTGGGACAAAACTCACTGTCATAG          30
Vβ3.5-8(1)   : TATTACTGTGCTGAAAGTGAA-------CCCGAGA-------------GACAGGGG---------TCACGCACGG---------ATGAAAAACTGTTCTTTGGCACTGGGACAAAACTCACTGTTATAG          39
Vβ3.5-10(1)  : TATTACTGTGCTGAAAGTG-----------CTCC------------GGGACAGGGGA----------GATACGG--------TAATGAAAAACTGTTCTTTGGCACTGGGACAAAACTCACTGTCATAG          39

               Y  Y  C                                                                                           F  G  K  G
Vβ3.5        : TATTACTGTGCTGAAAGTGAAA                                                          CAATCTCAACAACTGCATTTTGGAAAGGGCACCCAGCTGACAGTGCTTG   Jβ2.2
Vβ3.5-3(2)   : TATTACTGTGCTGAAAGTGAAA-------GGTTTTG----------GGGACA-------------CCAGCTTGGG--------TCTCAACAACTGCATTTTGGAAAGGGCACCCAGCTGACAGTGCTTG          42
Vβ3.5-7(2)   : TATTACTGTGCTGAA-------------GAGGCCTTC---------GGGACAGGG-----------CGGTTG-----------------CAACTGCATTTTGGAAAGGGCACCCAGCTGACAGTGCTTG          30

               Y  Y  C                                                                                           F  G  E  G
Vβ3.5        : TATTACTGTGCTGAAAGTGAAA                                                         TTTTATCAATACCCAGTATTTTGGAGAAGGAACAAAAATAACAGTTCTGG   Jβ2.3
Vβ3.5-4(2)   : TATTACTGTGCTGAAAGTGAA--CAGGCGCCGCGTGGTAGTCTGGA---------------------------------------CAATACCCAGTATTTTGGAGAAGGAACAAAAATAACAGTTCTGG          39
Vβ3.5-11(2)  : TATTACTGTGCTGAA---------------CTCGTAC---------------GGGGATC----------GG----------------ATACCCAGTATTTTGGAGAAGGAACAAAAATAACAGTTCTGG          24

               Y  Y  C                                                                                           F  G  P  G
Vβ3.5        : TATTACTGTGCTGAAAGTGAAA                                                           ATACATATGAGGTTGAGTTTGGTCCTGGGACCCACATAACAGTACTAG   Jβ2.1
Vβ3.5-5(2)   : TATTACTGTGCTGAAAGTGAA-----GCCGCGATAGGTCG------------------------------------------------TGAGGTTGAGTTTGGTCCTGGGACCCACATAACAGTACTAG          27
Vβ3.5-9(2)   : TATTACTGTGCTGAAA-----------------C--------------GACAGG-------------CTAC------------------GAGGTTGAGTTTGGTCCTGGGACCCACATAACAGTACTAG          18

               Y  Y  C                                                                                           F  G  F  G
Vβ3.5        : TATTACTGTGCTGAAAGTGAAA                                                          ACCAACACTAAGGTGATTTTTGGTTTTGGCACAAAAGTTACAGTGATGG   Jβ1.1
Vβ3.5-12(2)  : TATTACTGTGCTGAA---------------GCCGT---------------CAGGGGA--------GGAACGTCTCGT---ACCAACACTAAGGTGATTTTTGGTTTTGGCACAAAAGTTACAGTGATGG          39


               Y  F  C                                                                                           F  G  T  G
Vβ4          : TATTTCTGT                                                                       AATAATGAAAAACTGTTCTTTGGCACTGGGACAAAACTCACTGTCATAG   Jβ1.2
Vβ4-1(1)     : TATTTCTGT  GCCAGCACCAGGGGC------------------------CAGGGGATC----GTCGTGTCACGAGGG--------GAAAAACTGTTCTTTGGCACTGGGACAAAACTCACTGTCATAG          42
Vβ4-2(1)     : TATTTCTGT  GCCAGCAGTGCGCCCGGACGTCTCGGAG---------------------------------------------ATGAAAAACTGTTCTTTGGCACTGGGACAAAACTCACTGTCATAG          33
Vβ4-3(1)     : TATTTCTGT  GCCAGCAGACCGAAAAAAC----------------GGGACAGG-------------CTCG-------------ATGAAAAACTGTTCTTTGGCACTGGGACAAAACTCACTGTCATAG          36
Vβ4-4(1)     : TATTTCTGT  GCCAGCAGTTTGAAC---------------------------GGGAT--------AGTTTTGC----------ATGAAAAACCGTTCTTTGGCACTGGGACAAAACTCACTGTCATAG          33
Vβ4-5(1)     : TATTTCTGT  GCCAGCAGTTTTGAA---------------------------GGGATC----------G--------------ATGAAAAACTGTTCTTTGGCACTGGGACAAAACTCACTGTCATAG          27
Vβ4-6(1)     : TATTTCTGT  GCCAGCAGTT-----------------------------CAGGGGATC----------CC------------------AAACTGTTCTTTGGCACTGGGACAAAACTCACTGTCATAG          21
Vβ4-7(2)     : TATTTCTGT  GCCAGCAGCGGCGC-----------------------GACAGGG------------TCCGT-------------TGAAAAACTGTTCTTTGGCACTGGGACAAAACTCACTGTCATAG   (*)    30

               Y  F  C                                                                                           F  G  P  G
Vβ4          : TATTTCTGT                                                                         ATACATATGAGGTTGAGTTTGGTCCTGGGACCCACATAACAGTACTAG   Jβ2.1
Vβ4-8(2)     : TATTTCTGT  GCCAGCAGTCA-------------------------GGACAGG-------------ATAAGA----------------GAGGTTGAGTTTGGTCCTGGGACCCACATAACAGTACTAG          24
Vβ4-9(2)     : TATTTCTGT  GCCAGCAGTTTGATAC-------------------GGGACAGGG-----------CGTATAA------------ATATGAGGTTGAGTTTGGTCCTGGGACCCACATAACAGTACTAG          36


                                                                                                                 F  G  T  G
               Y  Y  C                                                                          AATAATGAAAAACTGTTCTTTGGCACTGGGACAAAACTCACTGTCATAG   Jβ1.2
Vβ3.6-1(1)   : TATTACTGT  GCTGAAAGTGAGGCCC--------------------------GGGATC---------GAACG----------AATGAAAAACTGTTCTTTGGCACTGGGACAAAACTCACTGTCATAG          33
Vβ3.6-2(1)   : TATTACTGT  GCTGA---------------------------------ACAGGGGATC-------GCCCCGGGG--------AATGAAAAACTGTTCTTTGGCACTGGGACAAAACTCACTGTCATAG          30

                                                                                                                 F  G  K  G
               Y  Y  C                                                                          CAATCTCAACAACTGCATTTTGGAAAGGGCACCCAGCTGACAGTGCTTG   Jβ2.2
Vβ3.6-3(1)   : TATTACTGT  GCTGAAAGACGAA-----------------------GGACAGGG--------------AGG--------CAATCTCAACAACTGCATTTTGGAAAGGGCACCCAGCTGACAGTGCTTG          33

                                                                                                                 F  G  E  G
               Y  Y  C                                                                         TTTTATCAATACCCAGTATTTTGGAGAAGGAACAAAAATAACAGTTCTGG   Jβ2.3
Vβ3.6-4(2)   : TATTACTGT  GCTGAAAGTGAAATTGAGGGC---------------GGACA-----------------CCC-------------CAATACCCAGTATTTTGGAGAAGGAACAAAAATAACAGTTCTGG          33

                                                                                                                 F  G  P  G
               Y  Y  C                                                                            ATACATATGAGGTTGAGTTTGGTCCTGGGACCCACATAACAGTACTAG   Jβ2.1
Vβ3.6-5(2)   : TATTACTGT  GCTGAACCTTCCCCGGTGGGGCCGAAC--------GGGACAGGGGATC----------GGA---------------ATGAGGTTGAGTTTGGTCCTGGGACCCACATAACAGTACTAG          45
Vβ3.6-6(2)   : TATTACTGT  GCTGAAAGTGAAGGACCCCACTTGTCCC------------------------------------------------------TTGAGTTTGGTCCTGGGACCCACATAACAGTACTAG          24
```

```
                                                                                                                F  G  F  G
               Y  Y  C                                                                       ACCAACACTAAGGTGATTTTTGGTTTTGGCACAAAAGTTACAGTGATGG  Jβ1.1
Vβ3.7-1(1)  : TATTACTGT  GCTGAAATACTTCC---------------------GGGACAGG-------------ACGGCG----------ACACTAAGGTGATTTTTGGTTTTGGCACAAAAGTTACAGTGATGG           33

                                                                                                                F  G  P  G
               Y  Y  C                                                                        ATACATATGAGGTTGAGTTTGGTCCTGGGACCCACATAACAGTACTAG  Jβ2.1
Vβ3.7-2(2)  : TATTACTGT  GCTGAAAGT--------------------------GGGACAGGGGAT-----------ATC----------------GAGGTTGAGTTTGGTCCTGGGACCCACATAACAGTACTAG           24

                                                                                                                F  G  E  G
               Y  Y  C                                                                       TTTTATCAATACCCAGTATTTTGGAGAAGGAACAAAAATAACAGTTCTGG  Jβ2.3
Vβ3.7-3(2)  : TATTACTGT  GCTGAAAGTGAATACGGGG--------------------------------------------------TTATCAATACCCAGTATTTTGGAGAAGGAACAAAAATAACAGTTCTGG           27
```

D

```
                                                                                                             CDR3(nt)
          Y  Y  C                                                                 F  G  T  G
Vγ1.1   : TACTACTGTGCTTACTGGGACTCT                           TCTGGTTATTACTACAAAGTGTTTGGCACCGGTACAAAGCTCATCGTGTCAG  Jγ3
Vγ1.1-1 : TACTACTGTGCTTACTGGGACTCT---------------GACG---------CTGGTTATTACTACAAAGTGTTTGGCACCGGTACAAAGCTCATCGTGTCAG           30
Vγ1.1-4 : TACTACTGTGCTTACTGGGAC------------------GGG---------------------------GTGTTTGGCACCGGTACAAAGCTCATCGTGTCAG            9
Vγ1.1-7 : TACTACTGTGCTTACTGGGAC------------------CGA---------TCTGGTTATTACTACAAAGTGTTTGGCACCGGTACAAAGCTCATCGTGTCAG           27

          Y  Y  C                                                                 F  G  T  G
Vγ1.1   : TACTACTGTGCTTACTGGGACTCT                             GACCACATGGATTAAATACTTCGGTACAGGAACTAAGCTAATTATCTCCG  Jγ1
Vγ1.1-2 : TACTACTGTGCTTACTGGGAC-----------------CGAGT----------------ATGGATTAAATACTTCGGTACAGGAACTAAGCTAATTATCTCCG           21
Vγ1.1-5 : TACTACTGTGCTTACTGGGACTCT---------------ACG------------------TGGATTAAATACTTCGGTACAGGAACTAAGCTAATTATCTCCG           21
Vγ1.1-8 : TACTACTGTGCTTACTGGGACTCT--------------TGGC-------------------GGATTAAATACTTCGGTACAGGAACTAAGCTAATTATCTCCG           21
Vγ1.1-9 : TACTACTGTGCTTACTGGGACTCT------------GCCATCTC---------GACCACATGGATTAAATACTTCGGTACAGGAACTAAGCTAATTATCTCCG           33

          Y  Y  C                                                                 F  G  T  G
Vγ1.1   : TACTACTGTGCTTACTGGGACTCT                                    GGCAATCTGGTCTTTGGCACAGGCACTAAACTCATCGTTTCCA  Jγ4(Jγ5)
Vγ1.1-3 : TACTACTGTGCTTACTGGGACTCT-------------ATCGTTG-----------------------TGGTCTTTGGCACAGGCACTAAACTCATCGTTTCCA           18
Vγ1.1-6 : TACTACTGTGCTTACTGGGACTCT------------------------------------------CTGGTCTTTGGCACAGGCACTAAACTCATCGTTTCCA           12


          Y  Y  C                                                                 F  G  T  G
Vγ1.2   : TACTACTGTGCCTACTGGGACTCT                           TCTGGTTATTACTACAAAGTGTTTGGCACCGGTACAAAGCTCATCGTGTCAG  Jγ3
Vγ1.2-1 : TACTACTGTGCCTACTGGGA-------------------A-----------TCTGGTTATTACTACAAAGTGTTTGGCACCGGTACAAAGCTCATCGTGTCAG           18
Vγ1.2-3 : TACTACTGTGCCTACTGGGACTCT------------------------------GGTTATTACTACAAAGTGTTTGGCACCGGTACAAAGCTCATCGTGTCAG           24
Vγ1.2-4 : TACTACTGTGCCTACTGGGACTCT-----------AGAGGTGCCTC-------------TTACTACAAAGTGTTTGGCACCGGTACAAAGCTCATCGTGTCAG           30

          Y  Y  C                                                                 F  G  T  G
Vγ1.2   : TACTACTGTGCCTACTGGGACTCT                             GACCACATGGATTAAATACTTCGGTACAGGAACTAAGCTAATTATCTCCG  Jγ1
Vγ1.2-2 : TACTACTGTGCCTACTGGGACTC----------------CG-----------------CATGGATTAAATACTTCGGTACAGGAACTAAGCTAATTATCTCCG           21


          Y  Y  C                                                                 F  G  T  G
Vγ1.3p  : TACTACTGTGCCTACTGGGACTCT                                    GGCAATCTGGTCTTTGGCACAGGCACTAAACTCATCGTTTCCA  Jγ4(Jγ5)
Vγ1.3p-1: TACTACTGTGCCTACTGGGACTCT-------------TTACAC-----------------GGCAATCTGGTCTTTGGCACAGGCACTAAACTCATCGTTTCCA  (*)      24
Vγ1.3-3 : TACTACTGTGCCTACTGGGACTC---------------GGGG---------------------AATCTGGTCTTTGGCACAGGCACTAAACTCATCGTTTCCA           18

          Y  Y  C                                                                 F  G  T  G
Vγ1.3p  : TACTACTGTGCCTACTGGGACTCT                           TCTGGTTATTACTACAAAGTGTTTGGCACCGGTACAAAGCTCATCGTGTCAG  Jγ3
Vγ1.3-2 : TACTACTGTGCCTACTGGGA---------------------------------TGGTTATTACTACAAAGTGTTTGGCACCGGTACAAAGCTCATCGTGTCAG           21
Vγ1.3-4 : TACTACTGTGCCTAC-----------------------GGTCG-------------------------AGTGTTTGGCACCGGTACAAAGCTCATCGTGTCAG            6


          Y  Y  C                                                                 F  G  T  G
Vγ1.4   : TACTACTGTGCCTACTGGGACTCT                                    GGCAATCTGGTCTTTGGCACAGGCACTAAACTCATCGTTTCCA  Jγ4(Jγ5)
Vγ1.4-1 : TACTACTGTGCCTACTGGGACT----------------TTGAG-----------------GGCAATCTGGTCTTTGGCACAGGCACTAAACTCATCGTTTCCA           21
Vγ1.4-4 : TACTACTGTGCCTACTGGGAC---------------CGCGACGGAG------------------ATCTGGTCTTTGGCACAGGCACTAAACTCATCGTTTCCA           21
Vγ1.4-5 : TACTACTGTGCCTACTGGG--------------------GGAC----------------------TCTGGTCTTTGGCACAGGCACTAAACTCATCGTTTCCA           12

          Y  Y  C                                                                 F  G  T  G
Vγ1.4   : TACTACTGTGCCTACTGGGACTCT                           TCTGGTTATTACTACAAAGTGTTTGGCACCGGTACAAAGCTCATCGTGTCAG  Jγ3
Vγ1.4-2 : TACTACTGTGCCTACTGGGACTCT--------------CTCGC----------------TTACTACAAAGTGTTTGGCACCGGTACAAAGCTCATCGTGTCAG           24
Vγ1.4-3 : TACTACTGTGCCTACTGGGAC-----------------CCTC----------------ATTACTACAAAGTGTTTGGCACCGGTACAAAGCTCATCGTGTCAG           21
Vγ1.4-8 : TACTACTGTGCCTACTGGGACTCT--------------CGGC----------CTGGTTATTACTACAAAGTGTTTGGCACCGGTACAAAGCTCATCGTGTCAG           30

          Y  Y  C                                                                 F  G  T  G
Vγ1.4   : TACTACTGTGCCTACTGGGACTCT                             GACCACATGGATTAAATACTTCGGTACAGGAACTAAGCTAATTATCTCCG  Jγ1
Vγ1.4-6 : TACTACTGTGCCTACTGGGACT-----------------GG-------------------TGGATTAAATACTTCGGTACAGGAACTAAGCTAATTATCTCCG           18
Vγ1.4-7 : TACTACTGTGCCTACTGGGACTC------------------------------------ATGGATTAAATACTTCGGTACAGGAACTAAGCTAATTATCTCCG           18
Vγ1.4-9 : TACTACTGTGCCTACTGGGACTCT--------------AGGA-------------------GGATTAAATACTTCGGTACAGGAACTAAGCTAATTATCTCCG           21


          Y  Y  C                                                                 F  G  T  G
Vγ1.5p  : TACTACTGTGCCTACTGGGACTCT                                    GGCAATCTGGTCTTTGGCACAGGCACTAAACTCATCGTTTCCA  Jγ4(Jγ5)
Vγ1.5p-1: TACTACTGTGCCTACTGGG------------------------------------------GCAATCTGGTCTTTGGCACAGGCACTAAACTCATCGTTTCCA  (*)      12

          Y  Y  C                                                                 F  G  T  G
Vγ1.5p  : TACTACTGTGCCTACTGGGACTCT                           TCTGGTTATTACTACAAAGTGTTTGGCACCGGTACAAAGCTCATCGTGTCAG  Jγ3
Vγ1.5p-2: TACTACTGTGCCTACTGGGACTCT----------------C--------------GTTATTACTACAAAGTGTTTGGCACCGGTACAAAGCTCATCGTGTCAG  (*)      24

          Y  Y  C                                                                 F  G  T  G
Vγ1.5p  : TACTACTGTGCCTACTGGGACTCT                             GACCACATGGATTAAATACTTCGGTACAGGAACTAAGCTAATTATCTCCG  Jγ1
Vγ1.5p-3: TACTACTGTGCCTACTG----------------------CG------------------ATGGATTAAATACTTCGGTACAGGAACTAAGCTAATTATCTCCG  (*)      14


          Y  Y  C                                                                 F  G  T  G
Vγ2.1   : TACTACTGCGCCTACTGGGACCCT                             GACCACATGGATTAAATACTTCGGTACAGGAACTAAGCTAATTATCTCCG  Jγ1
Vγ2.1-1 : TACTACTGCGCCTACTGGGAC------------------------------------ACATGGATTAAATACTTCGGTACAGGAACTAAGCTAATTATCTCCG           18
Vγ2.1-2 : TACTACTGCGCCTACTGGGACCCT-------------ATTTACCA--------------ATGGATTAAATACTTCGGTACAGGAACTAAGCTAATTATCTCCG           27


          Y  Y  C                                                                 F  G  T  G
Vγ3.1   : TACTACTGTGCGTACTGGTATTCATATCAAGGCAT                  GGATGCTGATGTTAAAATATTTGGAACTGGAACTAAGCTTATTGTTTCAG  Jγ2
Vγ3.1-1 : TACTACTGTGCGTACTGGTATTCATATCAAGGCAT------C------------------GATGTTAAAATATTTGGAACTGGAACTAAGCTTATTGTTTCAG           30
Vγ3.1-10: TACTACTGTGCGTACTGGTATTCATATCA-----------GCG-------------------TGTTAAAATATTTGGAACTGGAACTAAGCTTATTGTTTCAG           24

          Y  Y  C                                                                 F  G  T  G
Vγ3.1   : TACTACTGTGCGTACTGGTATTCATATCAAGGCAT                TCTGGTTATTACTACAAAGTGTTTGGCACCGGTACAAAGCTCATCGTGTCAG  Jγ3
Vγ3.1-2 : TACTACTGTGCGTACTGGTATTCATATCAAGGCAT------G---------------TATTACTACAAAGTGTTTGGCACCGGTACAAAGCTCATCGTGTCAG           33
Vγ3.1-3 : TACTACTGTGCGTACTGGTATTCATATC----------GAGAGGG--------------TTACTACAAAGTGTTTGGCACCGGTACAAAGCTCATCGTGTCAG           30
Vγ3.1-7 : TACTACTGTGCGTACTGGTATTCATATCA------------------------------TTACTACAAAGTGTTTGGCACCGGTACAAAGCTCATCGTGTCAG           24
Vγ3.1-9 : TACTACTGTGCGTACTGGTATTCATATC----------CGTTTAAG--------------TACTACAAAGTGTTTGGCACCGGTACAAAGCTCATCGTGTCAG           30
Vγ3.1-13: TACTACTGTGCGTACTGGTATTCATATCAAGG--------GGGC-------TCTGGTTATTACTACAAAGTGTTTGGCACCGGTACAAAGCTCATCGTGTCAG           39
Vγ3.1-15: TACTACTGTGCGTACTGGTATTCATATCAAGGCAT---CCGGCTC---------GGTTATTACTACAAAGTGTTTGGCACCGGTACAAAGCTCATCGTGTCAG           42
```

```
          Y  Y  C                                                                 F  G  T  G
Vγ3.1   : TACTACTGTGCGTACTGGTATTCATATCAAGGCAT                  GACCACATGGATTAAATACTTCGGTACAGGAACTAAGCTAATTATCTCCG  Jγ1
Vγ3.1-4 : TACTACTGTGCGTACTGGTATTCATATCAAGGCAT------------------------ATGGATTAAATACTTCGGTACAGGAACTAAGCTAATTATCTCCG            30
Vγ3.1-5 : TACTACTGTGCGTACTGGT--------------------CAGG----------------ATGGATTAAATACTTCGGTACAGGAACTAAGCTAATTATCTCCG            18
Vγ3.1-6 : TACTACTGTGCGTACTGGTATTCATATCAAGGCAT----ACAG--------------ACATGGATTAAATACTTCGGTACAGGAACTAAGCTAATTATCTCCG            36
Vγ3.1-8 : TACTACTGTGCGTACTGGTATTCAT------------------------------------GGATTAAATACTTCGGTACAGGAACTAAGCTAATTATCTCCG            18
Vγ3.1-12: TACTACTGTGCGTACTGGTATTCAT-------------CGGGTTC--------GACCACATGGATTAAATACTTCGGTACAGGAACTAAGCTAATTATCTCCG            33
Vγ3.1-14: TACTACTGTGCGTACTGGT----------------CCCCGACATCACAAGGGC--------GGATTAAATACTTCGGTACAGGAACTAAGCTAATTATCTCCG            30
Vγ3.1-16: TACTACTGTGCGTACTGGTATTCATATCAAGGC-------G-----------------CATGGATTAAATACTTCGGTACAGGAACTAAGCTAATTATCTCCG            30
Vγ3.1-17: TACTACTGTGCGTACTGGT-----------------GGGGAAAGACCG----------CATGGATTAAATACTTCGGTACAGGAACTAAGCTAATTATCTCCG            27

          Y  Y  C                                                                 F  G  T  G
Vγ3.1   : TACTACTGTGCGTACTGGTATTCATATCAAGGCAT                         GGCAATCTGGTCTTTGGCACAGGCACTAAACTCATCGTTTCCA  Jγ4 (Jγ5)
Vγ3.1-11: TACTACTGTGCGTACTGGTATTCATATCA-----------CA-------------------GCAATCTGGTCTTTGGCACAGGCACTAAACTCATCGTTTCCA            24


          Y  Y  C                                                                 F  G  T  G
Vγ4     : TACTACTGTGCCTACTGGCAGTCT                             GGATGCTGATGTTAAAATATTTGGAACTGGAACTAAGCTTATTGTTTCAG  Jγ2
Vγ4-1   : TACTACTGTGCCTACTGGCAGTC----------------GCGAC-----------------ATGTTAAAATATTTGGAACTGGAACTAAGCTTATTGTTTCAG            21
Vγ4-4   : TACTACTGTGCCTACTGGCAGTCT--------------GAAGAA-------------GCTGATGTTAAAATATTTGGAACTGGAACTAAGCTTATTGTTTCAG            27
Vγ4-7   : TACTACTGTGCCTACTGGCAGTC---------------CAGACTC---------------GATGTTAAAATATTTGGAACTGGAACTAAGCTTATTGTTTCAG            24

          Y  Y  C                                                                 F  G  T  G
Vγ4     : TACTACTGTGCCTACTGGCAGTCT                             GACCACATGGATTAAATACTTCGGTACAGGAACTAAGCTAATTATCTCCG  Jγ1
Vγ4-2   : TACTACTGTGCCTACTGGCAGTCT-----------------------------GACCACATGGATTAAATACTTCGGTACAGGAACTAAGCTAATTATCTCCG  (*)       25
Vγ4-3   : TACTACTGTGCCTACTGGCAGTCT--------------AGCGAC----------------TGGATTAAATACTTCGGTACAGGAACTAAGCTAATTATCTCCG            24
Vγ4-5   : TACTACTGTGCCTACTGGCAG-----------------ATCGACG----------CCACATGGATTAAATACTTCGGTACAGGAACTAAGCTAATTATCTCCG            27

          Y  Y  C                                                                 F  G  T  G
Vγ4     : TACTACTGTGCCTACTGGCAGTCT                                    GGCAATCTGGTCTTTGGCACAGGCACTAAACTCATCGTTTCCA  Jγ4 (Jγ5)
Vγ4-6   : TACTACTGTGCCTACTGGCAG-----------------CCAATCG-------------------ATCTGGTCTTTGGCACAGGCACTAAACTCATCGTTTCCA            28


          Y  Y  C                                                                 F  G  T  G
Vγ6     : TATTACTGTGCTTACTGGGAGAG                              GGATGCTGATGTTAAAATATTTGGAACTGGAACTAAGCTTATTGTTTCAG  Jγ2
Vγ6-1   : TATTACTGTGCTTACTGGGAG------------------TCCA---------------CTGATGTTAAAATATTTGGAACTGGAACTAAGCTTATTGTTTCAG            21
Vγ6-5   : TATTACTGTGCTTACTGG------------------TCCACCTTTA---------------ATGTTAAAATATTTGGAACTGGAACTAAGCTTATTGTTTCAG            21

          Y  Y  C                                                                 F  G  T  G
Vγ6     : TATTACTGTGCTTACTGGGAGAG                              GACCACATGGATTAAATACTTCGGTACAGGAACTAAGCTAATTATCTCCG  Jγ1
Vγ6-2   : TATTACTGTGCTTACTGGGAG------------------GTCC------------CCACATGGATTAAATACTTCGGTACAGGAACTAAGCTAATTATCTCCG            24

          Y  Y  C                                                                 F  G  T  G
Vγ6     : TATTACTGTGCTTACTGGGAGAG                            TCTGGTTATTACTACAAAGTGTTTGGCACCGGTACAAAGCTCATCGTGTCAG  Jγ3
Vγ6-3   : TATTACTGTGCTTACTGGGAGAG------------GCGTATTCGTCGT------------------AAAGTGTTTGGCACCGGTACAAAGCTCATCGTGTCAG            24

          Y  Y  C                                                                 F  G  T  G
Vγ6     : TATTACTGTGCTTACTGGGAGAG                                     GGCAATCTGGTCTTTGGCACAGGCACTAAACTCATCGTTTCCA  Jγ4 (Jγ5)
Vγ6-4   : TATTACTGTGCTTAC------------------------GCAT------------------------TGGTCTTTGGCACAGGCACTAAACTCATCGTTTCCA             6
Vγ6-6   : TATTACTGTGCTTACTGGGAGA------------------AGG------------------------TGGTCTTTGGCACAGGCACTAAACTCATCGTTTCCA            12
Vγ6-7   : TATTACTGTGCTTACT----------------------CGCAGCG-----------------------GGTCTTTGGCACAGGCACTAAACTCATCGTTTCCA             9


                                                                                  F  G  T  G
          Y  Y  C                                                     GGCAATCTGGTCTTTGGCACAGGCACTAAACTCATCGTTTCCA  Jγ4 (Jγ5)
Vγ1.6-1 : TACTACTGT  GCCTCCTGA----------------------------------------------------TTTGGCACAGGCACTAAACTCATCGTTTCCA  (*)        0
Vγ1.6-5 : TACTACTGT  GCCTACTGGGACTTTCTCACG----------------------------GGCAATCTGGTCTTTGGCACAGGCACTAAACTCATCGTTTCCA            24
Vγ1.6-8 : TACTACTGT  GCCTACTGGGACTCTATCGT-------------------------------CAATCTGGTCTTTGGCACAGGCACTAAACTCATCGTTTCCA            21
Vγ1.6-9 : TACTACTGT  GCCTACTGGGACTCG----------------------------------GGCAATCTGGTCTTTGGCACAGGCACTAAACTCATCGTTTCCA            18
Vγ1.6-13: TACTACTGT  GCCTACTGGGACTCCGCGC----------------------------------ATCTGGTCTTTGGCACAGGCACTAAACTCATCGTTTCCA            18
Vγ1.6-15: TACTACTGT  GCCTACTGGGACTCGAG-------------------------------------TCTGGTCTTTGGCACAGGCACTAAACTCATCGTTTCCA            15
Vγ1.6-16: TACTACTGT  GCCTACTGGGCCGACTC----------------------------------CAATCTGGTCTTTGGCACAGGCACTAAACTCATCGTTTCCA            18
Vγ1.6-17: TACTACTGT  GCCTACTGGGACACC----------------------------------GGCAATCTGGTCTTTGGCACAGGCACTAAACTCATCGTTTCCA            18
Vγ1.6-19: TACTACTGT  GCCTACTGGGACTCTCGCA-------------------------------GCAATCTGGTCTTTGGCACAGGCACTAAACTCATCGTTTCCA            21

                                                                                  F  G  T  G
          Y  Y  C                                            TCTGGTTATTACTACAAAGTGTTTGGCACCGGTACAAAGCTCATCGTGTCAG  Jγ3
Vγ1.6-2 : TACTACTGT  GCCTACTGGGACCGCGTACATCTA----------------TCTGGTTATTACTACAAAGTGTTTGGCACCGGTACAAAGCTCATCGTGTCAG            36
Vγ1.6-3 : TACTACTGT  GCCTACTGGGACTCTAGGCCGG-------------------CTGGTTATTACTACAAAGTGTTTGGCACCGGTACAAAGCTCATCGTGTCAG            33
Vγ1.6-6 : TACTACTGT  GCCTACTGGGAC----------------------------TCTGGTTATTACTACAAAGTGTTTGGCACCGGTACAAAGCTCATCGTGTCAG            24
Vγ1.6-7 : TACTACTGT  GCCTACTGGGACTCGTCG-------------------------------TACTACAAAGTGTTTGGCACCGGTACAAAGCTCATCGTGTCAG            21
Vγ1.6-11: TACTACTGT  GCCTACTATCGCACA-------------------------------TATTACTACAAAGTGTTTGGCACCGGTACAAAGCTCATCGTGTCAG            21
Vγ1.6-14: TACTACTGT  GCCTACTGGGACTCTGAGAGTGGA-------------------------TACTACAAAGTGTTTGGCACCGGTACAAAGCTCATCGTGTCAG            27

                                                                                  F  G  T  G
          Y  Y  C                                              GACCACATGGATTAAATACTTCGGTACAGGAACTAAGCTAATTATCTCCG  Jγ1
Vγ1.6-4 : TACTACTGT  GCCTACTGGGACTCTAGTGGGGTGG-------------------CCACATGGATTAAATACTTCGGTACAGGAACTAAGCTAATTATCTCCG            33
Vγ1.6-10: TACTACTGT  GCCTAC-------------------------------------------TGGATTAAATACTTCGGTACAGGAACTAAGCTAATTATCTCCG             9
Vγ1.6-18: TACTACTGT  GCCTACTGGGACTCGTC-------------------------GACCACATGGATTAAATACTTCGGTACAGGAACTAAGCTAATTATCTCCG            27
Vγ1.6-20: TACTACTGT  GCCTACTGGGACTCGA----------------------------------GGATTAAATACTTCGGTACAGGAACTAAGCTAATTATCTCCG            18

                                                                                  F  G  T  G
          Y  Y  C                                              GGATGCTGATGTTAAAATATTTGGAACTGGAACTAAGCTTATTGTTTCAG  Jγ2
Vγ1.6-12: TACTACTGT  GCCTACTGGGACTCCCCCCCCATTCA----------------GGATGCTGATGTTAAAATATTTGGAACTGGAACTAAGCTTATTGTTTCAG            36


                                                                                  F  G  T  G
          Y  Y  C                                              GACCACATGGATTAAATACTTCGGTACAGGAACTAAGCTAATTATCTCCG  Jγ1
Vγ3.4-1 : TACTACTGT  GCACGTTGGTATTATTATCAAGGCCGTGG----------------CACATGGATTAAATACTTCGGTACAGGAACTAAGCTAATTATCTCCG            36

                                                                                  F  G  T  G
          Y  Y  C                                            TCTGGTTATTACTACAAAGTGTTTGGCACCGGTACAAAGCTCATCGTGTCAG  Jγ3
Vγ3.4-2 : TACTACTGT  GCACGTTGGTATTATTATCAAGGCATAGAGGGAGGA----TCTGGTTATTACTACAAAGTGTTTGGCACCGGTACAAAGCTCATCGTGTCAG            48
Vγ3.4-6 : TACTACTGT  GCACGTTGGCATTATTATCAAGGCGAC-------------TCTGGTTATTACTACAAAGTGTTTGGCACCGGTACAAAGCTCATCGTGTCAG            39
Vγ3.4-9 : TACTACTGT  GCACGTTGGTATTATTATCAAAGGACC----------------------------------TTTGGCACCGGTACAAAGCTCATCGTGTCAG            18
Vγ3.4-14: TACTACTGT  GCCTACTGGGACTTCGGGG----------------------------ATTACTACAAAGTGTTTGGCACCGGTACAAAGCTCATCGTGTCAG            24
Vγ3.4-15: TACTACTGT  GCACGTTGGTATTATTATCAAGGCACCCGA----------TCTGGTTATTACTACAAAGTGTTTGGCACCGGTACAAAGCTCATCGTGTCAG            42
```

```
                                                                                     F  G  T  G
             Y  Y  C                                                   GGCAATCTGGTCTTTGGCACAGGCACTAAACTCATCGTTTCCA  Jγ4(Jγ5)
Vγ3.4-3 :  TACTACTGT  GCACGTTGGCTAT----------------------------------------ATCTGGTCTTTGGCACAGGCACTAAACTCATCGTTTCCA            12
Vγ3.4-5 :  TACTACTGT  GCACGTTGGTATTATTATCAAGGTCTCCGA---------------------AATCTGGTCTTTGGCACAGGCACTAAACTCATCGTTTCCA            30
Vγ3.4-7 :  TACTACTGT  GCACGTTGGTATTATTATCAAGGCGAC----------------------GGCAATCTGGTCTTTGGCACAGGCACTAAACTCATCGTTTCCA            30
Vγ3.4-11:  TACTACTGT  GCACGTTGGTATTATTATCAAGGCCGT----------------------GGCAATCTGGTCTTTGGCACAGGCACTAAACTCATCGTTTCCA            30
Vγ3.4-12:  TACTACTGT  GCACGTTGGTATTATT-------------------------------------ATCTGGTCTTTGGCACAGGCACTAAACTCATCGTTTCCA            15
Vγ3.4-13:  TACTACTGT  GCACGTTGGTATTATTATCGGCACAC-----------------------GGCAATCTGGTCTTTGGCACAGGCACTAAACTCATCGTTTCCA  (*)       29
Vγ3.4-16:  TACTACTGT  GCACGTTGGTATTATTATCAA----------------------------GGCAATCTGGTCTTTGGCACAGGCACTAAACTCATCGTTTCCA            24
Vγ3.4-17:  TACTACTGT  GCACGTTGGTATTATTATCAAGTCCCTCGC--------------------CAATCTGGTCTTTGGCACAGGCACTAAACTCATCGTTTCCA  (*)       31
Vγ3.4-19:  TACTACTGT  GCACGTTGGTATTATTATCGGAGGAGCAATCTGGC-------------------------CTTTGGCACAGGCACTAAACTCATCGTTTCCA            27
Vγ3.4-20:  TACTACTGT  GCACGTTGCTCCTTC-------------------------------------------------GGCACAGGCACTAAACTCATCGTTTCCA             6

                                                                                     F  G  T  G
             Y  Y  C                                            GGATGCTGATGTTAAAATATTTGGAACTGGAACTAAGCTTATTGTTTCAG  Jγ2
Vγ3.4-4 :  TACTACTGT  GCACGTTGGTATTATTATCAAGGCTCC-------------------GCTGATGTTAAAATATTTGGAACTGGAACTAAGCTTATTGTTTCAG            33
Vγ3.4-8 :  TACTACTGT  GCACGTTGGTATTATTATCAAGGCA-------------------ATGCTGATGTTAAAATATTTGGAACTGGAACTAAGCTTATTGTTTCAG            33
Vγ3.4-10:  TACTACTGT  GCACGTTGGTATTATTATCAAGAGCC-----------------------GATGTTAAAATATTTGGAACTGGAACTAAGCTTATTGTTTCAG  (*)       29
Vγ3.4-18:  TACTACTGT  GCACGTTGGTATTATTATCAAGGCATTGACGGGT-------------CTGATGTTAAAATATTTGGAACTGGAACTAAGCTTATTGTTTCAG            39
```

E

```
                                                                                                                     CDR3  (nt)
                                                   D                                    J

          Y  F  C                                                                   F  G  S  G
          TACTTCTGTGCCAGACAG                 TTGAGAACTTGGAGTAG               GAAACAGACAAGCTTGTATTTGGAAGTGGGATTGCTTTCTCAGTTGAACCAA
TRD2-1 : TACTTCTGTGCCAGACAG--------CT----------AGAACTTGGAGTAG-----CATCGG--------CAGACAAGCTTGTATTTGGAAGTGGGATTGCTTTCTCAGTTGAACCAA       36
TRD2-2 : TACTTCTGTGCCAGACAG------------------------CTTGGAGTA--AGCCCGCCTAAGA--AAACAGACAAGCTTGTATTTGGAAGTGGGATTGCTTTCTCAGTTGAACCAA       39
TRD2-3 : TACTTCTGTGCCAGACAG--------CCGG---------------GGAGTAG-----CCGGGG---------AGACAAGCTTGTATTTGGAAGTGGGATTGCTTCCTCAGTTGAACCAA       30
TRD2-4 : TACTTCTGTGCCAGACAG--------C-----------AGAACTTGGAGTAG-----CTTCG--------ACAGACAAGCTTGTATTTGGAAGTGGGATTGCTTTCTCAGTTGAACCAA  (*)  35
TRD2-5 : TACTTCTGTGCCAGACAG--------CAA------TTGAGAACTTGGAGTAG-------C----------ACAGACAAGCTTGTATTTGGAAGTGGGATTGCTTTCTCAGTTGAACCAA       36
TRD2-7 : TACTTCTGTGCCAGA--------------------------ACTTGGAGTA-------------------ACAGACAAGCTTGTATTTGGAAGTGGGATTGCTTTCTCAGTTGAACCAA  (*)  22
TRD2-8 : TACTTCTGTGCCAGACAG-----CATGGGCTAG----GAGAACTTGGAGTAG-------C---------AACAGACAAGCTTGTATTTGGAAGTGGGATTGCTTTCTCAGTTGAACCAA       42
TRD2-9 : TACTTCTGTGCCAGACAG---------C------------AACTTGGAGTA-------CCG------GAAACAGACAAGCTTGTATTTGGAAGTGGGATTGCTTTCTCAGTTGAACCAA       33
TRD2-10: TACTTCTGTGCCAGACAG--------CT----------AGAACTTGGAGTAG-----CATCGG--------CAGACAAGCTTGTATTTGGAAGTGGGATTGCTTTCTCAGTTGAACCAA       36
TRD2-11: TACTTCTGTGCCAGACAG----CATGGGCTAGGT-----GAACTTGGAGTAG-------T---------AACAGACAAGCTTGTATTTGGAAGTGGGATTGCTTTCTCAGTTGAACCAA       42
TRD2-12: TACTTCTGTGCCGGACAG-----CATGGGCTAG----GAGAACTTGGAGTAG------CT----------ACAGACAAGCTTGTATTTGGAAGTGGGATTGCTTTCTCAGTTGAACCAA       42
TRD2-13: TACTTCTGTGCCAGACAG----CATGGGCTAGGT-----GAACTTGGAGTA----------------GAAACAGACAAGCTTGTATTTGGAAGTGGGATTGCTTTCTCAGTTGAACCAA       42
TRD2-14: TACTTCTGTGCCAGACAG----CATGGGCTAGGT-----GAACTTGGAGTAG------CT----------ACAGACAAGCTTGTATTTGGAAGTGGGATTGCCTTCTCAGTTGAACCAA       42
TRD2-15: TACTTCTGTGCCAGACAG-----CATGGGCTAG----GAGAACTTGGAGTAG------C----------AACAGACAAGCTTGTATTTGGAAGTGGGATTGCTTTCTCAGTTGAACCAA       42
TRD2-16: TACTTCTGTGCCAGACAG---------C--------TGAGAACTTGG----------------------ACAGACAAGCTTGTATTTGGAAGTGGGATTGCTTTCTCAGTTGAACCAA       27
TRD2-18: TACTTCTGTGCCAGACAG--------CA-------TTGAGAACTTGGAGTAG----CGCGCGT--------CAGACAAGCTTGTATTTGGAAGTGGGATTGCTTTCTCAGTTGAACCAA  (*)  40
```

Supplementary Figure S5 Combinatorial and junctional diversity of duck TCR genes expressed in the thymus, TCRα (A), TCRδ (B), TCRβ (C), TCRγ (D) and TCRδ2 (E). The nucleotide sequences of the V(D)J junctions of all unique TCR clones are shown. The germline sequences of the 3'-end of the V, D and J segments are shown above the cDNA clones and in bold. Conserved sequences of the germline V and J segments are underlined. Nucleotides shaded in the junctions are putative P nucleotides. Gaps are indicated by dashes. The CDR3 is defined as the region between the J region–encoded FGXG motif and the nearest preceding V region–encoded cysteine, as described in the IMGT unique numbering for V-DOMAIN. The number on the right-hand side of each cDNA clone indicates the nucleotide length of the CDR3 region. The asterisk indicates nonfunctional transcripts.

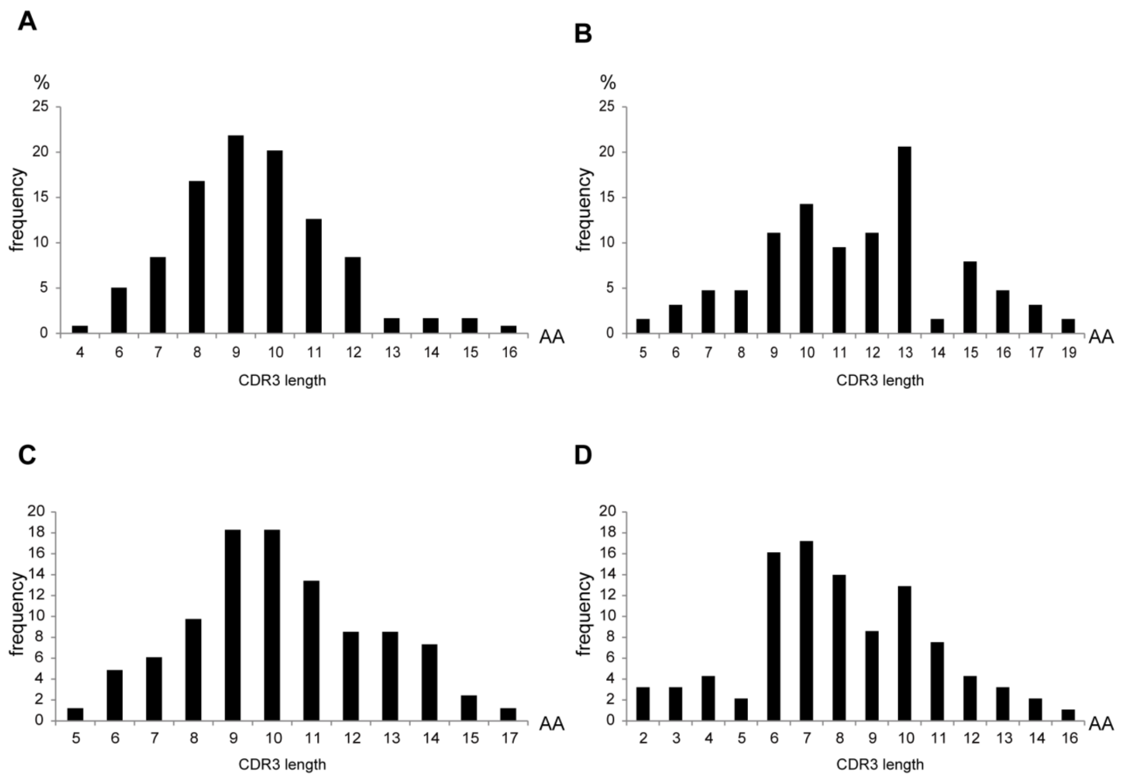

Supplementary Figure S6 CDR3 length distributions of duck TCR cDNA clones, TCRα (A), TCRδ (B), TCRβ (C) and TCRγ (D). The *x*-axis shows the amino-acid length of CDR3, and the *y*-axis indicates the percentages of CDR3 sequences with the indicated lengths.

Supplementary Table S1 Primer Sequences

Name	Sequence	Purpose
TRBF	5'-CAGATGGGTCTCCCGTAGAA-3'	BAC screening for TCRβ locus
TRBR	5'-CCCACCACTGTGAATGTGAG-3'	
TRGF	5'-ATGGTCTGGTGGCAGTAAGG-3'	BAC screening for TCRγ locus
TRGR	5'- TGGCCATGGATTGTTCTGTA-3'	
TRAF	5'-CAAATATGCAGCCTTGCTGA-3'	BAC screening for TCRα/δ locus
TRAR	5'-TTCCTCCTTCCTCTCCCAAT-3'	
TRAF2	5'-GGGATGCACTTCAACCAGAT-3'	BAC screening for the overlap clone
TRAR2	5'-CCCTGTGCAAACCCTAGAAA-3'	
TRD2F	5'-GCAGGCATTTCTTTCTCTCG-3'	BAC screening for TCRδ2 locus
TRD2R	5'-ACCCAAATTGTCTGCACTCC-3'	
NotI-d(T)$_{18}$	5'-AACTGGAAGAATTCGCGGCCGCAGGAATTTTTTTTTTTTTTTTTT-3'	-
RT-P1	5'-AACTGGAAGAATTCGCGGCC-3'	-
RT-P2	5'-AAGAATTCGCGGCCGCAGGAA-3'	-
Cb3F1	5'-CCCCGACACCCTCAATTTAG-3'	3'RACE for Cβ (1st round RCR)
Cb3F2	5'-AAGGGGTGGGTACAGATGAG-3'	3'RACE for Cβ (nested RCR)
Cg3F1	5'-TCTGCATTGTCATTTTAAAG-3'	3'RACE for Cγ (1st round RCR)
Cg3F2	5'-GAGCGGATGTTCTACATTTC-3'	3'RACE for Cγ (nested RCR)
Ca3F1	5'-CACCACGGAGCTGAACACTG-3'	3'RACE for Cα (1st round RCR)
Ca3F2	5'-CTGACAGCGCCTTGTTCTTC-3'	3'RACE for Cα (nested RCR)
Cd3F1	5'-CTTGTTTGGCAAGGAACTTC-3'	3'RACE for Cδ (1st round RCR)
Cd3F2	5'-TTTGCAACACCACAGACATC-3'	3'RACE for Cδ (nested RCR)
C2d3F1	5'-TGGAGAAGCCTGCAATGTTC-3'	3'RACE for Cδ2 (1st round RCR)
C2d3F2	5'-TTTGCTGCAGCCCCTGAAGG-3'	3'RACE for Cδ2 (nested RCR)

B1gsp1	5'-CTTTCCCACAGAGAATAAG-3'	5'RACE for TCRβ1 (reverse transcription)
B2gsp1	5'-CAAAATGAGCATGATGTACA-3'	5'RACE for TCRβ2 (reverse transcription)
Bgsp2	5'-TCGACTGGTCAGTGAATAAG-3'	5'RACE for both TCRβ1 and β2 (1st round RCR)
Bgsp3	5'-TGTGGAAGTCTCATCTGTAC-3'	5'RACE for both TCRβ1 and β2 (nested RCR)
Ggsp1	5'-TGGGGAGTTTAAAATTCTC-3'	5'RACE for TCRγ (reverse transcription)
Ggsp2	5'-GTGCTGACTGAGTACTTATC-3'	5'RACE for TCRγ (1st round RCR)
Ggsp3	5'-TGCAGGATTTCAGAGTTCTC-3'	5'RACE for TCRγ (nested RCR)
Agsp1	5'-CCCAGCTGTGTGAAAGACAG-3'	5'RACE for TCRα (reverse transcription)
Agsp2	5'-ATCCCTGTTACACAGACAGT-3'	5'RACE for TCRα (1st round RCR)
Agsp3	5'-TGCTTCCTGTTTATTCTCAG-3'	5'RACE for TCRα (nested RCR)
Dgsp1	5'-TGGCTGGAAAATTTTTGTTC-3'	5'RACE for TCRδ (reverse transcription)
Dgsp2	5'-CACATTCACCACTTTAATAG-3'	5'RACE for TCRδ (1st round RCR)
Dgsp3	5'-GTCCCCTCTGATGTCATAAC-3'	5'RACE for TCRδ (nested RCR)
CbpF	5'-TTTTCCCCATCAAAGCAAGA-3'	Southern blotting probe for Cβ
CbpR	5'-AGCATCACCATAGATGAATC-3'	
Vb3pF	5'-AACAGTCGCCAGACACAGTC-3'	Southern blotting probe for VβIII
Vb3pR	5'-GCACTTTCAGCACAGTAATA-3'	
Vb2pF	5'-AATCACCAGGCACAGTTGTC-3'	Southern blotting probe for VβII
Vb2pR	5'-GTGCCTGAGTCATTAAGTAG-3'	
CgpF	5'-GCAGACGAAGCATAAAGATC-3'	Southern blotting probe for Cγ
CgpR	5'-ATGGGGAGTTTAAAATTCTC-3'	
Vg1pF	5'-ACACGTCTGCAATGTCATTT-3'	Southern blotting probe for VγI
Vg1pR	5'-GTAGGCACAGTAGTAGATAG-3'	
Vg6pF	5'-TGCGCGGATCGACTGCCACT-3'	Southern blotting probe for VγVI
Vg6pR	5'-CCCAGTAAGCACAGTAATAG-3'	

CapF	5'-CAGGCTGACCACTAAAGATG-3'	Southern blotting probe for Cα
CapR	5'-CATCCCTGTTACACAGACAG-3'	
Va1pF	5'-TCACCCAGGAAGAAGGACAAG-3'	Southern blotting probe for VαI
Va1pR	5'-GTACAAGGCGCTGTCAGAAAG-3'	
Va3pF	5'-CAGCCTTGTTGGCACTGTCAG-3'	Southern blotting probe for VαIII
Va3pR	5'-GAGAAGCACCTGGGAGTTCTC-3'	
CdpF	5'-CTGGCCCAGAAGTTGTTCTG-3'	Southern blotting probe for Cδ
CdpR	5'-CCACCACCTTTTCTGATGAC-3'	
Vd2pF	5'-GGCAGGCGTTTGGGCTCAGAG-3'	Southern blotting probe for VδII
Vd2pR	5'-GAGCTCAGGGACAGAGAAGAC-3'	
Vd5pF	5'-CGCAGCCAAGGATTGTGGAG-3'	Southern blotting probe for VδV
Vd5pR	5'-GCCATGCAAAAGCAGAAATG-3'	
D2cpF	5'-CCAAGCAGACAAGAATATTC-3'	Southern blotting probe for Cδ2
D2cpR	5'-TTGCAAACTTTCACTGTCAC-3'	
D2vpF	5'-TTTGCTGCAGCCCCTGAAGG-3'	Southern blotting probe for VHδ
D2vpR	5'-TCCAAGTTCTCAATCTTTAG-3'	
EF1a1F	5'-GAGCCACCTTACAGCCAGAAGAGATA-3'	Quantitative real-time PCR for *EF1a1*
EF1a1R	5'-GATTGGCACAAAAGCTACAGTGTCTG-3'	
TRAqF	5'-CCTTCGGTCTACAGGCTGAC-3'	Quantitative real-time PCR for TCRα
TRAqR	5'-TGTTTCCACCTCCACAACAG-3'	
TRBqF	5'-AAGAGCAAAGCCACACTGGT-3'	Quantitative real-time PCR for TCRβ
TRBqR	5'-CCCTTCTGTCCTCTCAGCAC-3'	
TRGqF	5'-CTGGAAACAGCACCGTTTTT-3'	Quantitative real-time PCR for TCRγ
TRGqR	5'-GGGAGCTTAGCTGGAGTCCT-3'	
TRDqF	5'-GCAAATGTTTGCAACACCAC-3'	Quantitative real-time PCR for TCRδ

TRDqR	5'-TTTTTGCCAGCAGGACTCTT-3'	
TRD2qF	5'-TTGGCAAGGACATTCTACCC-3'	Quantitative real-time PCR for TCRδ2
TRD2qR	5'-CCCCTTTGCATCTGGTTTTA-3'	
TRD2VH	5'-TGGAGAAGCCTGCAATGTTC-3'	Junctional diversity of TCRδ2 transcripts
TRD2C	5'-TGGGACTCAAAGTTCTACAG-3'	

Supplementary Table S2 Accession numbers for sequences used in phylogenetic analyses

Figure	Accession number
Fig. 3A	**Human**: A22: DQ097920, A17: DQ097914, A6: X58747, A27: DQ341447, A35: DQ097935, A25: DQ097923, A30: X58768, A34: DQ097934, A10: DQ097904, A21: U50404, A24: M17661, A20: X70305, A41: DQ097943, A36-D7: X61070, A23-D6: D13071, A29-D5: M17664, A1.1: X04939, A14: S51029, A19: Z46641, A38-D8: D13074, D1: AY357942, A9.2: X57531, A40: DQ097942, A16: DQ097913, A3: X57534, A8.4: D13077, A4: M17663, A26.1: L06886, A2: DQ097917, D2: S24406, D3: M23326<br><br>**Mouse**: A4: L47342, A13: M38102, A21-D12: M94080, A11: DQ340292, A10: X57397, A5.4: M38681, A3.1: X02967, A14.3: L77149, A7: X56719, A1: M22604, AVD16D: M16118, D2.2: M37280, A15-D6: M37599, A6: M34200, A12.3: M38680, A9: M33586, A17: X60319, A2: X03760, D4: M23545, D5: X12729<br><br>**Cow**：013: D90013, 015: D90015, 012: D90012, 011: D90011, D113: D16113, D116: D16116, 014: D90014, 017: D90017, 016: D90016<br><br>**Sheep**: A622: M55622, D3S2: Z12996, D5: Z12995, A35: U78035, D2: AJ005904, D4: AJ005906, D6: AJ005908, D7: AJ809501, D1S1: Z12989<br><br>**Rabbit**: D5: D38121, D1: D26555, A885: M12885, D4: D38120<br><br>**Chicken**: A2: Scaffold AADN03011072.1:23-316, A1: 27:807845-808144, D1: 27:428976-429278<br><br>**Finch:** 066: 27:3077771-3078064, 164: 27:3067882-3068181, 500: 27:3048926-3049222, 516: 27:3033229-3033528, 495: 27:3054225-3054501, 497: 27:3051398-3051700<br><br>**Parrot**: A3: AGAI01023412, A1: AGAI01024880, A2: AGAI01025999, D5: AGAI01011509, D4: AGAI01044297, D6: AGAI01045807<br><br>**Grouse**: GW705681<br><br>**Turkey**: A03: ADDD01149483, A02: ADDD01152688, A01: ADDD01150225, D5: ADDD01052097, D4: ADDD01147795
Fig. 3B	**Human**：B12.1：X07224, B14: X06154, B11.3：X58797, B2: M64351, B21.1: L27608, B23: L27614, B13: U03115, B5: U03115, B9: M27380, B15: U03115, B3.1: U07977, B7.2：U07975, B19: U48260, B6.9: X61447, B10.3: U17047, B25.1: L27610, B27: U66061, B24.1: L27612, B28: U08314,

	B30: L06893, B20.1: X72719, B29.1: M13847, B4.3: X58812<br><br>**Mouse**：B16: L29434, B14: AE000664, B3: AE000663, B21: X16691, B24: M61184, B23: X59150, B26: K02548, B12: M30881, B17: AE000664, B4: X56725, B19: AJ249821, B13.3: M15616, B10: X16694, B29: X00696, B31: X03277, B20: M11859, B30: X16695, B1: X01642<br><br>**Rabbit**：B8: BAA04245, B11: BAA04248, B9: BAA04246, B7S1: BAA04241, B1: AAA31472, B5: BAA04239, B10: BAA04247, B2: M13895, B6: BAA04240<br><br>**Cow**：B125: PQ0061, B129: PQ0065, B126: PQ0062, B122: JQ0473, B123: PQ0060, B124: PQ0059<br><br>**Sheep**：B8S1: AAB88433, B6S1: AAB88431, B10S1: AAB88434, B1S5: AAB88425, B22S1: AAB88440, B7S1: AAB88432, B17S1: AAB88439, B13S1: PQ0068, B12S1: AAB88435, B15S1: AAB88438, B3S1: AAB88427, B2S1: AAB88426, B4S1: AAB88430<br><br>**Chicken**：B2: EF554773, B2S2: AAA62753, B1S1: B36198, B1: M37798<br><br>**Axolotl:** B3: L29423, B2: L33269, B1.1: L33258, B5: L33337, B6: L33400, B7.1: L33723, B11: AF324475, B4: L29422, B13: AF324477, B10: AF324474, B12: AF324476, B8: L33786, B9: L33787<br><br>**Frog**：B1S1: U60425, B9: U60431, B2: U60427, B3: U60432, B4: U60428, B6: U60434, B8: U60435, B5: U60424, B7: U60430, B10: U75994
Fig. 3C	**Human**：G2: M13429, G9: M27335 , G3: S60779, G4: S60780<br><br>**Mouse**：G6: M13338, G7: AF037352, G1: Z22847, G3: AF037352, G4: M13336<br><br>**Cow**：G5S6: D16129, G5S16: D16133, G5S11: D16126, G5S7: D16130, G1S1: D16119, G1S3: D16131, G6: AY560834, G3S2: U73187, G3S1: U73186, G4S1: U73188<br><br>**Sheep**：G5S2: Z13006, G5S1: Z13005, G1S1: Z12998, G2S2: Z13000, G2S1: Z12999, G2S3: Z13001, G2S4: Z13002, G6S1: Z13007, G4S1: Z13004, G3S1: Z13003<br><br>**Opossum**：G2: 6:283928450-283928153, G1.1: 6:283942345-283942040, G1.2: 6:283934927-283934623, G1.3: 6:283921739-283921428, G1.4: 6:283915397-283915042, G1.5: 6:283907901-283907590, G4: 6:283884333-283884026, G3.1: 6:283888100-283887792, G3.2:

	6:283879394-283879086<br><br>**Rabbit**：G1S4: D38139, G1S1: D38135, G1S3: D38138, G1S2: D38137, G2S1: D38142<br><br>**Platypus**：G1.1: AAY82100, G1.2: AAY82093, G1.3: AAY82109, G3.1: AAY82120, G3.2: AAY82094, G2.3: AAY82114, G2.1: AAY82119, G2.2: AAY82110<br><br>**Chicken**：G1S5: U78213, G1S4: U78212, G1S8: U78216, G1S3: U78210, G2S8: U78226, G2S7: U78225, G2S9: U78227, G3S4: U78231, G3S8: U78235, G3S3: U78230<br><br>**Frog**：G1: AF440824, G13: AF440826, G8: AF440827 , G14: AF440822

Supplementary Table S3 Similarity ranges of new TRV genes identified from cDNA analysis to genomic sequences in each of the subgroups

New genes identified from cDNA analysis		Range
**TRAV**	Vα1.2	87.4% ~ 96.8%
	Vα1.3	86.8% ~ 96.5%
	Vα1.4	87.5% ~ 96.8%
	Vα1.5	87.5% ~ 96.9%
	Vα2.5	80.6% ~ 96.5%
	Vα2.6	77.8% ~ 96.5%
	Vα2.7	78.9% ~ 95.4%
	Vα2.8p	79.4% ~ 91.8%
	Vα2.9	79.9% ~ 95.7%
	Vα2.10	79.2% ~ 93.3%
	Vα2.11	77.8% ~ 96.5%
	Vα2.12	81.0% ~ 91.8%
	Vα2.13	79.2% ~ 96.1%
	Vα2.14	78.4% ~ 94.7%
	Vα2.15	81.2% ~ 94.7%
	Vα2.16	80.6% ~ 91.8%
	Vα2.17	81.9% ~ 96.4%
	Vα2.18	81.4% ~ 96.4%
	Vα2.19	81.6% ~ 93.3%
	Vα2.20	80.9% ~ 93.6%
	Vα3.5	81.2% ~ 94.6%
	Vα3.6	79.3% ~ 94.3%

	Vα3.7	76.4% ~ 95.3%
	Vα3.8	79.7% ~ 93.2%
	Vα3.9	78.6% ~ 95.7%
	Vα3.10	76.4% ~ 95.7%
	Vα3.11p	80.1% ~ 93.2%
	Vα3.12	79.3% ~ 94.6%
	Vα3.13	79.3% ~ 96.8%
	Vα3.14	77.2% ~ 96.8%
	Vα3.15	80.6% ~ 91.8%
	Vα3.16	79.1% ~ 97.1%
	Vα3.17	80.1% ~ 95.3%
	Vα3.18	77.2% ~ 95.3%
	Vα3.19	79.3% ~ 96.8%
	Vα3.20	78.3% ~ 96.8%
	Vα3.21	80.4% ~ 96.8%
	Vα3.22	81.2% ~ 92.1%
	Vα3.23	80.1% ~ 93.9%
	Vα3.24	80.1% ~ 96.8%
**TRDV**	Vδ2.15	77.4% ~ 94.1%
	Vδ2.16	76.9% ~ 95.2%
	Vδ2.17	78.5% ~ 96.0%
	Vδ2.18	75.4% ~ 89.6%
	Vδ2.19	76.2% ~ 93.2%
	Vδ2.20	75.1% ~ 91.8%

**TRBV**	Vβ3.6	91.8% ~ 95.0%
	Vβ3.7	91.4% ~ 95.3%
**TRGV**	Vγ1.6	81.0% ~ 93.3%
	Vγ3.4	78.9% ~ 89.6%
